# Photochemical Aerobic Upcycling of Polystyrene Plastics via Indium Salt‐Mediated Synergistic Catalysis

**DOI:** 10.1002/cssc.202502759

**Published:** 2026-03-03

**Authors:** Lydia Lavrenti, Christos Papagkikas, Olga G. Mountanea, Christoforos G. Kokotos

**Affiliations:** ^1^ Laboratory of Organic Chemistry Department of Chemistry National and Kapodistrian University of Athens Panepistimiopolis Athens Greece

**Keywords:** aerobic oxidation, metal halides, photocatalysis, photochemical upcycling, polystyrene

## Abstract

The photochemical upcycling of polystyrene (PS) represents a sustainable method for the transformation of plastic waste into added‐value chemical feedstock. Herein, we propose a convenient and efficient photochemical catalytic system that integrates InBr_3_ catalysis with bromine radical sources, such as tosyl bromide and *N*‐bromosuccinimide (NBS). This approach enables the effective depolymerization of PS under mild conditions, providing benzoic acid in high yields. The protocol exhibited broad substrate tolerance, efficiently converting a variety of commercial and everyday PS‐based materials and was also successfully applied to a 1‐gram reaction. Comprehensive mechanistic investigations were performed, while applications to generate a series of value‐added compounds of potential pharmaceutical and industrial interest are highlighted. This study offers a practical method for the photochemical upcycling of PS waste through bromine radical‐mediated depolymerization.

## Graphical abstract

This study presents a practical aerobic photochemical strategy for the upcycling of polystyrene waste, combining bromine radical mediation with InBr_3_ catalysis. The approach efficiently depolymerizes diverse PS materials under mild conditions, yielding benzoic acid and enabling downstream production of value‐added compounds.

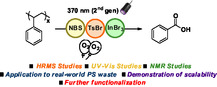

## 1 Introduction

Polymers are macromolecules composed of repeating subunits and are found in both natural systems (e.g., cellulose, DNA) and synthetic materials, including plastics [1, 2]. Owing to their low cost, durability, and lightweight nature, plastics experienced rapid industrial expansion after World War II [3, 4], particularly in the form of single‐use plastics (SUPs), which are now ubiquitous across healthcare, agriculture, and construction sectors [5]. Global plastic production reached approximately 7.800 million tonnes (Mt) between 1950 and 2015 [4], growing at an average annual rate of 9% [6], and is projected to increase substantially in the coming decades [4, 7]. This growth has led to severe environmental consequences, as more than 80% of plastic waste is improperly managed [4, 8], resulting in persistent pollution, ecosystem damage, and human exposure to micro‐ and nanoplastics [9]. The COVID‐19 pandemic further exacerbated plastic waste generation [10], and projections indicate that plastics could become a major contributor to CO_2_ emissions [11] and global waste accumulation by 2050 [12]. Among different plastic categories, polystyrene (PS), classified under recycling code 6, is a widely used synthetic polymer, with applications ranging from food packaging to protective packaging for electronics and fragile goods [13]. However, despite its versatility, polystyrene remains a material of environmental concern, due to its persistence and the challenges it poses for recycling [14]. Current plastic waste management methods—landfilling, incineration, and mechanical recycling—each present distinct drawbacks [15, 16]. Landfilling can contaminate soil and groundwater, while incineration emits greenhouse gases and toxic substances [8, 15, 16]. Mechanical recycling, though widely used, degrades plastic quality over repeated cycles and is limited to clean, single‐type materials [3, 15]. In contrast, chemical upcycling offers a more sustainable solution by converting mixed or contaminated plastics into high‐value products [15], helping reduce emissions and advance a circular economy [3, 8, 10, 15, 17, 18]. Among these, photocatalysis stands out as a milder alternative [16].

Photochemistry, the use of light to drive organic transformations, has gained significant attention over the past decade, since it provides milder and more sustainable alternatives to traditional chemical reactions [19, 20, 21, 22, 23, 24]. Since the pioneering experiments of Ciamician using sunlight on rooftops [25], photochemistry has offered solutions to previously challenging synthetic problems, particularly in the activation of strong C—H bonds [19, 20, 21, 22, 23, 24]. Photocatalysis, an environmentally friendly and cost‐effective subset of photochemistry, enables efficient plastic upcycling by converting light into chemical energy to activate substrates under mild conditions, avoiding harsh reagents or high energy input. Among the main mechanistic pathways, Hydrogen Atom Transfer (HAT) is a key process in both chemical and biological systems, enabling the cleavage of C(sp^3^)–H bonds through the simultaneous transfer of a proton and an electron (H• ≡ H^+^ + e^−^) from a hydrogen donor to an acceptor, or abstractor. HAT processes can occur indirectly (*i*‐HAT), where the hydrogen abstractor is generated through a single electron transfer (SET) from an excited photocatalyst, or directly (*d*‐HAT), where the excited photocatalyst itself abstracts the hydrogen [22]. Additionally, under certain coordination environments, light‐induced homolytic cleavage of metal–ligand bonds produces radicals capable of initiating hydrogen atom transfer (HAT) from substrates [26]. Most photocatalysts for plastic upcycling are semiconductors that work through light absorption, charge transfer and separation, and surface redox reactions [27, 28]. Plastic oxidation is less energy‐demanding than water oxidation, allowing use of visible light [3, 28]. In polystyrene (PS), low C–H bond energy (˜85 kcal/mol) enables hydrogen atom transfer (HAT) to form carbon‐centered radicals, which react with oxygen or reactive species to create alkoxy radicals [16, 29]. These undergo *β*‐scission, breaking polymer chains into carbonyl and alkyl radicals, which form benzoic acid, acetophenone, or additional carbonyls. Light‐driven photocatalysis also induces chain scission, branching, cross‐linking, and oxidation, selectively activating inert bonds and accelerating depolymerization [16, 29].

**SCHEME 1:**
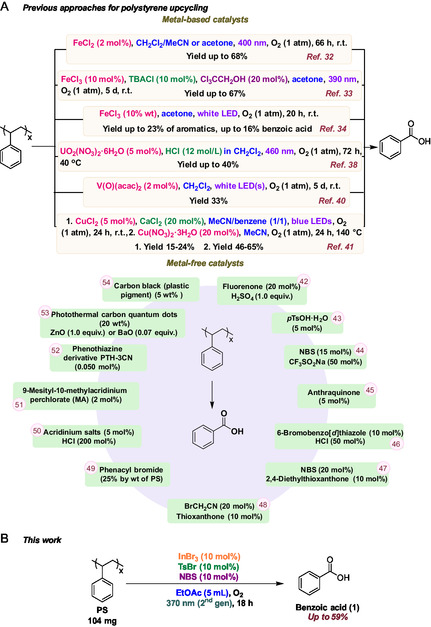
(A) Metal‐based and metal‐free photochemical approaches for upcycling polystyrene to benzoic acid. (B) This work.

Photochemical degradation of polystyrene (PS) has been explored since the 1980s [30, 31], but early studies were limited by the lack of suitable catalysts and mechanistic understanding. Renewed interest has emerged in recent years, particularly following the recognition that PS can be selectively converted into benzoic acid (PhCO_2_H), a high‐value industrial chemical. As a result, numerous photocatalytic protocols have been reported since 2021 (Scheme 1A). Several groups have demonstrated that simple iron salts can catalyze the aerobic photochemical oxidation of PS to benzoic acid [32, 33, 34]. For example, Zhang developed a catalytic system using FeCl_3_, in combination with tetrabutylammonium chloride (TBACl) and 2,2,2‐trichloroethanol under UV light, affording benzoic acid in yields up to 67% [33]. Subsequent studies revealed that halogen radicals can modulate product selectivity, with Cl^•^ favoring benzoic acid formation and Br^•^ leading to mixtures of benzoic acid and acetophenone [35]. Beyond iron catalysis, alternative metal‐based photocatalytic approaches have been explored, like a two‐step strategy involving initial functionalization of Styrofoam, followed by FeCl_3_‐catalyzed depolymerization to yield functionalized styrenes [36], a tandem UV‐driven process converting PS into aromatic intermediates using AlCl_3_ [37], uranyl or vanadium complexes [38, 39, 40], and a copper‐based LMCT photocatalytic system employing CuCl_2_ and CaCl_2_, enabling oxidative cleavage of PS waste to benzoic acid and oxidized styrene oligomers in up to 65% yield through an one‐pot, two‐step protocol [41].

In parallel with metal‐based systems, metal‐free photochemical strategies for polystyrene (PS) upcycling have rapidly emerged as viable alternatives. Early examples include fluorenone‐mediated partial oxidation of PS to benzoic acid under blue light [42], acid‐catalyzed photochemical oxidation yielding mixtures of benzoic acid, acetophenone, and formic acid [43], and HAT‐based approaches employing *N*‐bromosuccinimide‐derived radicals for the conversion of real‐world PS waste into aromatic products [44]. Subsequent studies expanded the scope and efficiency of metal‐free photocatalysis. Anthraquinone was shown to promote PS oxidation under UV irradiation and air, with downstream derivatization to pharmaceutically relevant products [45], while visible‐light‐mediated benzylic C(sp^3^)–H oxidation using organic photosensitizers was adapted to PS upcycling, achieving moderate benzoic acid yields [46]. Building on halogen‐radical‐mediated approaches, our group recently reported two synergistic *i*‐HAT‐based metal‐free photocatalytic protocols for PS upcycling [47, 48]. These systems employed thioxanthone‐based photocatalysts under UVA irradiation to generate bromine radicals in situ, enabling selective conversion of PS and PS‐derived materials into benzoic acid in yields up to 54%, with demonstrated scalability, catalyst recyclability, and favorable green metrics [47, 48]. Complementary bromine‐radical strategies have also been reported, including the use of phenacyl bromide as an “all‐in‐one” photo‐HAT reagent that generates multiple reactive species in situ [49], while acridinium salts have also emerged as powerful visible‐light photocatalysts [50, 51]. Other organic photocatalysts, including phenothiazine‐derived systems operating via consecutive photoinduced electron transfer (conPET), have demonstrated efficient and scalable PS degradation under mild conditions [52]. Beyond purely photochemical pathways, photothermal strategies have recently been introduced, wherein carbon‐based materials convert light into localized heat to enable polymer depolymerization [53, 54], yielding monomers or aromatic products.

In this study, we report a fundamentally distinct strategy for the photochemical upcycling of polystyrene that integrates bromine‐radical‐mediated C–H activation with unprecedented indium(III) bromide photocatalysis. In contrast to previously reported metal‐free *i*‐HAT systems and iron‐, copper‐, or halogen‐only photocatalytic approaches, this work introduces InBr_3_ as a dual‐function photocatalyst and bromine‐radical activator, enabling efficient PS depolymerization under mild conditions (Scheme 1B). The synergistic combination of InBr_3_ with readily available bromine radical sources, including tosyl bromide and *N*‐bromosuccinimide (NBS), provides a new mechanistic manifold for selective PS oxidation that is not accessible in our earlier metal‐free protocols or existing metal‐based systems. The method exhibits broad substrate generality across diverse real‐world PS materials, improved operational simplicity, and excellent scalability, as demonstrated by gram‐scale reactions. Detailed mechanistic investigations using HRMS, UV–Vis, and NMR spectroscopy elucidate the role of indium in the photochemical activation process. Finally, the direct conversion of PS‐derived benzoic acid into structurally diverse, value‐added compounds highlights the synthetic utility and circular‐economy relevance of this approach, positioning the present study as a significant advance beyond prior photocatalytic PS upcycling methodologies.

## 2 Results and Discussion

We initiated our investigation inspired by the work of Zeng, who employed a catalytic system based on FeCl_3_, tetrabutylammonium chloride (TBACl), and trichloroethanol under Kessil 390 nm irradiation under an oxygen atmosphere for the photochemical upcycling of polystyrene [33]. In our studies, acetone was chosen as the solvent, since it provides better solubility for polystyrene, resulting in a more homogeneous reaction mixture and improved catalytic efficiency [55]. Using commercially available polystyrene as the model substrate, we first focused on evaluating a series of metal chlorides under comparable conditions, in order to identify previously unknown metal salts in the photochemical upcycling of PS (for details, see Table S1) [55]. Among the chlorides tested, FeCl_3_ and VCl_3_ afforded the most promising outcomes, delivering yields of up to 16% and 12%–18%, respectively. The superior performance of FeCl_3_ was consistent with literature precedent [33]. In contrast, the use of VCl_3_ resulted in modest product formation, reaching 12% yield after 18 h and a maximum of 18% after 48 h. However, the reactions were often accompanied by complex, nonclean mixtures and poor reproducibility, with no significant improvement upon extending the reaction time. These limitations, combined with the moderate efficiencies observed for other metal chlorides, such as MnCl_2_, BiCl_3_, or TiCl_3_, prompted us to shift our focus toward metal bromides as potential alternatives. Subsequent screening of various bromide salts revealed a distinct improvement in catalytic performance (for details, see Table S2) [55]. While most bromides, including MnBr_2_, NiBr_2_·dme, CoBr_2_, or ZnBr_2_, yielded only trace or minimal product formation (1%–2%), indium(III) bromide (InBr_3_) emerged as a notably effective catalyst, affording an 11% yield under standard conditions and up to 30% yield after 72 h. To our knowledge, indium photocatalysis is rather underdeveloped, while no literature precedent for plastics’ upcycling is known. Following the preliminary optimization of metal chloride and bromide catalysts, we directed our attention toward evaluating the effect of light irradiation on the photochemical upcycling of polystyrene (for details, see Table S3) [55]. A range of irradiation wavelengths was tested, revealing that the reaction efficiency strongly depended on the energy of the light used. Low‐energy sources, such as Compact Fluorescent Lamps (CFL), produced only trace yields, whereas UV‐violet irradiation significantly improved performance. Among the tested sources, 370 nm 2^nd^ generation Kessil LEDs delivered the best results, achieving yields of up to 31% after 72 h [55]. Therefore, these irradiation conditions were chosen as the optimal setup for all subsequent photochemical studies. Furthermore, conducting the reaction under air resulted in comparable, but lower yields; therefore, all reactions were carried out under an oxygen atmosphere (for details, see Table S3) [55]. Next, we investigated the effect of concentration on the photochemical aerobic upcycling of polystyrene (for details, see Table S4) [55]. By varying the acetone volume from 2 to 10 mL (corresponding to concentrations of 0.5 to 0.1 M) and analyzing the isolated products, we found that the optimal yield was obtained at approximately 0.2 M, yielding 22% of benzoic acid at 18 h. Regarding the reaction medium, different solvents, such as acetone, ethyl acetate (EtOAc), chloroform (CHCl_3_), dichloromethane (CH_2_Cl_2_), acetonitrile (ACN), dimethylformamide (DMF), dimethylsulfoxide (DMSO), or benzene were used under identical photochemical conditions (for details, see Table S5) [55]. Acetone and ethyl acetate provided the highest yields (22% and 23%, respectively), while other solvents resulted in low or trace product formation. Therefore, ethyl acetate was selected as the optimal solvent for further experiments. Moreover, combining acetone and ethyl acetate in a 1:1 ratio did not lead to any improvement in the reaction yield.

Then, we focused on evaluating the effect of additives and halogen sources on the photochemical aerobic upcycling of polystyrene (Table 1). Initially, we examined the influence of 2,2,2‐trichloroethanol as the additive. Interestingly, when the reaction was carried out in the absence of this alcohol, the yield was noticeably higher than when it was included, suggesting that it inhibits the reaction, likely by interfering with radical formation or quenching reactive intermediates (Table 1, entries 1 vs 2). Building on this observation, we proceeded to explore the role of different halogen sources and their loadings in the reaction. Several known chlorine and bromine donors, such as tetrabutylammonium chloride (TBACl), *p*‐toluenesulfonyl chloride (TsCl), tetrabutylammonium bromide (TBABr), *p*‐toluenesulfonyl bromide (TsBr), or *N*‐bromosuccinimide (NBS), were tested individually and in combination with one another to identify the most effective system (Table 1). When TBACl, TBABr, or TsCl were tested individually at a 10 mol% loading, they produced moderate yields of 30%, 17%, and 30%, respectively (Table 1, entries 2–4). In contrast, the use of TsBr or NBS resulted in higher yields of 39% (Table 1, entries 5 and 6). Increasing the halogen source loading, however, caused a significant drop in the yield to 4% in the first three cases. For TsBr and NBS, a slight improvement was observed upon increasing the loading, with yields rising only by 1% and 2%, respectively (for details, see Table S6) [55]. We then proceeded to test the previously used halogen donors in combinations to evaluate potential synergistic effects (Table 1, entries 7–14). However, most mixtures (e.g., TBABr/TsCl, TBABr/TsBr, TBABr/NBS, TBACl/TsCl, TBACl/TsBr, TBACl/NBS) afforded lower yields (14%–37%) (Table 1, entries 7–12), compared with the individual outcomes of TsBr or NBS. This suggests that combining these reagents did not significantly enhance the reaction outcome and, in some cases, may have led to competitive interactions that partially improved or even hindered the overall yield. However, when TsCl was combined with NBS, the yield increased to 43% (Table 1, entry 13), and further improvement to 51% was observed for the TsBr/NBS (1/1) system at 20 mol% total loading (Table 1, entry 14). Having established the optimal halogen donor system for our photochemical protocol, different loadings and ratios of the TsBr/NBS combination were studied. Reducing the total loading to 10 mol% led to a sharp decrease in yield (14%) (Table 1, entry 15), while increasing it to 30 mol% resulted in a yield of 26% (Table 1, entry 16), indicating that the reaction reaches its maximum efficiency at 20 mol% loading. Variation of the donor ratio to 3:1 or 1:3 produced yields between 36% and 39% (Table 1, entries 17 and 18), which were lower than those obtained with the 1:1 mixture, confirming that a balanced 1:1 ratio of TsBr and NBS at 20 mol% total loading represents the most effective condition identified in this study. Moreover, studies with different loadings of InBr_3_ revealed that a 10 mol% loading of the metal halide provided the highest efficiency, while both lower and higher concentrations resulted in decreased reaction yields (for details, see Table S7) [55]. Regarding the reaction time study, yields increased from 36% at 6 h to a maximum of 51% at 18 h, after which prolonged irradiation led to a decline (for details, see Table S8), most probably due to other secondary side reactions, including acid‐promoted transesterification of benzoic acid by ethyl acetate (the solvent) to form ethyl benzoate under the acidic conditions generated during irradiation [55]. Moreover, a standard polystyrene sample (MW = 390.000) was subjected to a 6 h photochemical reaction, affording a 42% yield of the isolated product, in order to evaluate the performance of shorter reaction times on pure PS substrates. Although high, the yields did not come close to the 51% obtained after 18 h of irradiation (for details, see Table S8) [55]. Finally, the use of two 370 nm 2^nd^ gen Kessil lamps under 6 h irradiation led to a decreased yield compared with the standard single‐lamp setup (for details, see Table S8) [55].

**TABLE 1:** Halogen source loading study for the photochemical upcycling of polystyrene.

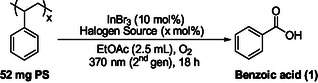			
Entry	Halogen source	Halogen source loading, mol%	Yield, %^a^
1^b^	TBACl	10	23
2	TBACl	10	30
3	TBABr	10	17
4	TsCl	10	30
5	TsBr	10	39
6	NBS	10	39
7	TBABr/TsCl (1/1)	10/10	33
8	TBABr/TsBr (1/1)	10/10	31
9	TBABr/NBS (1/1)	10/10	37
10	TBACl/TsCl (1/1)	10/10	20
11	TBACl/TsBr (1/1)	10/10	16
12	TBACl/NBS (1/1)	10/10	14
13	TsCl/NBS (1/1)	10/10	43
**14**	**TsBr/NBS (1/1)**	**10/10**	**51**
15	TsBr/NBS (1/1)	5/5	14
16	TsBr/NBS (1/1)	15/15	26
17	TsBr/NBS (3/1)	15/5	36
18	TsBr/NBS (1/3)	5/15	39

Reaction conditions: Polystyrene (PS) (52 mg, 0.50 mmol, 1.00 equiv.), InBr_3_ (18 mg, 10 mol%, 0.05 mmol, 0.10 equiv.) and halogen Source (10 mol%, 0.05 mmol, 0.10 equiv.) in EtOAc (2.5 mL), under LED (Kessil PR160L, 370 nm ‐ 2^nd^ gen) irradiation for 18 h at r.t. and oxygen atmosphere.

a Yield of isolated product (benzoic acid), after base‐acid wash and extractions.

b 2,2,2‐Trichloroethanol (9.6 μL, 20 mol%, 0.10 mmol, 0.20 equiv.) was used as an additive.

To evaluate the influence of reaction conditions on the upcycling of polystyrene, a series of control experiments were conducted (Table 2). Various setups were tested, including reactions performed under dark, under air or argon atmospheres, and with or without irradiation at specific wavelengths and temperatures (Table 2, entries 1 and 3–7). These experiments confirmed that both light and oxygen are crucial for the transformation. The reaction carried out under dark produced only traces of product (Table 2, entry 1). Additionally, when the reaction was conducted under sunlight, the reaction produced only 1% of product (Table 2, entry 2). The purely photochemical nature of the process—rather than a thermal one—was further verified by the absence of product formation at 45°C without light (Table 2, entry 3). When the reaction was performed at 45°C under 370 nm 2^nd^ gen irradiation, the yield dropped to 19% (Table 2, entry 4), likely due to inefficient light penetration through the oil bath. Furthermore, extended irradiation followed by thermal treatment with Cu(NO_3_)_2_ · 3H_2_O, following He and Zang's protocol [41], afforded a yield of up to 40%, which was still lower than our best result of 51% (Table 2, entry 5). The necessity of oxygen was confirmed by the formation of only 3% product under an argon atmosphere (Table 2, entry 7), whereas performing the reaction under air yielded 25%, highlighting the crucial role of pure oxygen atmosphere in the process (Table 2, entry 6). Finally, to further explore the behavior of mixed polymer systems, the standard reaction conditions were applied to PS in the presence of equimolar amounts of other common polymers, including high‐ and low‐density polyethylene (HDPE and LDPE), polypropylene (PP), or polyethylene terephthalate (PET) (Table 2, entries 8–11). The reaction proceeded efficiently, demonstrating the system's tolerance toward mixed plastic waste and selectivity toward polystyrene's degradation. The slight decrease in yield observed suggests that the photocatalytic system may also interact, to a minor extent, with these additional polymers.

**TABLE 2:** Control experiments for the photochemical upcycling of polystyrene.

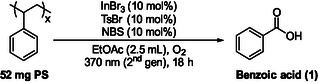		
Entry	Control variations	Yield, %^a^
1	Under dark	traces
2	Under sunlight	1
3	No hv, at 45°C	0
4	370 nm (2^nd^ gen) irradiation, at 45°C	19
5^b^	18 h irradiation and 24 h without irradiation at 140°C after addition of Cu(NO_3_)_2_•3H_2_O (20 mol%)	40
6	Under air	25
7	Under argon	3
8	In the presence of 14 mg of a HD‐PE plastic bottle	35
9	In the presence of 14 mg of a LD‐PE plastic bag	34
10	In the presence of 21 mg of a PP plastic cup	38
11	In the presence of 96 mg of a PET water bottle	39

Reaction conditions: Polystyrene (PS) (52 mg, 0.50 mmol, 1.00 equiv.), InBr_3_ (18 mg, 10 mol%, 0.05 mmol, 0.10 equiv.), TsBr (12 mg, 10 mol%, 0.05 mmol, 0.10 equiv.) and NBS (9 mg, 10 mol%, 0.05 mmol, 0.10 equiv.) in EtOAc (2.5 mL), under LED (Kessil PR160L, 370 nm ‐ 2^nd^ gen) irradiation for 18 h at r.t. and oxygen atmosphere.

a Yield of isolated product (benzoic acid), after base‐acid wash and extractions.

b After 18 h of irradiation, Cu(NO_3_)_2_•3H_2_O (20 mol%, 24 mg) in a mixture of EtOAc:MeCN (2:1.5 mL) was added and the reaction mixture was left under stirring at 140°C for 24 h.

**SCHEME 2:**
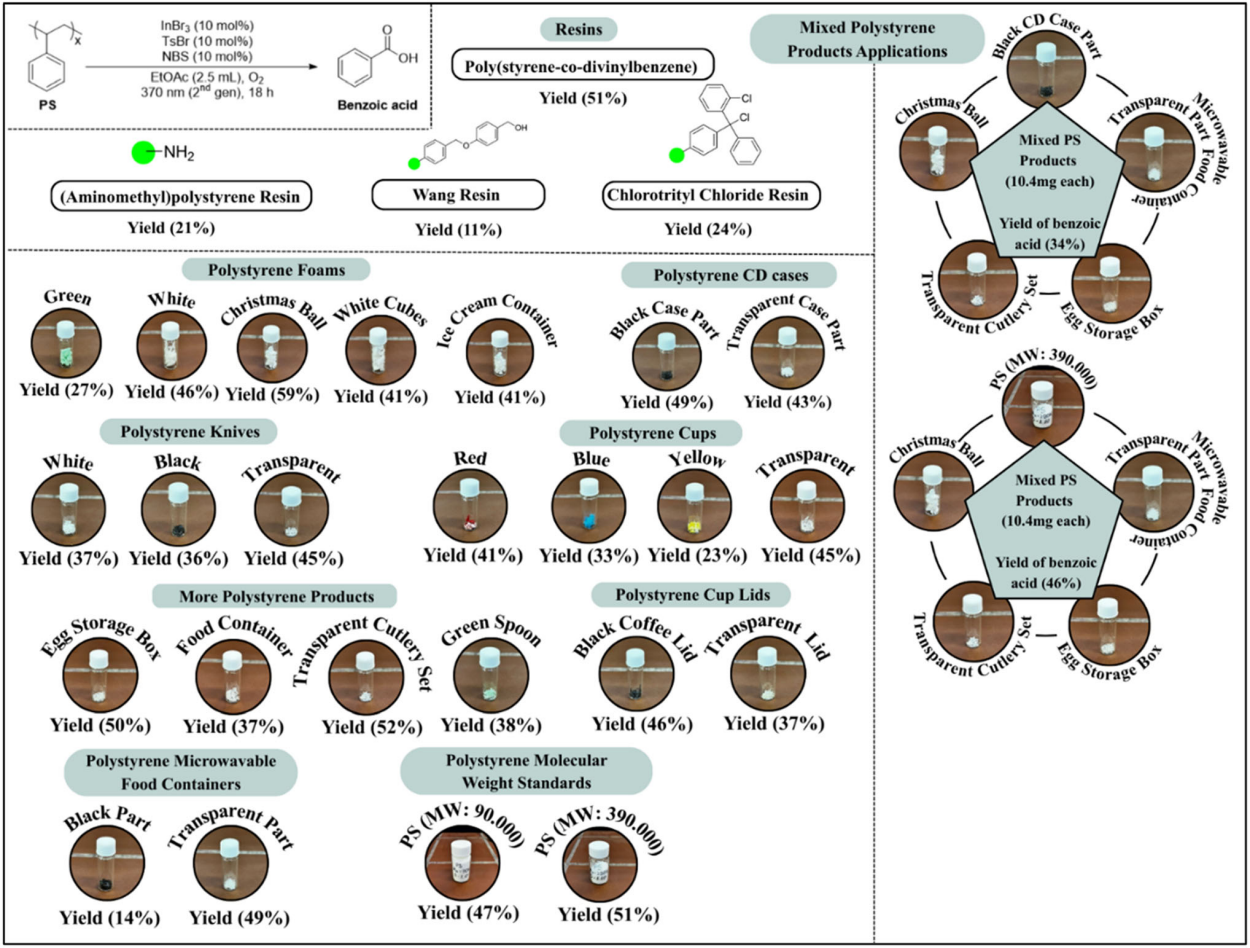
Real‐life applications of the developed photochemical upcycling method to commercial and mixed polystyrene sources.

Having identified the optimal reaction conditions, we envisaged the use of a variety of everyday polystyrene‐based products, as well as several commercial resins, as the starting materials (Scheme 2). Poly(styrene‐co‐divinylbenzene) served as the model substrate throughout this study, while the examined materials span across different physical forms, degrees of cross‐linking, and functionalization. In this context, the resins investigated—namely aminomethyl polystyrene, Wang resin, and chlorotrityl chloride resin—are well‐established polystyrene‐based supports, commonly used in synthetic chemistry, while the cross‐linked poly(styrene‐co‐divinylbenzene) material represents a typical polystyrene architecture employed alongside these substrates. Application of the optimized conditions to these materials consistently resulted in conversion of the polystyrene backbone into benzoic acid as the major product, with yields depending on polymer composition, functional linker density, and material morphology. For the commercial resins, benzoic acid was obtained in yields ranging from 11% to 24%, whereas consumer polystyrene products afforded yields between approximately 14% and 59%, underscoring the robustness of the method across structurally and physically diverse PS sources. The reported yields correspond to isolated benzoic acid obtained after acid–base washing and extraction and were calculated under the assumption that each tested consumer item consisted solely of polystyrene, while no pretreatment was performed. Indeed, the lower yields obtained for the commercial resins could be attributed to a major degree to the fact that are not consisting entirely from polystyrene. Among colored PS cups, the red and blue materials afforded moderate yields (41% and 33%, respectively), likely reflecting pigment‐induced attenuation of light penetration, while thicker molded items, such as white and black PS knives, yielded similar conversions (37% and 36%), suggesting only a minor color influence in these cases; the green PS spoon (38%) performed comparably. CD case components exhibited relatively high reactivity, affording 49% for the black part and 43% for the transparent part. In contrast, green PS foam delivered a lower yield (27%), whereas an ice‐cream foam container performed better (41%), highlighting variability among commercial foam formulations. The yellow PS cup (23%) gave one of the lowest yields, consistent with the general trend that colored plastics often perform less efficiently, due to pigment interference with photo‐oxidation, whereas transparent PS products—including a transparent knife (45%), a small transparent cup (45%), and a transparent cutlery set (52%)—consistently afforded higher yields owing to improved light transmission. Similarly, white PS foam (46%), white foam cubes (41%), and a black coffee cup lid (46%) performed efficiently. The transparent frozen‐drink cup lid also afforded a good yield (37%), while a pronounced contrast was observed for the microwavable food container components (14% for the black part versus 49% for the transparent part), likely reflecting differences in copolymer composition and heat‐resistant additives. The egg storage box (50%) and food container (37%) showed moderate to high conversions, consistent with typical PS formulations, and notably, the foam Christmas ball afforded the highest yield among all consumer products examined (59%). In addition, PS standards with molecular weights of 90.000 and 390.000 yielded 47% and 51% benzoic acid, respectively, in close agreement with the results obtained for the other polystyrene substrates. Finally, to assess performance under more realistic waste‐stream conditions, a mixture of different PS materials that had previously shown high individual yields—including the foam Christmas ball, transparent cutlery set, egg storage box, transparent part of the microwavable food container, and the black part of a CD case—was subjected to the optimized conditions, affording benzoic acid in 34% yield (Scheme 2); replacement of the black CD case component with the PS standard (MW 390.000) increased the yield to 46%, further demonstrating the effectiveness of the protocol in heterogeneous mixed‐polystyrene systems.

**SCHEME 3:**
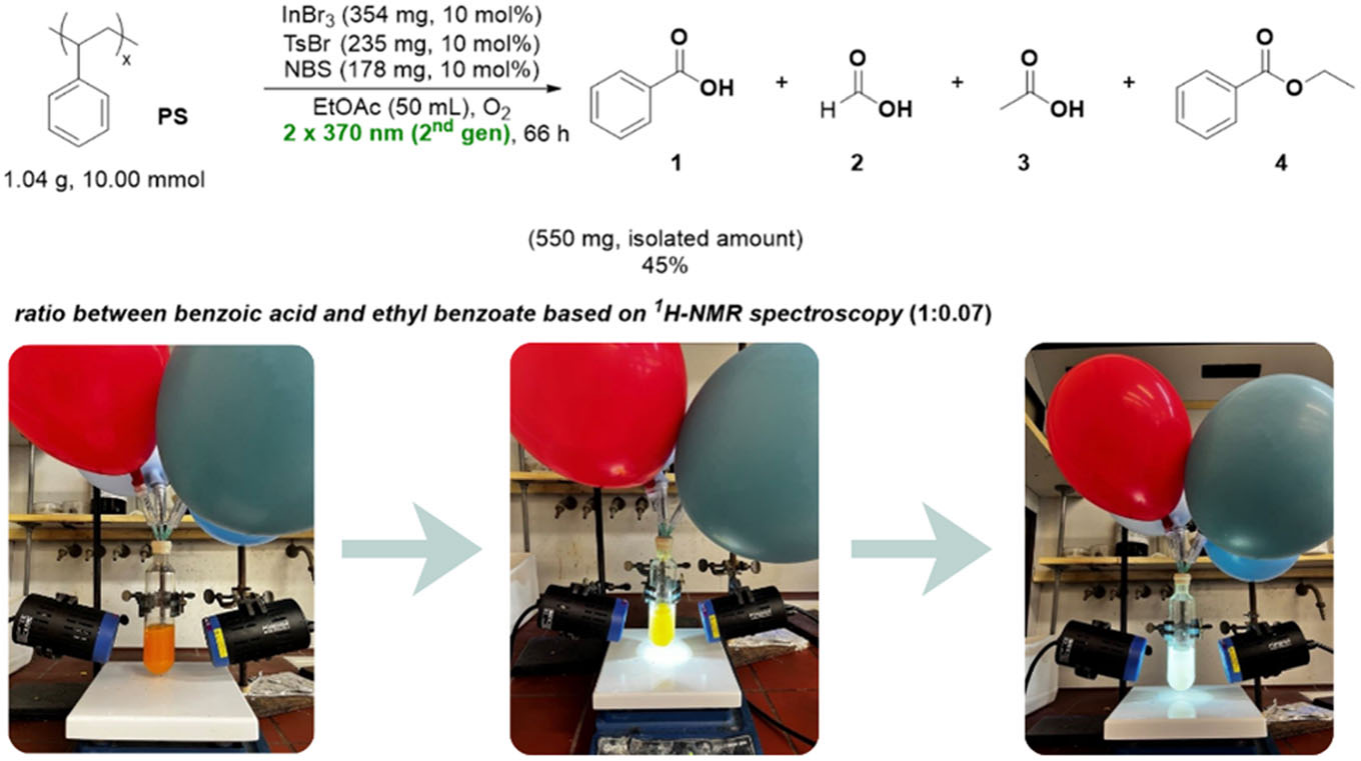
Large‐scale reaction and visual overview of the photochemical upcycling of polystyrene.

The developed protocol was further validated on a larger scale, using 1 g of polystyrene as the starting material (Scheme 3). In this experiment, two Kessil 370 nm (second generation) lamps were employed as the light source (Scheme 3). The reaction progress was carefully monitored over a period of 72 h, with attention to the consumption of PS. Despite the scale‐up, the process maintained a high level of efficiency, affording benzoic acid in 45% yield. Analysis of the crude reaction mixture by ^1^H‐NMR spectroscopy revealed additional minor components, providing insight into the reaction mechanism. Signals corresponding to ethyl benzoate were detected, suggesting partial ester formation, most likely arising from acid‐promoted transesterification of benzoic acid by ethyl acetate (the solvent) under the acidic conditions generated during irradiation, with acetic acid formed from oxidative degradation of ethyl acetate, contributing to the acidic environment. In addition, the formation of formic acid and acetic acid supports the occurrence of oxidative degradation pathways.

To gain insight into the reaction mechanism, a series of mechanistic experiments were performed by selectively omitting components of the catalytic system and by introducing radical and singlet‐oxygen quenchers (Table 3). When PS was subjected to irradiation in the presence of the solvent only, a negligible 3% yield was obtained, confirming that light irradiation alone is insufficient to break down PS (Table 3, entry 1). This observation underscores the necessity of the photocatalytic system, as the polymer's inert nature and strong C–C backbone require an activated pathway for effective oxidative transformation. When the reaction was performed using only InBr_3_, a moderate yield of 19% was achieved, indicating that the metal halide can partially promote the oxidative process‐likely through light‐induced generation of reactive bromine or indium species that facilitate C–H activation and chain scission (Table 3, entry 2). In contrast, reactions using only NBS or TsBr afforded lower yields of 6% and 10%, respectively, suggesting that while these halogen sources can undergo limited homolytic cleavage under photochemical conditions to generate bromine radicals, their activity alone is insufficient for efficient polymer oxidation (Table 3, entries 3 and 4). The highest yield (51%) was obtained when both InBr_3_ and the brominating agents were combined, demonstrating a clear synergistic effect between the metal center and the halogen source. The involvement of reactive oxygen species was probed using quenching experiments (Table 3, entries 8–11). Hydrogen peroxide alone (10 equiv.) yielded only 1% product (Table 3, entry 8), while its addition under standard conditions still reduced the yield to 19% (Table 3, entry 9), indicating its quenching effect. Moreover, radical trapping experiments using singlet oxygen quenchers (NaN_3_ and DABCO) nearly suppressed the reaction, producing only 1% and trace amounts of product, respectively (Table 3, entries 10 and 11).

**TABLE 3:** Mechanistic experiments for the photochemical upcycling of polystyrene.

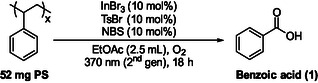		
Entry	Quenchers: mechanistic variations	Yield, %^a^
1	EtOAc [no InBr_3_, TsBr, NBS]	3
2	InBr_3_ [no TsBr, NBS]	19
3	NBS [no InBr_3_, TsBr]	6
4	TsBr [no InBr_3_, NBS]	10
5	Br_2_ [no InBr_3_, TsBr, NBS]	6
6	Br_2_ and InBr_3_ [no TsBr, NBS]	35
7	Br_2_, InBr_3_ and TsBr [no NBS]	50
8	Hydrogen peroxide (30 wt%) solution (10 equiv.) [no InBr_3_, TsBr, NBS]	1
9	Addition of hydrogen peroxide (30 wt%) solution (10 equiv.)	19
10	Addition of NaN_3_ (0.50 mmol)	1
11	Addition of DABCO (0.50 mmol)	traces

Reaction conditions: Polystyrene (PS) (52 mg, 0.50 mmol, 1.00 equiv.), InBr_3_ (18 mg, 10 mol%, 0.05 mmol, 0.10 equiv.), TsBr (12 mg, 10 mol%, 0.05 mmol, 0.10 equiv.) and NBS (9 mg, 10 mol%, 0.05 mmol, 0.10 equiv.) in EtOAc (2.5 mL), under LED (Kessil PR160L, 370 nm ‐ 2^nd^ gen) irradiation for 18 h at r.t. and oxygen atmosphere.

a Yield of isolated product (benzoic acid), after base‐acid wash and extractions.

**FIGURE 1:**
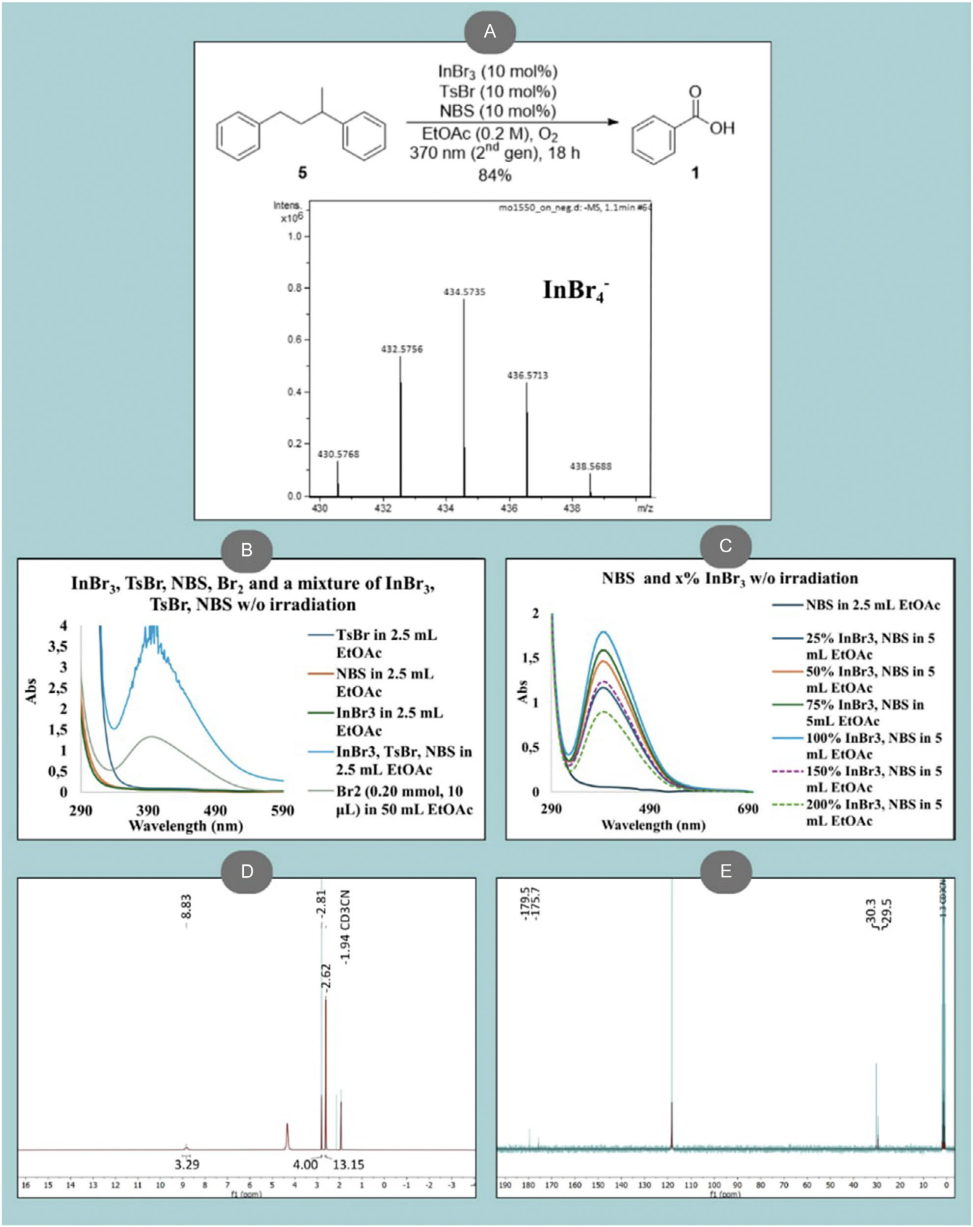
(A) HRMS spectra of the InBr_4_^−^ formation after 18 h of reaction time; (B) UV‐Vis spectra of InBr_3_ (0.05 mmol), TsBr (0.05 mmol), NBS (0.05 mmol), a mixture of InBr_3_, TsBr, NBS in 2.5 mL EtOAc, and Br_2_ (0.20 mmol, 10 μL) in 50 mL EtOAc; (C) UV‐Vis spectra of NBS (0.05 mmol) and InBr_3_ (0.0125 mmol, 0.0250 mmol, 0.0375 mmol, 0.0500 mmol, 0.0750 mmol, 0.100 mmol) mixture in proportional ratios in 2.5 mL EtOAc; (D) ^1^H NMR spectrum (400 MHz, acetonitrile‐*d*_3_) of NBS (0.1M) (cyan) and NBS (0.1M) ‐ InBr_3_ (0.1M) (red) in CD_3_CN, by the integration we observed a ratio of NBS:succinimide 1:3.29; and (E) ^13^C NMR spectrum (100 MHz, acetonitrile‐*d*_3_) of NBS (0.1M) (red) and NBS (0.1M) ‐ InBr_3_ (0.1M) (cyan) in CD_3_CN.

To obtain additional insight into our photochemical upcycling protocol, we employed model substrate **5**, which can be regarded as a styrene dimer (Figure 1). When subjected to the developed aerobic photochemical reaction conditions, **5** delivered benzoic acid in high yield (84%) after 18 h, indicating that our protocol is effective for both benzylic and tertiary carbon oxidation. Substrate **5** was also treated under the reaction conditions, which was analyzed using direct infusion high‐resolution mass spectrometry (DI‐HRMS). This technique provides several advantages including the elimination of time‐consuming chromatographic separation steps, a reduced risk of solvent‐induced degradation of sensitive analytes and the capability to perform comprehensive mass spectrometric profiling of all species within a defined scan range [56, 57, 58, 59]. The aerobic photochemical upcycling reaction was carried out both in the presence and absence of the radical scavenger TEMPO. The analysis revealed masses that could be assigned to adducts, corresponding to intermediates containing hydroxy or peroxy groups (see pages S40–S73 of the Supporting Information) [55]. Additionally, peaks associated with various benzylic oxygenated and non‐oxygenated compounds—previously identified in our group's earlier studies [45, 47, 48]—were observed, along with radical intermediates trapped by the scavenger [55]. Furthermore, an [InBr_4_]^−^ complex was independently synthesized to verify its accessibility under the reaction conditions [55]. Since tosyl bromide or *N*‐bromosuccinimide (NBS) cannot directly furnish bromide anions without photochemical activation and radical pathways, tetrabutylammonium bromide (TBABr) was employed as the external bromide source, supplying both the halide and the stabilizing counterion. The resulting complex was characterized by high‐resolution mass spectrometry (HRMS) to confirm its formation and evaluate its stability, thereby supporting its relevance to the proposed reaction mechanism (see page S74 of the Supporting Information) [55]. The [InBr_4_]^−^ species was clearly detected in the reaction mixture, exhibiting a high‐intensity peak that remained consistent across all experimental stages (Figure 1, A). The independently synthesized [NBu_4_]^+^[InBr_4_]^−^ complex was subsequently employed in the photochemical reaction, as the sole photochemical promoter, leading to a 22% yield of benzoic acid (see page S75 of the Supporting Information), which is similar to InBr_3_ alone (see Table 3, entry 2, 19% yield of benzoic acid), or in combination with TsBr or NBS individually (see page S75 of the Supporting Information, 34% and 34% yield of benzoic acid, respectively) [55], which is similar to the InBr_3_/TBABr/TsBr and InBr_3_/TBABr/NBS systems (see Table 1, entries 8 and 9, 31% and 37% yield of benzoic acid, respectively). To determine whether tosyl bromide or *N*‐bromosuccinimide (NBS) act through the same pathway to produce the [InBr_4_]^−^ species photochemically, each reagent was tested individually under identical conditions. In both cases, formation of the [InBr_4_]^−^ complex was confirmed, demonstrating that both brominating agents are capable of generating the same indium–bromide species independently (see page S77 of the Supporting Information) [55].

To elucidate the photochemical behavior and possible interactions between the bromine sources and the indium catalyst, UV–Vis absorption spectra were recorded for the individual components and their mixtures in ethyl acetate (EtOAc), both before and after irradiation (see page S78‐S85 of the Supporting Information) [55]. Solutions of InBr_3_ (0.05 mmol), TsBr (0.05 mmol), and NBS (0.05 mmol) were each prepared in 2.5 mL EtOAc, followed by analysis of their mixtures to obtain a comprehensive understanding of the system (Figure 1,B). Upon combining the components, a pronounced spectral evolution was observed, characterized by the emergence of an intense absorption band around 400 nm, suggesting the formation of a new species. Based on these observations, we proposed that molecular bromine (Br_2_) could be generated under the reaction conditions. To test this hypothesis, a bromine reference solution was prepared by diluting 10 μL Br_2_ (0.20 mmol) in 50 mL EtOAc to obtain a suitable concentration for comparison. The resulting UV–Vis spectrum displayed a band at a similar wavelength, thereby supporting the formation of Br_2_ in the reaction mixture (Figure 1, B). To evaluate the ability of molecular bromine to promote the photochemical degradation of polystyrene, the reaction was carried out in the presence of Br_2_ alone (Table 3, entry 5). A similar extremely low reactivity trend (6% yield) was observed as with NBS used independently (Table 3, entry 3). When Br_2_ was combined with InBr_3_ (Table 3, entry 6), the reaction proceeded in 35% yield—comparable to that obtained with the NBS–InBr_3_ system (Table 1, entry 6). Moreover, the additional introduction of TsBr to the Br_2_–InBr_3_ couple further enhanced the reaction, affording a 50% yield (Table 3, entry 7), which is nearly identical to the optimal 51% yield achieved under the best conditions (Table 1, entry 14).

Subsequently, we investigated the absorption behavior of each component individually and in binary combinations to determine the origin of the observed spectral changes (see pages S78‐S85 of the Supporting Information) [55]. The comparative UV–Vis analysis revealed that the interaction responsible for the new absorption band originates primarily from the InBr_3_–NBS pair. This observation suggests the formation of a ground‐state charge–transfer (CT)/donor–acceptor adduct or a Lewis‐acid–activated NBS complex. Such complexes are known and account for the generation of new absorption bands and their ability to generate radical species upon irradiation [60]. Furthermore, literature precedents have shown that photoinert metal salts can form transient CT adducts with *N*‐halosuccinimides (NXS), rendering their combination photoactive: For example, CeCl_3_·NXS transient CT complexes were reported to absorb visible light and to transfer an electron to NXS, initiating halogen radical chemistry [61, 62, 63]. Such metal–NXS CT behavior provides a close mechanistic analogy to how a Lewis‐acidic indium halide might activate NBS under light [61, 62, 63]. Besides, *N*‐bromosuccinimide (NBS) is a well‐known source of bromine and/or bromine radicals, particularly under Lewis‐acidic or photochemical conditions [61, 62, 63]. Given that indium(III) halides, such as InBr_3_, act as strong Lewis acids and readily interact with halogen donors, their combination with NBS is chemically reasonable and likely promotes in situ formation of reactive bromine species. Their interaction was further examined by maintaining NBS at 0.05 mmol and gradually increasing the amount of InBr_3_ (25%, 50%, 75%, 100%, 150%, and 200% relative to NBS) (Figure 1C). The absorption intensity increased with higher InBr_3_ concentration, reaching a maximum at a 1:1 InBr_3_–NBS ratio, indicating the formation of a distinct stoichiometric complex between the two species. UV‐Vis studies after 10 min of light irradiation showed only slight changes in the absorption bands for most compounds and their mixtures, except from NBS‐InBr_3_ and NBS‐TsBr‐InBr_3_, where the prominent absorption band around 400 nm disappeared. Irradiation of bromine also caused changes in its absorption band, whereas the UV‐Vis spectrum of succinimide, a possible product formed after bromine removal from NBS, showed no significant absorption [55].

We continued our investigation by conducting ^1^H‐NMR analysis of the photochemical system to study the effect of InBr_3_ addition in an *N*‐bromosuccinimide (NBS, 0.1 M) solution in CD_3_CN (see pages S86–S91 of the Supporting Information) [55]. Incremental additions of InBr_3_ were performed at 25%, 50%, 75%, and 100% relative to NBS. As the concentration of InBr_3_ increased, we observed a gradual decrease in the characteristic NBS signal at 2.80 ppm, accompanied by the emergence of a new resonance at 2.61 ppm, which intensified progressively with higher InBr_3_ loading. Based on our prior conclusions regarding bromine and succinimide formation, we also recorded the ^1^H‐NMR spectrum of pure succinimide for comparison. The newly formed signal in the reaction mixture matched perfectly with that of succinimide, confirming its formation. Quantitatively, the NBS:succinimide ratio evolved from approximately 1:0.99 at 25% InBr_3_ to 1:1.74, 1:2.60, and finally 1:3.29 at 100% InBr_3_ (Figure 1D), demonstrating a steady conversion of NBS to succinimide. This observation was further verified by ^13^C‐NMR spectroscopy, which revealed distinct carbon resonances corresponding to succinimide, providing additional evidence for its generation in the system (Figure 1E). The observed spectral changes can be rationalized by considering the Lewis acidic nature of indium(III) bromide (InBr_3_). As a strong Lewis acid, InBr_3_ may coordinate to NBS (either through its carbonyl oxygen atoms or the *N*–Br bond) [63, 64], thereby facilitating heterolytic cleavage of the N–Br linkage and promoting the generation of bromine‐based species. Similar interactions between *N*‐halosuccinimides and other metal‐based Lewis acids have also been reported in the literature [61, 62, 65, 66, 67, 68]. This interaction effectively lowers the activation barrier for NBS decomposition and accelerates the formation of succinimide. Consequently, the progressive decrease of NBS resonances and the concomitant increase in succinimide signals with higher InBr_3_ concentrations suggest that the addition of InBr_3_ shifts the equilibrium and enhances the rate of NBS conversion.

**SCHEME 4:**
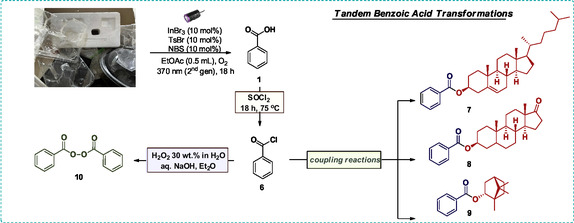
Further functionalization reactions using obtained polystyrene as starting material.

In the last stage of our investigation, we explored the synthetic versatility of the photochemically upcycled polystyrene product after extractions, benzoic acid, that was transformed into a series of compounds of industrial and pharmaceutical interest (Scheme 4). Initially, benzoic acid was converted to benzoyl chloride (**6**) using thionyl chloride. The resulting benzoyl chloride served directly as a key intermediate for further derivatization. Subsequent esterification reactions of benzoyl chloride with three structurally diverse alcohols—cholesterol, *epi*‐androsterone, or borneol—afforded a series of novel benzoyl esters (**7–9**). These compounds present potential biological and pharmacological significance, since benzoyl esters of steroids and terpenoids has been shown to increase lipophilicity and often modulate biological activity—for example, enhancing antimicrobial, anti‐inflammatory, or other pharmacological properties [69, 70]. In addition, benzoyl chloride was subjected to further oxidation to generate benzoyl peroxide (**10**), a compound of considerable industrial value as an oxidant and active ingredient in dermatological formulations for the treatment of acne and skin disorders [71, 72].

**SCHEME 5:**
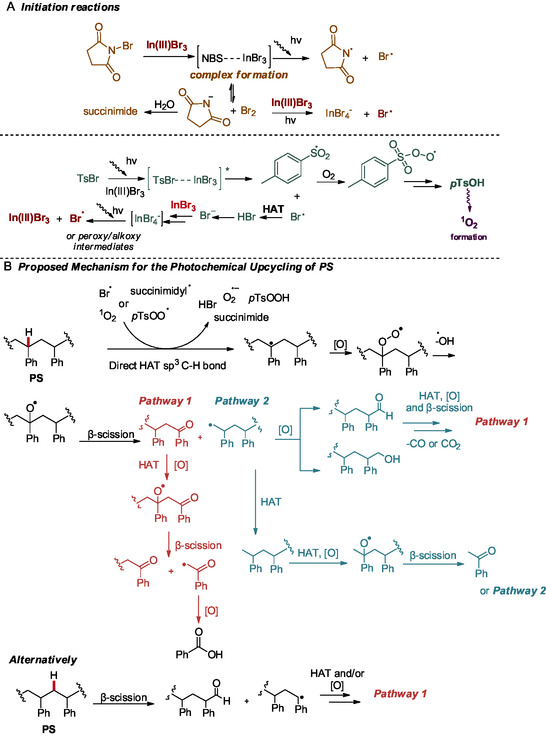
Proposed mechanism for the photochemical upcycling of plastics using InBr, TsBr, and NBS.

Having all the abovementioned data in hand, we were able to propose possible mechanistic pathways for the photochemical upcycling protocol (Scheme 5). NBS, upon irradiation, promotes poorly the upcycling process (6%, Table 3, entry 3). However, NBS forms a complex with InBr_3_, which weakens the N–Br bond and sets the stage for radical generation (Scheme 5A, top). Besides, it is known that strong Bronsted or Lewis acids can coordinate onto the O atom of the succinimide, which draws the electron density away from the halogen, thus amplifying the electrophilicity of the halogen by increasing the N−X bond polarization [61, 62, 63]. Upon irradiation, the complex undergoes homolytic cleavage to produce bromine radicals (Br•) and succinimidyl radicals, initiating a radical chain reaction: Br• abstracts hydrogen from the substrate to form a carbon radical. In line with literature reports [73], coordination of NBS to InBr_3_ can also enable nucleophilic attack of a bromide ligand from InBr_3_ onto the electrophilic bromine of NBS, leading to the formation of small amounts of Br_2_, in equilibrium along with succinimide (or succinimide anion, which can be protonated in the presence of trace water). This Br_2_ reacts with InBr_3_, producing detectable InBr_4_^−^ and bromine radical. Additionally, in the absence of light, the same NBS‐InBr_3_ complex can generate Br_2_ and succinimide via heterolytic cleavage, which under irradiation contributes further to radical formation. This in total is the major pathway for the upcycling process. Furthermore, upon irradiation, tosyl bromide undergoes homolytic cleavage to generate a tosyl radical and a bromine radical. This cleavage is relatively inefficient, when tosyl bromide acts alone (10%, Table 3, entry 4), resulting in low yields of benzoic acid. However, in the presence of InBr_3_, the S–Br bond is weakened, facilitating the formation of a bromine radical (Scheme 5A, bottom). The bromine radical can then abstract a hydrogen atom to produce HBr, which subsequently dissociates to form a bromide anion (Br^−^). Once formed, the bromide coordinates with InBr_3_ to yield the tetrahalide complex InBr_4_^−^, whose formation has been reported, once in bromine‐rich environments [74]. This complex, confirmed by HRMS analysis in our protocol, can also serve as a source of bromine radicals, while regenerating InBr_3_ under photochemical conditions. Additionally, the presence of oxygen facilitates the efficient trapping of the tosyl radical (Ts^•^) [75], which subsequently converts into *p*TsOH, an already reported acidic catalyst for the photochemical upcycling of PS via singlet oxygen generation [43]. Reaction of the tosyl radical with oxygen yields a peroxy intermediate, as confirmed by HRMS both through TEMPO‐trapped radical adducts and after hydrogen atom abstraction processes occurring on the polymer matrix. Based on the above findings, oxidative degradation of the polystyrene matrix occurs to a measurable extent, even when InBr_3_ is used as the sole catalyst (19%, Table 3, entry 2). In order to account for this reactivity, under irradiation, InBr_3_ can act as a photoactive Lewis acid via ligand‐to‐metal charge transfer (LMCT) excitation. In an LMCT process, a halide ligand donates an electron to the empty orbitals of the metal center upon photon absorption, transiently generating a metal‐reduced/halogen‐oxidized excited state [76]. This charge–transfer excitation weakens the In–Br bond and can relax through homolytic cleavage, leading to bromine radical (Br·) release and forms a reduced species, which can subsequently be reoxidized to In(III)Br_3_. Similar LMCT‐driven halogen radical formation can be found for iron or copper‐based halides, supporting direct Cl· or Br· radical formation without any external photocatalyst [77]. Supporting evidence from Dilmans’ work on indium‐mediated photoredox transformations demonstrates that indium halides can participate in single‐electron processes under light [78]. The resulting Br· radicals abstract benzylic hydrogens from the polymer, leading to chain oxidation and cleavage, explaining the benzoic acid yield in Table 3, entry 2.

After initiation, bromine and succinimidyl radicals drive the photochemical upcycling of polystyrene (PS) through two main pathways: hydrogen atom transfer (HAT) and singlet oxygen oxidation (Scheme 5B). In the HAT pathway, abstraction of 3° or 2° C–H bonds generates polymer radicals that undergo oxidation and *β*‐chain scission, yielding aromatic ketones and alkyl radicals. Subsequent oxidation, decarbonylation, or decarboxylation converts these intermediates into benzoic acid and related small molecules. Upon irradiation, polystyrene can also undergo degradation via a singlet oxygen‐mediated pathway, initiated by the excited catalyst. Singlet oxygen either inserts at benzylic sites or abstracts hydrogen, forming peroxy intermediates that decompose into reactive oxygen species (ROS) such as hydroxyl and superoxide radicals. These species promote *β*‐chain scission and oxidation along the polymer backbone, ultimately generating the same key intermediates observed in Pathways 1 and 2.

## 3 Conclusion

In summary, this study establishes a new approach for the photochemical upcycling of polystyrene through a bromine‐radical–mediated, indium(III) bromide–catalyzed pathway. The developed catalytic system introduces, for the first time, indium salts as efficient mediators for polymer deconstruction, enabling the depolymerization of polystyrene under mild conditions. This method exhibits broad substrate compatibility and selectivity, as well as excellent scalability, achieving the sustainable conversion of post‐consumer polystyrene waste into benzoic acid. Comprehensive mechanistic investigations using HRMS, UV–Vis, and NMR techniques reveal the key steps of the transformation giving emphasis to the synergistic interaction between bromine radicals and indium catalysis in promoting C—C bond cleavage. The identification of reactive intermediates, including bromine‐derived species and indium–bromide complexes, provides insight into the mechanistic pathways and validates the cooperative synergy between the halogen donor and the Lewis acid catalyst. Overall, this work introduces a new protocol for polystyrene upcycling, contributing significantly to the broader pursuit of circularity in polymer chemistry.

## Supporting Information

Additional supporting information can be found online in the Supporting Information section. The authors have cited additional references within the Supporting Information [78, 79, 80, 81, 82, 83, 84, 85, 86, 87, 88].

## Funding

This study was supported by the Hellenic Foundation for Research and Innovation (Grant 15949)

## Conflicts of Interest

The authors declare no conflicts of interest.

## Supporting information

Supplementary Material

## Data Availability

The data that support the findings of this study are available in the supplementary material of this article.

## References

[1] R. Geyer ,” A Brief History of Plastics,” In Mare Plasticum‐the Plastic Sea, eds. M. Streit‐Bianchi , M. Cimadevila , W. Trettnak , (Springer, 2020), 31–47.

[2] R. Geyer ,” Production, Use, and Fate of Synthetic Polymers,” In Plastic Waste and Recycling, ed. T. M. Letcher , (Academic Press, 2020), 13–32.

[3] S. Chu , B. Zhang , X. Zhao , et al., “Photocatalytic Conversion of Plastic Waste: From Photodegradation to Photosynthesis,” Advanced Energy Materials 12 (2022): 2200435.

[4] R. Geyer , J. R. Jambeck , and K. L. Law , “Production, Use, and Fate of All Plastics Ever Made,” Science Advances 3 (2017): e1700782. 28776036 10.1126/sciadv.1700782 PMC5517107

[5] I. E. Napper and R. C. Thompson , “Plastics and the Environment,” Annual Review of Environment and Resources 48 (2023): 55–79.

[6] Y. Chen , A. K. Awasthi , F. Wei , Q. Tan , and J. Li , “Single‐use Plastics: Production, Usage, Disposal, and Adverse Impacts,” The Science of the Total Environment 752 (2021): 141772. 32892042 10.1016/j.scitotenv.2020.141772

[7] D. E. Macarthur , “Beyond Plastic Waste,” Science 358 (2017): 843. 29146782 10.1126/science.aao6749

[8] X. Chen , Y. Wang , and L. Zhang , “Recent Progress in the Chemical Upcycling of Plastic Wastes,” ChemSusChem 14 (2021): 4137–4151. 34003585 10.1002/cssc.202100868

[9] B. Jiang , A. E. Kauffman , L. Li , et al., “Health Impacts of Environmental Contamination of Micro‐ and Nanoplastics: A Review,” Environmental Health and Preventive Medicine 25 (2020): 29. 32664857 10.1186/s12199-020-00870-9 PMC7362455

[10] X. Zhao , B. Boruah , K. F. Chin , M. Đokić , J. M. Modak , and H. S. Soo , “Upcycling to Sustainably Reuse Plastics,” Advanced Materials 34 (2022): 2100843. 10.1002/adma.202100843 34240472

[11] J. Zheng and S. Suh , “Strategies to Reduce the Global Carbon Footprint of Plastics,” Nature Climate Change 9 (2019): 374–378.

[12] A. S. Pottinger , R. Geyer , N. Biyani , et al., “Pathways to Reduce Global Plastic Waste Mismanagement and Greenhouse Gas Emissions by 2050,” Science 386 (2024): 1168–1173. 39541435 10.1126/science.adr3837

[13] N. Chaukura , W. Gwenzi , T. Bunhu , D. T. Ruziwa , and I. Pumure , “Potential Uses and Value‐Added Products Derived from Waste Polystyrene in Developing Countries: A Review,” Resources, Conservation, and Recycling 107 (2016): 157–165.

[14] Z. Xu , D. Sun , J. Xu , R. Yang , J. D. Russell , and G. Liu , “Progress and Challenges in Polystyrene Recycling and Upcycling,” ChemSusChem 17 (2024): e202400474. 38757556 10.1002/cssc.202400474

[15] W. Li , W. Zhao , H. Zhu , Z.‐J. Li , and W. Wang , “State of the Art in the Photochemical Degradation of (Micro)plastics: From Fundamental Principles to Catalysts and Applications,” Journal of Materials Chemistry A 11 (2023): 2503–2527.

[16] O. G. Mountanea , E. Skolia , and C. G. Kokotos , “Photochemical Upcycling and Recycling of Plastics: Achievements and Future Opportunities,” Green Chemistry 26 (2024): 8528–8549.

[17] M. R. Karimi Estahbanati , X. Y. Kong , A. Eslami , and H. S. Soo , “Current Developments in the Chemical Upcycling of Waste Plastics Using Alternative Energy Sources,” ChemSusChem 14 (2021): 4152–4166. 34048150 10.1002/cssc.202100874

[18] E. Skolia , O. G. Mountanea , and C. G. Kokotos , “Photochemical Upcycling of Polystyrene Plastics,” Trends in Chemistry 5 (2023): 116–120.

[19] D. Ravelli , M. Fagnoni , and A. Albini ,” Photoorganocatalysis. What for?,” Chemical Society Reviews 42 (2013): 97–113. 22990664 10.1039/c2cs35250h

[20] N. A. Romero and D. A. Nicewicz , “Organic Photoredox Catalysis,” Chemical Reviews 116 (2016): 10075–10166. 27285582 10.1021/acs.chemrev.6b00057

[21] A. Y. Chan , I. B. Perry , N. B. Bissonnette , et al., “Metallaphotoredox: The Merger of Photoredox and Transition Metal Catalysis,” Chemical Reviews 122 (2022): 1485–1542. 34793128 10.1021/acs.chemrev.1c00383 PMC12232520

[22] L. Capaldo , D. Ravelli , and M. Fagnoni , “Direct Photocatalyzed Hydrogen Atom Transfer (HAT) for Aliphatic C–H Bonds Elaboration,” Chemical Reviews 122 (2022): 1875–1924. 34355884 10.1021/acs.chemrev.1c00263 PMC8796199

[23] L. Capaldo and D. Ravelli , “Hydrogen Atom Transfer (HAT): A Versatile Strategy for Substrate Activation in Photocatalyzed Organic Synthesis,” European Journal of Organic Chemistry 2017 (2017): 2056–2071. 30147436 10.1002/ejoc.201601485 PMC6099384

[24] C. R. J. Stephenson , T. P Yoon , D. W. C. MacMillan , Visible Light Photocatalysis in Organic Chemistry, (Wiley‐VCH Verlag GmbH & Co. KGaA, 2018).

[25] G. Ciamician , “The Photochemistry of the Future,” Science 36 (1912): 385–394. 17836492 10.1126/science.36.926.385

[26] H. Li , L. Zhou , Y. Weng , and X. Yang , “Photocatalytic and Electrocatalytic C–H Functionalization: Sustainable Pathways for Polymer Upcycling,” ChemSusChem 18 (2025): e202501091. 40716032 10.1002/cssc.202501091

[27] D. Xu , H. Wang , K. Zhang , Z. Ya , H. Wang , and S. Zhang , “Photocatalytic Waste Polystyrene Plastic Conversion: Reaction Mechanism and Catalyst Design,” Environmental Science & Technology 59 (2025): 16112–16129. 40719423 10.1021/acs.est.5c07160

[28] T. Uekert , C. M. Pichler , T. Schubert , and E. Reisner , “Solar‐Driven Reforming of Solid Waste for a Sustainable Future,” Nature Sustainability 4 (2021): 383–391.

[29] L. Wimberger , G. Ng , and C. Boyer , “Light‐Driven Polymer Recycling to Monomers and Small Molecules,” Nature Communications 15 (2024): 2510. 10.1038/s41467-024-46656-3 PMC10954676 38509090

[30] B. Ranby and J. Lucki , “New Aspects of Photodegradation and Photooxidation of Polystyrene,” Pure and Applied Chemistry 52 (1980): 295–303.

[31] I. Mita , T. Takagi , K. Horie , and Y. Shindo , “Photosensitized Degradation of Polystyrene by Benzophenone in Benzene Solution,” Macromolecules 17 (1984): 2256–2260.

[32] M. Wang , J. Wen , Y. Huang , and P. Hu , “Selective Degradation of Styrene‐Related Plastics Catalyzed by Iron under Visible Light,” ChemSusChem 14 (2021): 5049–5056. 34510789 10.1002/cssc.202101762

[33] G. Zhang , Z. Zhang , and R. Zeng , “Photoinduced FeCl_3_‐Catalyzed Alkyl Aromatics Oxidation toward Degradation of Polystyrene at Room Temperature,” Chinese Journal of Chemistry 39 (2021): 3225–3230.

[34] S. Oh and E. E. Stache , “Chemical Upcycling of Commercial Polystyrene via Catalyst‐Controlled Photooxidation,” Journal of the American Chemical Society 144 (2022): 5745–5749. 35319868 10.1021/jacs.2c01411

[35] S. Oh and E. E. Stache , “Mechanistic Insights Enable Divergent Product Selectivity in Catalyst‐Controlled Photooxidative Degradation of Polystyrene,” Acs Catalysis 13 (2023): 10968–10975.

[36] B. Gautam , M.‐R. Huang , C.‐C. Lin , C.‐C. Chang , and J.‐T. Chen , “A Viable Approach for Polymer Upcycling of Polystyrene (Styrofoam) Wastes to Produce High Value Predetermined Organic Compounds,” Polymer Degradation and Stability 217 (2023): 110528.

[37] Z. Xu , F. Pan , M. Sun , et al., “Cascade Degradation and Upcycling of Polystyrene Waste to High‐Value Chemicals,” Proceedings of the National Academy of Sciences of the United States of America 119 (2022): e2203346119. 35969757 10.1073/pnas.2203346119 PMC9407675

[38] J. Meng , Y. Zhou , D. Li , and X. Jiang , “Degradation of Plastic Wastes to Commercial Chemicals and Monomers under Visible Light,” Science Bulletin 68 (2023): 1522–1530. 37423865 10.1016/j.scib.2023.06.024

[39] S.‐B. Tang , Y.‐X. Jiang , K. Li , Z.‐X. Wang , and J. Su , “Uranyl Photocatalyzed Polystyrene Oxidative Degradation under Visible Light in a Green Solvent,” Industrial & Engineering Chemistry Research 63 (2024): 4817–4824.

[40] C. Li , X. Y. Kong , M. Lyu , et al., “Upcycling of Non‐Biodegradable Plastics by Base Metal Photocatalysis,” Chem 9 (2023): 2683–2700.

[41] E. Xu , T. Liu , F. Xie , J. He , and Y. Zhang , “Aerobic Oxidation of Alkylarenes and Polystyrene Waste to Benzoic Acids via a Copper‐Based Catalyst,” Chemical Science 16 (2025): 2004–2014. 39759934 10.1039/d4sc03269a PMC11696680

[42] T. Li , A. Vijeta , C. Casadevall , A. S. Gentleman , T. Euser , and E. Reisner , “Bridging Plastic Recycling and Organic Catalysis: Photocatalytic Deconstruction of Polystyrene via a C–H Oxidation Pathway,” Acs Catalysis 12 (2022): 8155–8163. 35874621 10.1021/acscatal.2c02292 PMC9295126

[43] Z. Huang , M. Shanmugam , Z. Liu , et al., “Chemical Recycling of Polystyrene to Valuable Chemicals via Selective Acid‐Catalyzed Aerobic Oxidation under Visible Light,” Journal of the American Chemical Society 144 (2022): 6532–6542. 35353526 10.1021/jacs.2c01410 PMC9011358

[44] Y. Qin , T. Zhang , H. Y. V. Ching , G. S. Raman , and S. Das , “Integrated Strategy for the Synthesis of Aromatic Building Blocks via Upcycling of Real‐Life Plastic Wastes,” Chem 8 (2022): 2472–2484.

[45] N. F. Nikitas , E. Skolia , P. L. Gkizis , I. Triandafillidi , and C. G. Kokotos , “Photochemical Aerobic Upcycling of Polystyrene Plastics to Commodity Chemicals Using Anthraquinone as the Photocatalyst,” Green Chemistry 25 (2023): 4750–4759.

[46] N. Xu , X. Peng , C. Luo , et al., “Light‐Promoted Chlorine‐Radical‐Mediated Oxidation of Benzylic C(sp^3^)−H Bonds Utilizing Air as Oxidant,” Advanced Synthesis & Catalysis 365 (2023): 142–147.

[47] E. Skolia , O. G. Mountanea , and C. G. Kokotos , “Photochemical Aerobic Upcycling of Polystyrene Plastics,” ChemSusChem 17 (2024): e202400174. 38763906 10.1002/cssc.202400174

[48] O. G. Mountanea , E. Skolia , and C. G. Kokotos , “Photochemical Aerobic Upcycling of Polystyrene Plastics via Synergistic Indirect HAT Catalysis,” Chemistry – A European Journal 30 (2024): e202401588. 38837489 10.1002/chem.202401588

[49] Y. B. Cakir , R. T. Uzun , H. C. Kiliclar , et al., “Efficient Photolytic Breakdown of Waste Polystyrene Foam Using an “All‐in‐One” Photo‐HAT Reagent at Ambient Conditions,” Acs Sustainable Chemistry & Engineering 12 (2024): 9978–9986.

[50] A. Ong , Z. C. Wong , K. L. O. Chin , et al., “Enhancing the Photocatalytic Upcycling of Polystyrene to Benzoic Acid: A Combined Computational‐Experimental Approach for Acridinium Catalyst Design,” Chemical Science 15 (2024): 1061–1067. 38239702 10.1039/d3sc06388g PMC10793207

[51] A.‐L. De Abreu , D. Taton , and D. M. Bassani , “Reassessing the Photochemical Upcycling of Polystyrene Using Acridinium Salts,” Angewandte Chemie International Edition 64 (2025): e202418680. 39535325 10.1002/anie.202418680 PMC11796324

[52] S. Zhang , J. Wang , D. Su , and X. Xiao , “Facile Visible‐Light Upcycling of Diverse Waste Plastics Using a Single Organocatalyst with Minimal Loadings,” Nature Communications 16 (2025): 4188. 10.1038/s41467-025-59540-5 PMC12056016 40328800

[53] L. H. Kugelmass , C. Tagnon , and E. E. Stache , “Photothermal Mediated Chemical Recycling to Monomers via Carbon Quantum Dots,” Journal of the American Chemical Society 145 (2023): 16090–16097. 37432654 10.1021/jacs.3c04448

[54] S. Oh , H. Jiang , L. H. Kugelmass , and E. E. Stache , “Recycling of Post‐Consumer Waste Polystyrene Using Commercial Plastic Additives,” Acs Central Science 11 (2025): 57–65. 39866706 10.1021/acscentsci.4c01317 PMC11758496

[55] For full optimization and mechanistic studies, see the ESI†.

[56] E. Voutyritsa , A. Theodorou , and M. G. Kokotou C. G. Kokotos , “Organocatalytic Oxidation of Substituted Anilines to Azoxybenzenes and Nitro Compounds: Mechanistic Studies Excluding the Involvement of a Dioxirane Intermediate,” Green Chemistry 19 (2017): 1291–1298.

[57] I. Triandafillidi , M. G. Kokotou , and C. G. Kokotos , “Photocatalytic Synthesis of *γ*‐Lactones from Alkenes: High‐Resolution Mass Spectrometry as a Tool to Study Photoredox Reactions,” Organic Letters 20 (2018): 36–39. 29215290 10.1021/acs.orglett.7b03256

[58] I. Triandafillidi , M. G. Kokotou , D. Lotter , C. Sparr , and C. G. Kokotos , “Aldehyde‐Catalyzed Epoxidation of Unactivated Alkenes with Aqueous Hydrogen Peroxide,” Chemical Science 12 (2021): 10191–10196. 34377408 10.1039/d1sc02360h PMC8336450

[59] O. G. Mountanea , D. Psathopoulou , C. Mantzourani , et al., “An Efficient Light‐Mediated Protocol for the Direct Amide Bond Formation via a Novel Carboxylic Acid Photoactivation Mode by Pyridine‐CBr_4_ ,” Chemistry – A European Journal 29 (2023): e202300556. 37015030 10.1002/chem.202300556

[60] G. E. M. Crisenza , D. Mazzarella , and P. Melchiorre , “Synthetic Methods Driven by the Photoactivity of Electron Donor–Acceptor Complexes,” Journal of the American Chemical Society 142 (2020): 5461–5476. 32134647 10.1021/jacs.0c01416 PMC7099579

[61] T. K. Dinda , A. Manna , and P. Mal , “En Route to Recyclable Semi‐Heterogeneous Photocatalysis with Photoinert CeCl_3_ ,” Acs Catalysis 14 (2024): 7664–7673.

[62] S. Sahoo , T. K. Dinda , and P. Mal , “ *N*‐Halosuccinimide‐CeCl_3_ Transient Charge‐Transfer Complexes as Semi Heterogeneous Photocatalyst in Cyclization of N‐Propargylamides,” Chemistry – A European Journal 30 (2024): e202402192. 39087763 10.1002/chem.202402192

[63] Y. Luo , S. Zhou , A. A. Nkingwa , and Q. Zeng , “Indium(III)‐Catalyzed Three Component Reaction of *N*‐Bromosuccinimide, Alkenes and N‐Tosylhydrazones,” European Journal of Organic Chemistry 27 (2024): e202301178.

[64] Y. Yu and M. Kazemi , “Indium Bromide (InBr_3_): A Versatile and Efficient Catalyst Inorganic Synthesis,” Synthetic Communications 51 (2021): 2574–2601.

[65] F. Mo , J. M. Yan , D. Qiu , F. Li , Y. Zhang , and J. Wang , “Gold‐Catalyzed Halogenation of Aromatics by *N*‐Halosuccinimides,” Angewandte Chemie International Edition 49 (2010): 2028–2032. 20155774 10.1002/anie.200906699

[66] G. K. S. Prakash , T. Mathew , D. Hoole , et al., “ *N*‐Halosuccinimide/BF_3_−H_2_O, Efficient Electrophilic Halogenating Systems for Aromatics,” Journal of the American Chemical Society 126 (2004): 15770–15776. 15571400 10.1021/ja0465247

[67] S. Guha , I. Kazi , A. Nandy , and G. Sekar , “Role of Lewis‐Base‐Coordinated Halogen(I) Intermediates in Organic Synthesis: The Journey from Unstable Intermediates to Versatile Reagents,” European Journal of Organic Chemistry 2017 (2017): 5497–5518.

[68] M. A. B. Mostafa , R. M. Bowley , D. T. Racys , M. C. Henry , and A. Sutherland , “Iron(III)‐Catalyzed Chlorination of Activated Arenes,” The Journal of Organic Chemistry 82 (2017): 7529–7537. 28661157 10.1021/acs.joc.7b01225

[69] R. B. Hochberg , “Biological Esterification of Steroids,” Endocrine Reviews 19 (1998): 331–348. 9626557 10.1210/edrv.19.3.0330

[70] P. R. C. Corrêa , R. R. S. Miranda , L. P. Duarte , et al., “Antimicrobial Activity of Synthetic Bornyl Benzoates Against *Trypanosoma Cruzi* ,” Pathogens and Global Health 106 (2012): 107–112. 22943546 10.1179/2047773212Y.0000000002 PMC4001496

[71] M. Sagransky , B. A. Yentzer , and S. R. Feldman , “Benzoyl Peroxide: A Review of Its Current Use in the Treatment of Acne Vulgaris,” Expert Opinion on Pharmacotherapy 10 (2009): 2555–2562. 19761357 10.1517/14656560903277228

[72] E. A. Tanghetti and K. F. Popp , “A Current Review of Topical Benzoyl Peroxide: New Perspectives on Formulation and Utilization,” Dermatologic Clinics 27 (2009): 17–24. 18984364 10.1016/j.det.2008.07.001

[73] L.‐X. Shao and M. Shi , “ *N*‐Bromosuccinimide and Lithium Bromide: An Efficient Combination for the Dibromination of Carbon‐Carbon Unsaturated Bonds,” Synlett 8 (2006): 1269–1271.

[74] Z. Zhang , H. Fattal , T. D. Creason , et al., “Investigation of the Solution Chemistry of Hybrid Organic–Inorganic Indium Halides for New Material Discovery,” Inorganic Chemistry 61 (2022): 13015–13021. 35944017 10.1021/acs.inorgchem.2c01161

[75] C. Coenjarts , F. Ortica , J. Cameron , et al., “Mechanism of Reaction and Photoacid Generation of 1,2‐Di(Arylsulfonyl)hydrazine PAGs: A Laser Flash Photolytic Study,” Chemistry of Materials 13 (2001): 2305–2312.

[76] A. M. May and J. L. Dempsey , “A New Era of LMCT: Leveraging Ligand‐to‐Metal Charge Transfer Excited States for Photochemical Reactions,” Chemical Science 15 (2024): 6661–6678. 38725519 10.1039/d3sc05268k PMC11079626

[77] S. M. Treacy and T. Rovis , “Photoinduced Ligand‐to‐Metal Charge Transfer in Base‐Metal Catalysis,” Synthesis 56 (2024): 1967–1978. 38962497 10.1055/s-0042-1751518 PMC11218547

[78] A. A. Gladkov , V. V. Levin , D. Y. Cheboksarov , and A. D. Dilman , “Unlocking the Reactivity of the C–In Bond: Alkyl Indium Reagents as a Source of Radicals under Photocatalytic Conditions,” Chemical Science 16 (2025): 5623–5631. 40041808 10.1039/d4sc08521c PMC11873600

[79] T. E. Hurst , J. A. Deichert , L. Kapeniak , et al., “Sodium Methyl Carbonate as an Effective C1 Synthon. Synthesis of Carboxylic Acids, Benzophenones, and Unsymmetrical Ketones,” Organic Letters 21 (2019): 3882–3885. 31125244 10.1021/acs.orglett.9b00773

[80] S. Cailotto , M. Negrato , S. Daniele , et al., “Carbon Dots as Photocatalysts for Organic Synthesis: Metal‐Free Methylene–oxygen‐Bond Photocleavage,” Green Chemistry 22 (2020): 1145–1149.

[81] A. Modak , T. Naveen , and D. Maiti , “An Eficient Dehydroxymethylation Reaction by a Palladium Catalyst,” Chemical Communications 49 (2013): 252–254. 23164948 10.1039/c2cc36951f

[82] Y. Inoue , S. Takamuku , Y. Kunitomi , and H. Sakurai , “Singlet Photosensitization of Simple Alkenes. Part 1. cis–trans‐Photoisomerization of Cyclo‐Octene Sensitized by Aromatic Esters,” Journal of the Chemical Society, Perkin Transactions 2 (1980): 1672–1677.

[83] S. Kamijo , K. Tao , G. Takao , H. Tonoda , and T. Murafuji , “Photoinduced Oxidation of Secondary Alcohols Using 4‐Benzoylpyridine as an Oxidant,” Organic Letters 17 (2015): 3326–3329. 26099050 10.1021/acs.orglett.5b01550

[84] D. H. R. Barton and J. D. Cox , “158. The Application of the Method of Molecular Rotation Differences to Steroids. Part IV. Optical Anomalies,” Journal of the Chemical Society (1948): 783–793. 18871272 10.1039/jr9480000783

[85] B. Lyu , Y. Hiraga , R. Takagi , and S. Niwayama , “Complete Assignments of ^1^H and ^13^C NMR Chemical Shift Changes Observed upon Protection of Hydroxy Group in Borneol and Isoborneol and Their DFT Verification,” Molecules 30 (2025): 597. 39942701 10.3390/molecules30030597 PMC11819931

[86] N. Kunieda , A. Suzuki , and M. Kinoshita , “The Preparation of Optically Active *β*‐Keto Sulfoxides by Means of an Enantiomer‐Differentiating Reaction of *α*‐Lithio Aryl Methyl Sulfoxides with Chiral Carboxylates,” Bulletin of the Chemical Society of Japan 54 (1981): 1143–1150.

[87] N. Yadav , S. R. Bhatta , and J. N. Moorthy , “Visible Light‐Induced Decomposition of Acyl Peroxides Using Isocyanides: Synthesis of Heteroarenes by Radical Cascade Cyclization,” The Journal of Organic Chemistry 88 (2023): 5431–5439. 37093050 10.1021/acs.joc.2c03059

[88] F. D. Greene and J. Kazan , “Preparation of Diacyl Peroxides with N, N’‐Dicyclohexylcarbodiimide,” The Journal of Organic Chemistry 28 (1963): 2168–2171.

