# Supplementary material for: Photochemical Aerobic Upcycling of Polystyrene Plastics via Indium Salt‐Mediated Synergistic Catalysis
Source: ChemSusChem. 2026 Mar 3;19(5):e202502759. doi: 10.1002/cssc.202502759 (PMC12954651; doi:10.1002/cssc.202502759)
Supplement: Supplementary file 1 — Supplementary Material [file CSSC-19-e202502759-s001.pdf]

Photochemical Aerobic Upcycling of Polystyrene Plastics via Indium Salt-Mediated Synergistic Catalysis

Lydia Lavrenti, Christos Papagkikas, Olga G. Mountanea* and Christoforos G. Kokotos*

Laboratory of Organic Chemistry, Department of Chemistry, National and Kapodistrian University of Athens, Panepistimiopolis, Athens 15771, Greece

SUPPORTING INFORMATION

	Page
**General Remarks**	**S3**
**Photochemical Aerobic Upcycling of Polystyrene: Photochemical System Study – Study of Metal Chlorides**	**S4**
**Photochemical Aerobic Upcycling of Polystyrene: Photochemical System Study – Study of Metal Bromides**	**S5**
**Photochemical Aerobic Upcycling of Polystyrene: Irradiation Source Study**	**S6**
**Photochemical Aerobic Upcycling of Polystyrene: Investigation of the Effect of Solvent Volume**	**S7**
**Photochemical Aerobic Upcycling of Polystyrene: Solvent Study**	**S8**
**Photochemical Aerobic Upcycling of Polystyrene: Halogen Source – Halogen Source Loading Study**	**S9**
**Photochemical Aerobic Upcycling of Polystyrene: $InBr_3$ Loading Study**	**S10**
**Photochemical Aerobic Upcycling of Polystyrene: Reaction Time Study**	**S11**
**Photochemical Aerobic Upcycling of Polystyrene: Control Experiments**	**S12**
**Photochemical Aerobic Upcycling of Polystyrene: Mechanistic Experiments**	**S13**
**General Procedure for the Photochemical Aerobic Upcycling of Commercially Available Polystyrene**	**S14**
**Application of the Photochemical Aerobic Upcycling of Polystyrene to Plastic Polystyrene Daily Products**	**S19**
**Large Scale Photochemical Aerobic Upcycling of Polystyrene**	**S32**
**Synthesis of Butane-1,3-diyldibenzene (5) for Mechanistic Studies**	**S37**
**Direct Infusion-High Resolution Mass Spectrometry (DI-HRMS) Mechanistic Studies**	**S40**
**Further Mechanistic Studies**	**S74**
**Mechanistic Studies: UV-Vis Spectra**	**S78**
**Mechanistic Studies: NMR Spectra Studies**	**S86**
**Further Functionalization Reactions of Benzoic Acid**	**S92**
**References**	**S96**
**NMR Spectra**	**S97**

General Remarks

Chromatographic purification of products was accomplished using forced-flow chromatography on Merck® Kieselgel 60 230-400 mesh. Thin-layer chromatography (TLC) was performed on aluminum backed silica plates (0.2 mm, 60 F_{254}). Visualization of the developed chromatograms was performed by fluorescence quenching using phosphomolybdic acid, anisaldehyde or potassium permanganate stains. Melting points were determined on a Buchi® 530 hot stage apparatus and are uncorrected. Mass spectra (ESI) were recorded on a Finningan® Surveyor MSQ LC-MS spectrometer. HRMS spectra were recorded on Bruker® Maxis Impact QTOF spectrometer. Optical rotations were measured using a PerkinElmer 343 or an AA-65 series polarimeter in a 10 cm cell at room temperature. Sonication of the reaction mixture was performed in a ISOLAB 621.05.010 Sonicator (Ultrasonic Power 240 W). ^{1}H- and ^{13}C-NMR spectra were recorded on a Brucker Avance-400 (400 MHz and 100 MHz) spectrometer in $CDCl_3$ and are internally referenced to residual solvent signals. Data for ^{1}H-NMR are reported as follows: chemical shift (δ ppm), multiplicity (s = singlet, d = doublet, t = triplet, q = quartet, m = multiplet, br s = broad signal), coupling constant, integration and assignment. Data for ^{13}C-NMR are reported in terms of chemical shift (δ ppm). Kessil lamps PR160L were used as the irradiation source. For all experiments, the intensity of Kessil lamps was controlled in the maximum level with power consumption: 370 nm (max 43W), 370nm 2nd Gen (max 44W), 390 nm (max 52W), 400 nm (max 40W), 427 nm & 440 nm (max 45W), 456 nm (max 50W), 467 nm (max 44 W) and 525 nm (max 44W). The reaction under sunlight was performed in Athens, Greece, 37.97° N, 23.72° E.

Photochemical Aerobic Upcycling of Polystyrene

Photochemical System Study – Study of Metal Chlorides

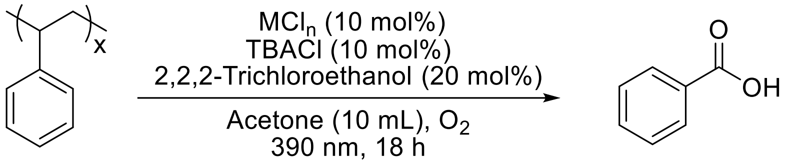

Entry	Metal Chlorides (MCl_n)	Yield (%)[a]
1	$FeCl_3$	14
2[b]	$FeCl_3$	5
3[c]	$FeCl_3$	16
4	$MnCl_2$	4
5[d]	$MnCl_2$	4
6	$NiCl_2$	2
7	$CoCl_2$•$6H_2O$	traces
8	$ZnCl_2$	2
9	CuCl	2
10	$BiCl_3$	3
11[d]	$BiCl_3$	5
12	$CeCl_3$•$7H_2O$	traces
13	$RuCl_3$	traces
14	$TiCl_3$	3
15	VCl_3	12
16[e]	VCl_3	18
17	$SnCl_2$•$2H_2O$	1

Table S1. [a] Yield of isolated product, after base-acid wash and extractions. [b] $FeCl_3$ (20 mol%), TBACl (20 mol%), without 2,2,2-trichloroethanol, acetone (5 mL), 2 Kessil lamps. [c] $FeCl_3$ (20 mol%), TBACl (20 mol%), without 2,2,2-trichloroethanol, 72 h. [d] Reaction time: 72 h. [e] Reaction time: 48 h.

Photochemical Aerobic Upcycling of Polystyrene

Photochemical System Study – Study of Metal Bromides

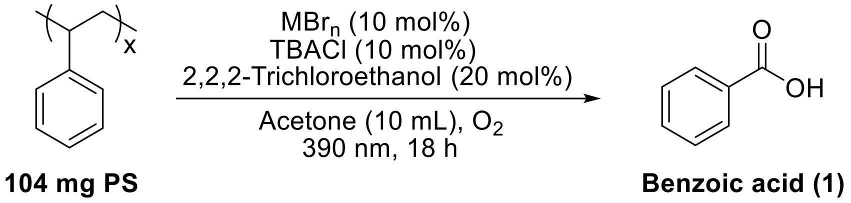

Entry	Metal Bromides (MBr_n)	Yield (%)[a]
1	$MnBr_2$	1
2	$NiBr_2$•dme	1
3	$CoBr_2$	1
4	$ZnBr_2$	1
5	$CuBr_2$	1
6	LiBr	2
7	$InBr_3$	11
8[b]	$InBr_3$	30

Table S2. [a] Yield of isolated product, after base-acid wash and extractions. [b] Reaction time: 72 h.

Photochemical Aerobic Upcycling of Polystyrene

Irradiation Source Study

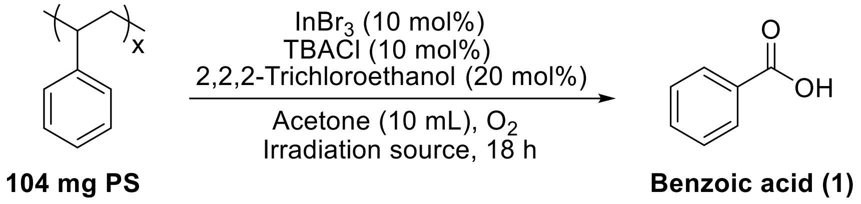

Entry	Irradiation Source (nm)	Yield (%)[a]
1	CFL	traces
2	370	2
3	370 (2nd gen)	20
4[b]	370 (2nd gen)	15
5[c]	370 (2nd gen)	27
6[b,c]	370 (2nd gen)	31
7	390	11
8[b,c]	390	3
9[c]	390	30
10	400	7
11	427	traces
12	440	traces
13	456	traces
14	467	traces
15	525	traces

Table S3. [a] Yield of isolated product, after base-acid wash and extractions. [b] Reaction under air. [c] Reaction time: 72 h.

Photochemical Aerobic Upcycling of Polystyrene

Investigation of the Effect of Solvent Volume

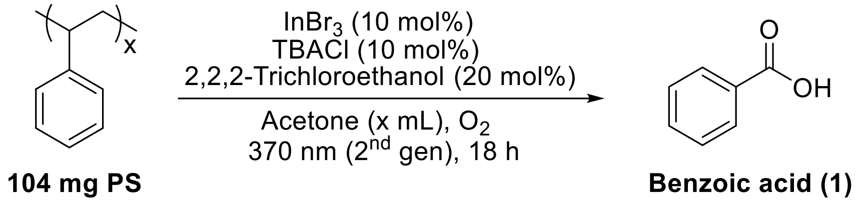

Entry	Acetone (mL)	Yield (%)[a]
1	2	1
2	4	10
3	5	22
4	6	17
5	8	20
6	10	20

Table S4. [a] Yield of isolated product, after base-acid wash and extractions.

Photochemical Aerobic Upcycling of Polystyrene

Solvent Study

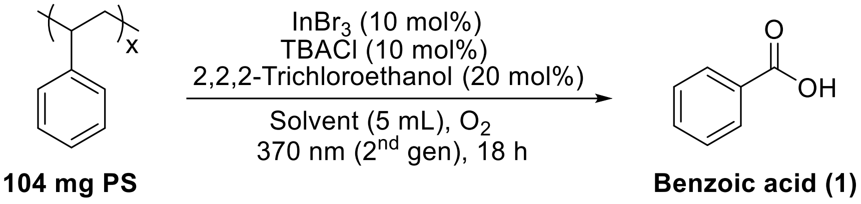

Entry	Solvent	Yield (%)[a]
1	Acetone	22
2	EtOAc	23
3	$CHCl_3$	20
4	Acetone/EtOAc (1/1)	19
5	CH_2Cl_2	3
6	ACN	traces
7	DMF	1
8	DMSO	traces
9	Benzene	3

Table S5. [a] Yield of isolated product, after base-acid wash and extractions.

Photochemical Aerobic Upcycling of Polystyrene

Halogen Source – Halogen Source Loading Study

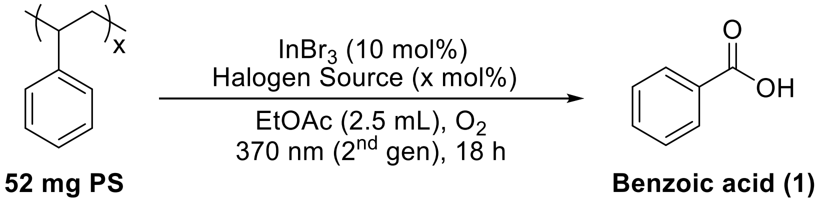

Entry	Halogen Source	Halogen Source Loading (mol%)	Yield (%)[a]
1	TBACl/2,2,2-trichloroethanol	10/20	23
2	TBACl	10	30
3	TBACl	20	4
4	TBABr	10	17
5	TBABr	20	4
6	TsCl	10	30
7	TsCl	20	4
8	TsBr	10	39
9	TsBr	20	40
10	NBS	10	39
11	NBS	20	41
12	TBABr/TsCl (1/1)	10/10	33
13	TBABr/TsBr (1/1)	10/10	31
14	TBABr/NBS (1/1)	10/10	37
15	TBACl/TsCl (1/1)	10/10	20
16	TBACl/TsBr (1/1)	10/10	16
17	TBACl/NBS (1/1)	10/10	14
18	TsCl/NBS (1/1)	10/10	43
19	TsBr/NBS (1/1)	10/10	51
20	TsBr/NBS (1/1)	5/5	14
21	TsBr/NBS (1/1)	15/15	26
22	TsBr/NBS (3/1)	15/5	36
23	TsBr/NBS (1/3)	5/15	39

Table S6. [a] Yield of isolated product, after base-acid wash and extractions.

Photochemical Aerobic Upcycling of Polystyrene

$InBr_3$ Loading Study

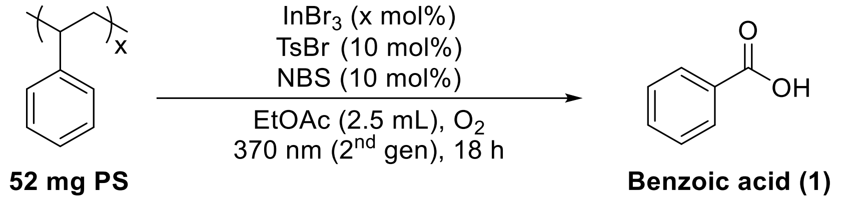

Entry	$InBr_3$ Loading (mol%)	Yield (%)[a]
1	5	24
2	10	51
3	15	38

Table S7. [a] Yield of isolated product, after base-acid wash and extractions.

Photochemical Aerobic Upcycling of Polystyrene

Reaction Time Study

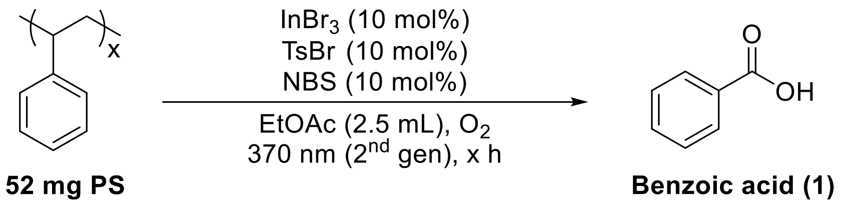

Entry	Reaction Time (h)	Yield (%)[a]
1	6	36
2[b]	6	42
3[c]	6	26
4	18	51
5	72	43

Table S8. [a] Yield of isolated product, after base-acid wash and extractions. [b] Polystyrene (MW: 390.000) was used as the starting material. [c] Irradiation under 2 x 2nd gen Kessil lamps.

Photochemical Aerobic Upcycling of Polystyrene

Control Experiments

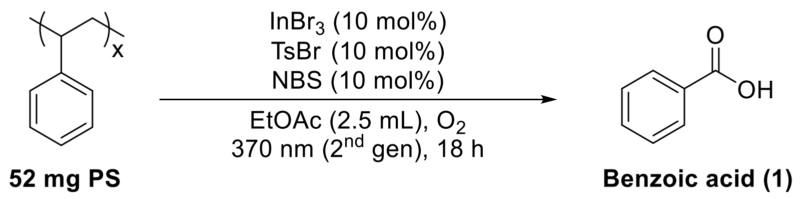

Entry	Control Variations	Yield (%)[a]
1	Under dark	traces
2	Under sunlight	1
3	No hv, at 45 °C	0
4	370 nm (2nd gen) irradiation, at 45 °C	19
5[b]	18 h irradiation and 24 h without irradiation at 140 °C after addition of $Cu(NO_3)_2•3H_2O$ (20 mol%)	40
6	Under air	25
7	Under argon	3
8	In the presence of 14 mg of a HD-PE plastic bottle	35
9	In the presence of 14 mg of a LD-PE plastic bag	34
10	In the presence of 21 mg of a PP plastic cup	38
11	In the presence of 96 mg of a PET water bottle	39

Table S9. [a] Yield of isolated product, after base-acid wash and extractions. [b] After 18 h of irradiation, $Cu(NO_3)_2•3H_2O$ (20 mol%, 24.2 mg) in a mixture of EtOAc:MeCN (2:1.5 mL) was added and the reaction mixture was left under stirring at 140 °C for 24 h.

Photochemical Aerobic Upcycling of Polystyrene

Mechanistic Experiments

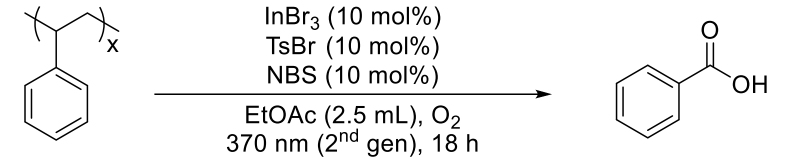

Entry	Quenchers – Mechanistic Variations	Yield (%)[a]
1	EtOAc [no $InBr_3$, TsBr, NBS]	3
2	$InBr_3$ [no TsBr, NBS]	19
3	NBS [no $InBr_3$, TsBr]	6
4	TsBr [no $InBr_3$, NBS]	10
5	Br_2 [no $InBr_3$, TsBr, NBS]	6
6	Br_2 and $InBr_3$ [no TsBr, NBS]	35
7	Br_2, $InBr_3$ and TsBr [no NBS]	50
8	Hydrogen peroxide (30 wt%) solution (10 equiv.) [no $InBr_3$, TsBr, NBS]	1
9	Addition of hydrogen peroxide (30 wt%) solution (10 equiv.)	19
10	Addition of NaN_3 (0.50 mmol)	1
11	Addition of DABCO (0.50 mmol)	traces

Table S10. [a] Yield of isolated product, after base-acid wash and extractions.

General Procedure for the Photochemical Aerobic Upcycling of Commercially Available Polystyrene

In a test tube containing polystyrene (52 mg, 0.50 mmol based on the repeating unit, CAS Number: 9052-95-3, 1.00 equiv.), indium tribromide ($InBr_3$) (18 mg, 0.05 mmol, 0.10 equiv.), tosyl bromide (TsBr) (12 mg, 0.05 mmol, 0.10 equiv.), *N*-bromosuccinimide (NBS) (9 mg, 0.05 mmol, 0.10 equiv.) and EtOAc (2.5 mL) were added. The reaction mixture was sealed with a septum and parafilm and was placed in an ultrasonic bath for 10 minutes. Then, a first degassing was performed using vacuum and argon flow, followed by a second degassing using oxygen flow. The atmosphere in the test tube was maintained by attaching two balloons filled with oxygen. The reaction mixture was stirred and irradiated with a 2nd generation Kessil lamp at 370 nm for 18 hours. After reaction completion, the reaction mixture was diluted with CH_2Cl_2 (2.5 mL) and filtered under vacuum to remove the insoluble residues corresponding to the starting material or lower molecular weight polymeric/oligomeric species. The filtrate was then concentrated *in vacuo.* Then, CH_2Cl_2 (5 mL) and aq. NaOH 1N (2.5 mL) were added to the flask and the crude reaction mixture was stirred for 10 min. The layers were separated in a separatory funnel. The aqueous phase was then acidified with concentrated HCl until pH = 1. Then, the aqueous layer was extracted with CH_2Cl_2 (3 x 5 mL) and the combined organic layers were dried over Na_2SO_4 and concentrated *in vacuo*, affording BA (**1**).

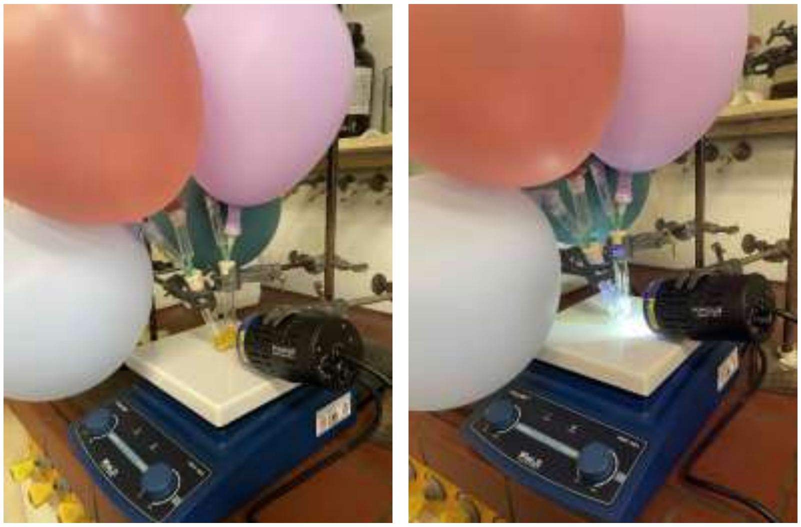

Figure S1. A: Reaction mixture setup, **B**: Reaction mixture during irradiation.

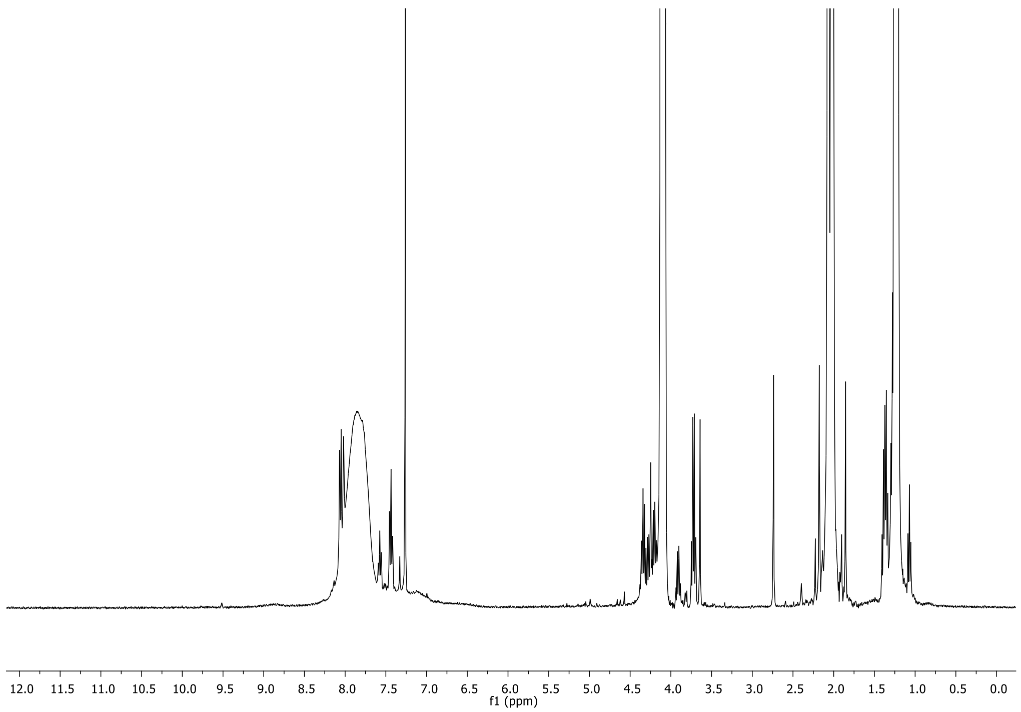

^{1}H NMR spectrum (400 MHz, $CDCl_3$) of the crude reaction mixture from the reaction before filtration and solvent evaporation.

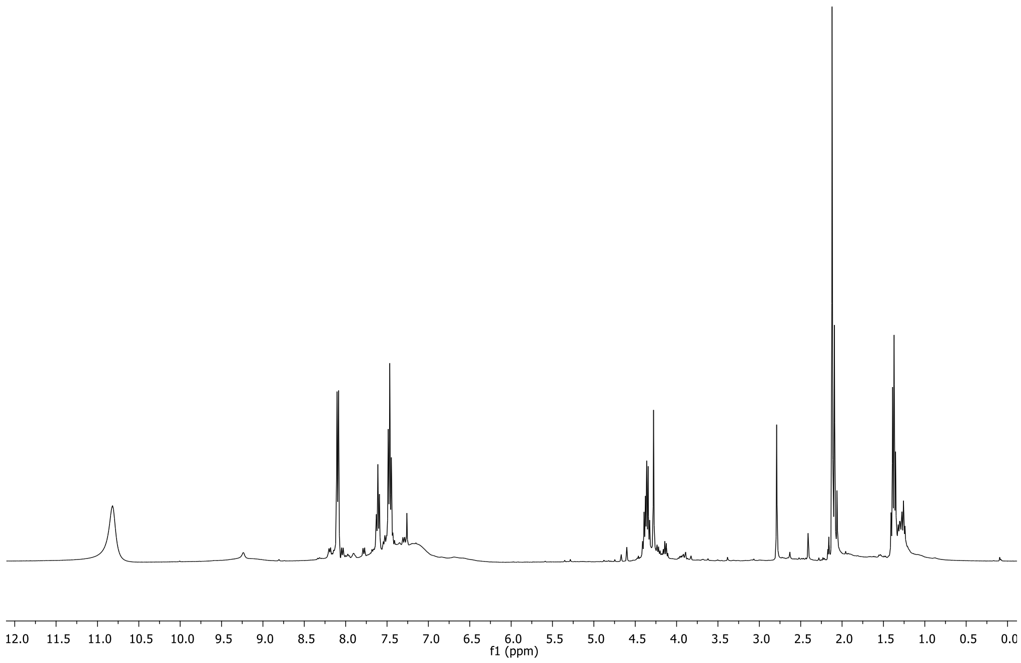

^{1}H NMR spectrum (400 MHz, $CDCl_3$) of the crude reaction mixture from the reaction after filtration and solvent evaporation.

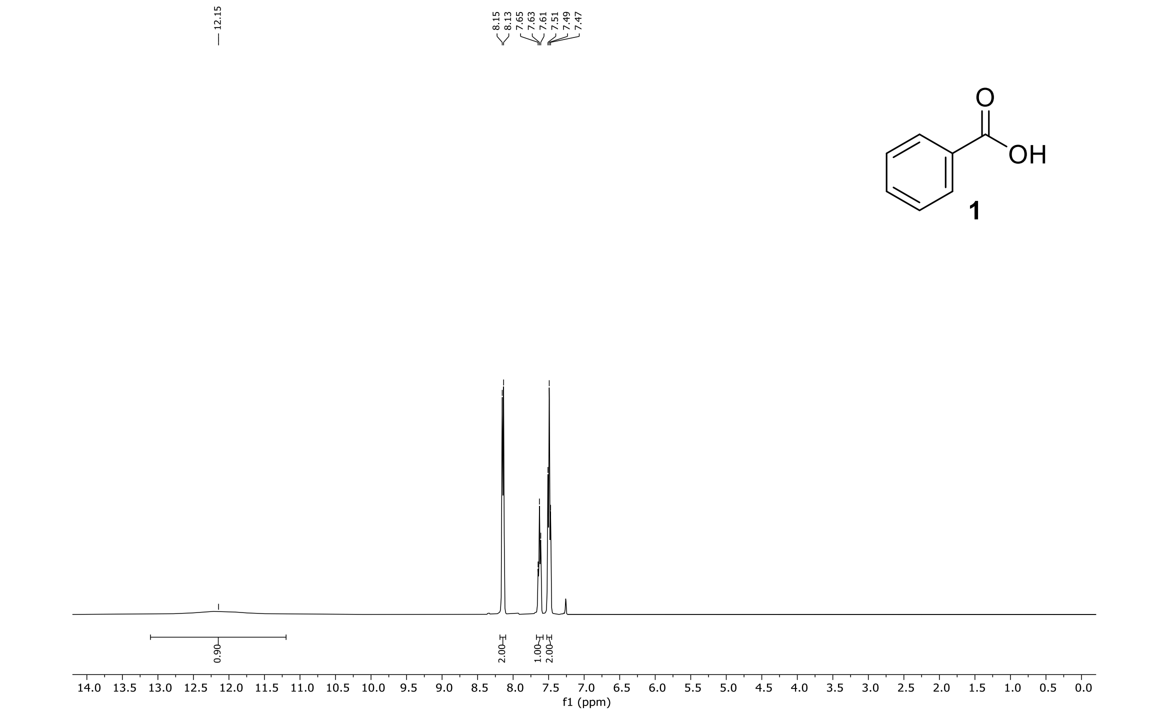

^{1}H NMR (400 MHz, $CDCl_3$) spectrum of the isolated benzoic acid (1) after acid-base workup

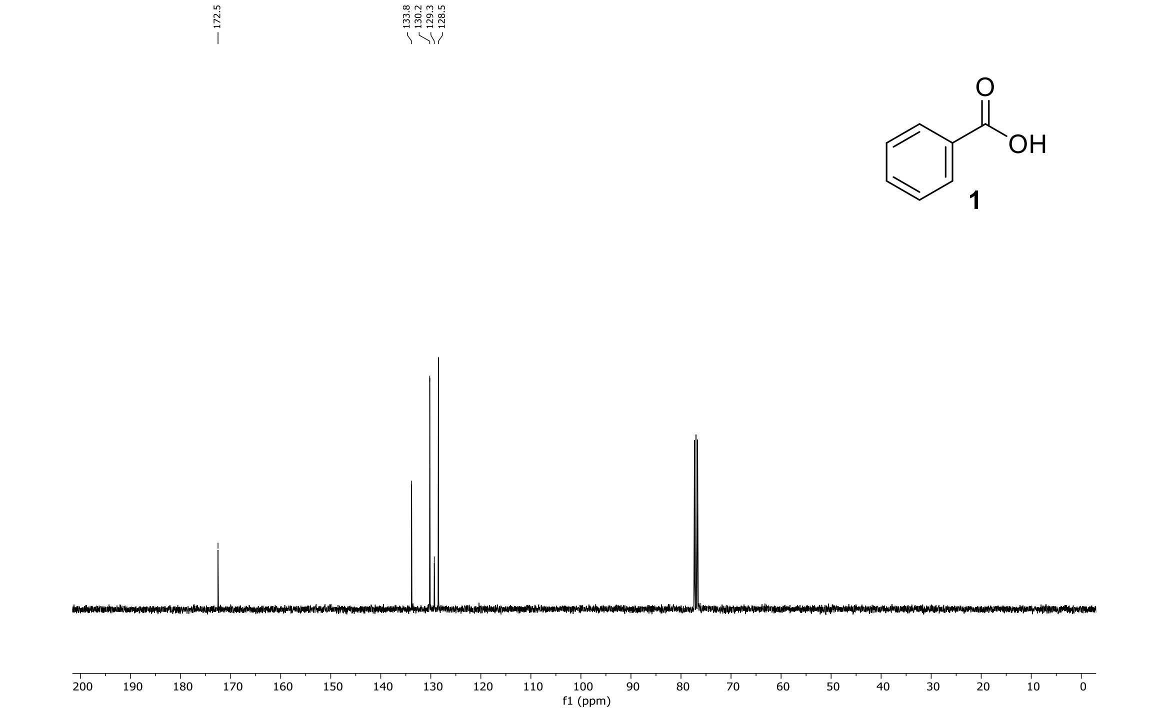

^{13}C NMR (100 MHz, $CDCl_3$) spectrum of the isolated benzoic acid (1) after acid-base workup

Benzoic acid (1)[1]

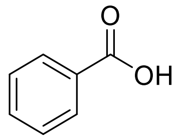

White solid; m.p.: 119-121 °C (lit. m.p.: 120-121 °C); **^{1}H NMR** ($CDCl_3$, 400 MHz): δ 12.15 (1H, br s, COOH), 8.14 (2H, d, *J* = 7.7 Hz, ArH), 7.63 (1H, t, *J* = 7.7 Hz, ArH), 7.49 (2H, t, *J* = 7.7 Hz, ArH); **^{13}C NMR** ($CDCl_3$, 100 MHz): δ 172.5, 133.8, 130.2, 129.3, 128.5; **MS (ESI)** m/z: 121 $[M-H]^-$.

Polystyrene Resins

Poly(styrene-co-divinylbenzene) (CAS Number: 9052-95-3)

200-400 mesh

Yield of benzoic acid (51%)

(Aminomethyl)polystyrene Resin (CAS Number: 89551-24-6)

100-200 mesh, HL, substitution: 0.81 mmol/g

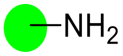

Yield of benzoic acid (21%)

Wang Resin (CAS Number: 65307-53-1)

extent of labeling ca. 1.0 mmol/g loading

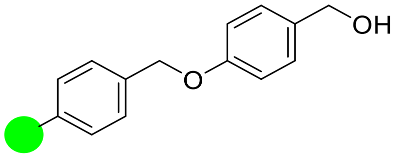

Yield of benzoic acid (11%)

Chlorotrityl Chloride Resin (CAS Number: 42074-68-0)

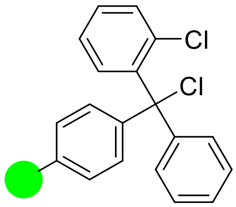

Yield of benzoic acid (24%)

Application of the Photochemical Aerobic Upcycling of Polystyrene to Plastic Polystyrene Daily Products

Polystyrene Red Cup

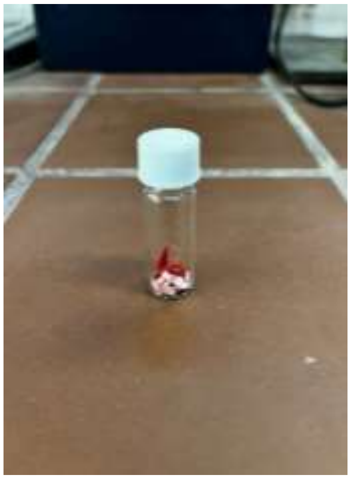

Polystyrene red cup (52 mg)

Yield of benzoic acid (41%)

Polystyrene Blue Cup

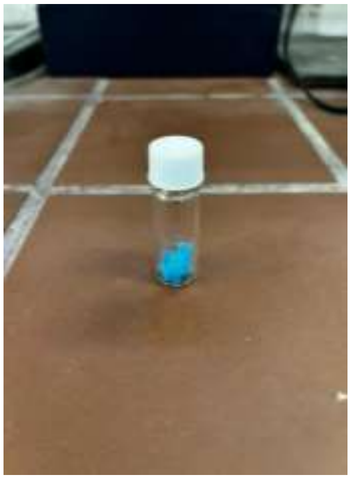

Polystyrene blue cup (52 mg)

Yield of benzoic acid (33%)

Polystyrene White Knife

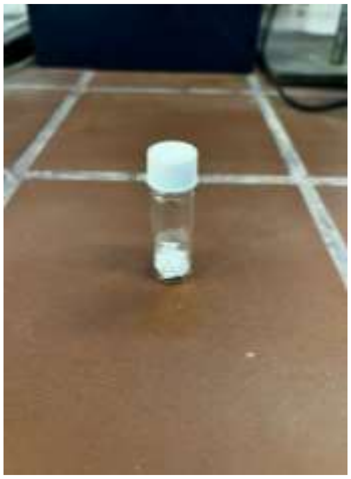

Polystyrene white knife (52 mg)

Yield of benzoic acid (37%)

Polystyrene Black Knife

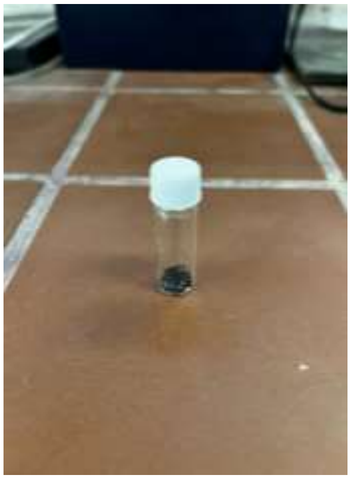

Polystyrene black knife (52 mg)

Yield of benzoic acid (36%)

Polystyrene Green Spoon

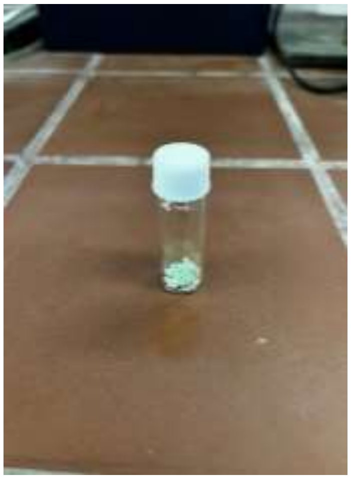

Polystyrene green spoon (52 mg)

Yield of benzoic acid (38%)

Black part of CD Case

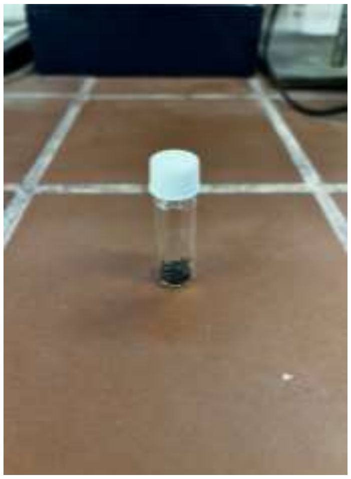

Polystyrene CD case – black part (52 mg)

Yield of benzoic acid (49%)

Transparent part of CD Case

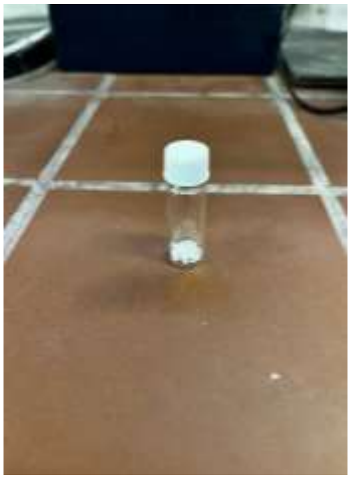

Polystyrene CD case – transparent part (52 mg)

Yield of benzoic acid (43%)

Polystyrene Green Foam

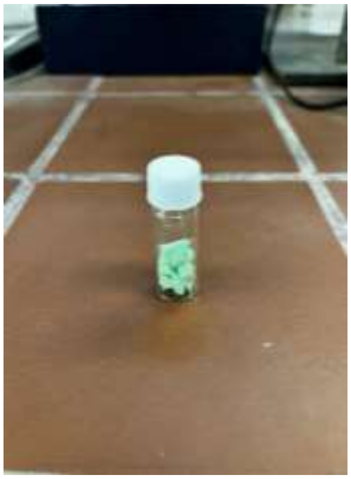

Polystyrene-based green foam (52 mg)

Yield of benzoic acid (27%)

Ice Cream Polystyrene Foam Container

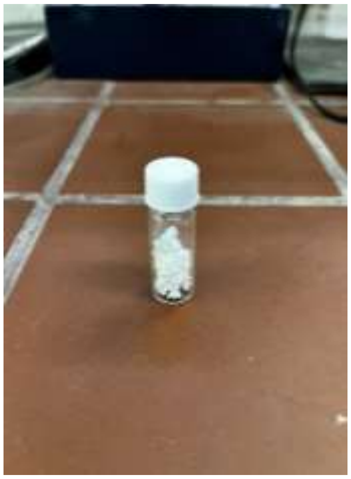

Polystyrene-based ice cream foam container (52 mg)

Yield of benzoic acid (41%)

Polystyrene Yellow Cup

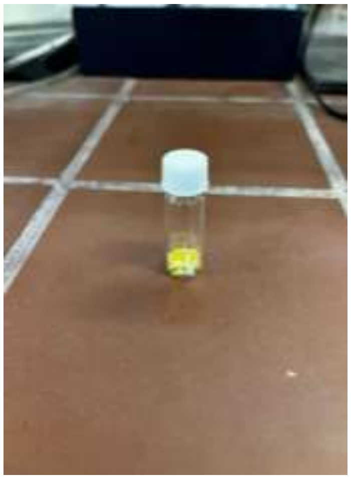

Polystyrene yellow cup (52 mg)

Yield of benzoic acid (23%)

Polystyrene Transparent Knife

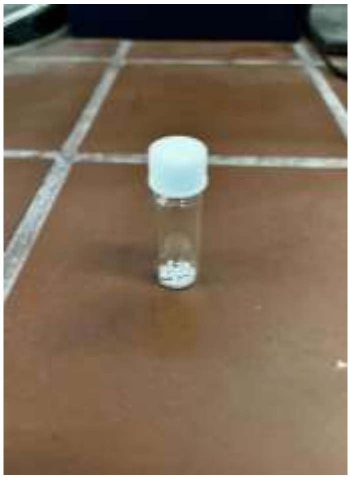

Polystyrene transparent knife (52 mg)

Yield of benzoic acid (45%)

Polystyrene Small Transparent Cup

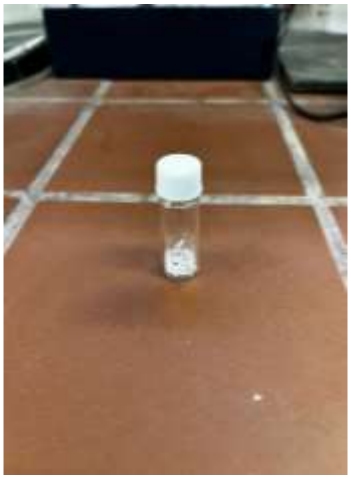

Polystyrene-based small transparent cup (52 mg)

Yield of benzoic acid (45%)

Polystyrene White Foam

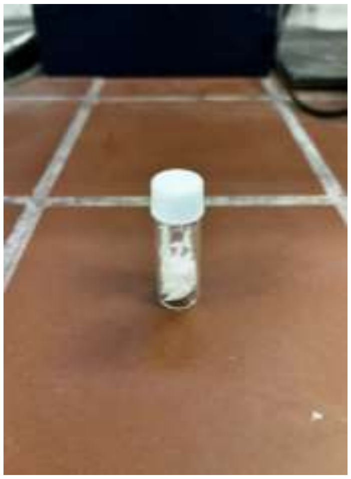

Polystyrene-based white foam (52 mg)

Yield of benzoic acid (46%)

Polystyrene White Foam Cubes

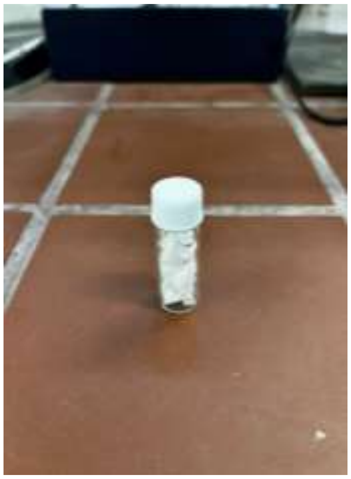

Polystyrene white foam cubes (52 mg)

Yield of benzoic acid (41%)

Polystyrene Black Coffee Cup Lid

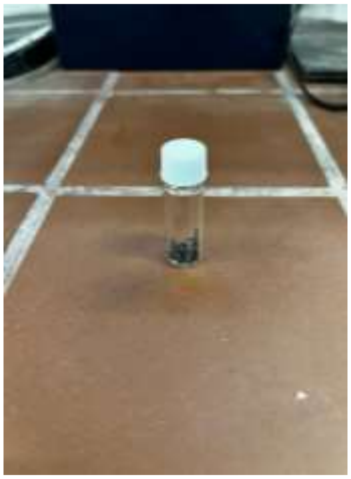

Polystyrene black coffee cup lid (52 mg)

Yield of benzoic acid (46%)

Polystyrene Transparent Frozen Drink Cup Lid

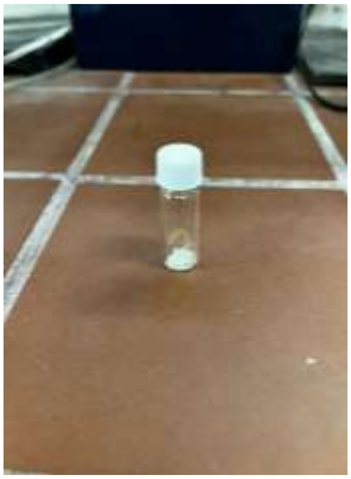

Polystyrene transparent frozen drink cup lid (52 mg)

Yield of benzoic acid (37%)

Polystyrene Black Part of Microwavable Food Container

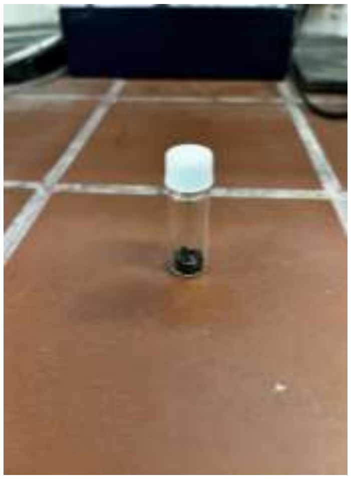

Polystyrene black part of microwavable food container (52 mg)

Yield of benzoic acid (14%)

Polystyrene Transparent Part of Microwavable Food Container

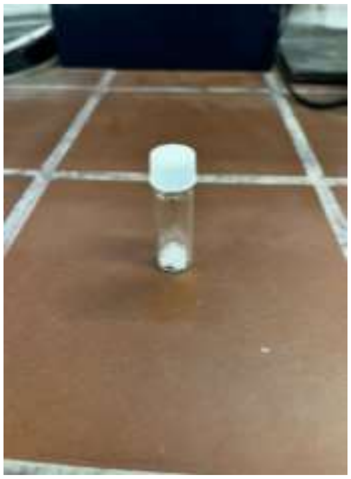

Polystyrene transparent part of microwavable food container (52 mg)

Yield of benzoic acid (49%)

Polystyrene Egg Storage Box

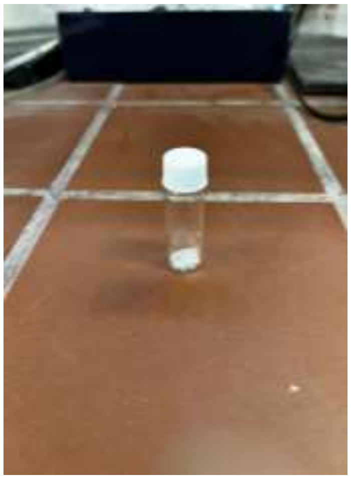

Polystyrene egg storage box (52 mg)

Yield of benzoic acid (50%)

Polystyrene Food Container

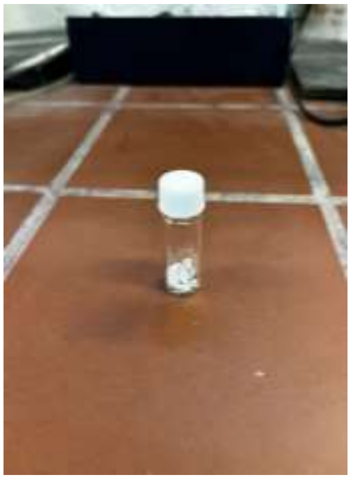

Polystyrene food container (52 mg)

Yield of benzoic acid (37%)

Polystyrene Foam Christmas Ball

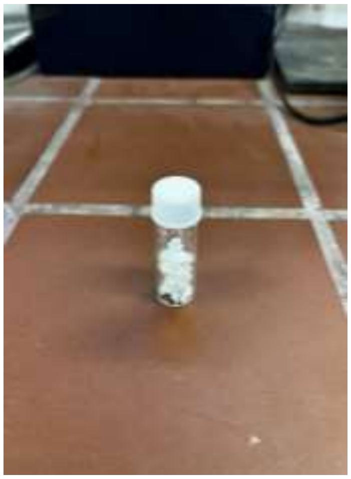

Polystyrene foam christmas ball (52 mg)

Yield of benzoic acid (59%)

Polystyrene Transparent Cutlery Set

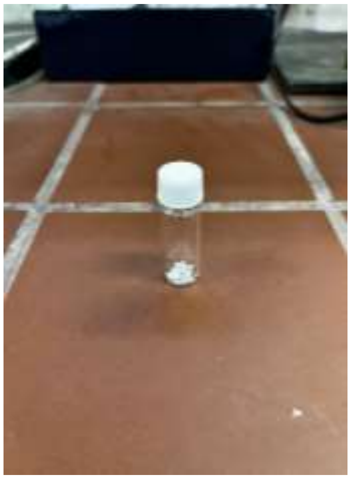

Polystyrene transparent cutlery set (52 mg)

Yield of benzoic acid (52%)

Polystyrene (MW: 90.000)

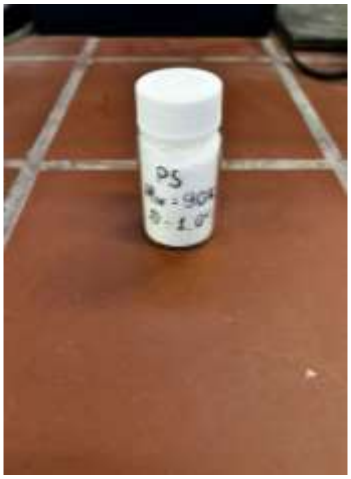

Polystyrene (MW: 90.000) (52 mg)

Yield of benzoic acid (47%)

Polystyrene (MW: 390.000)

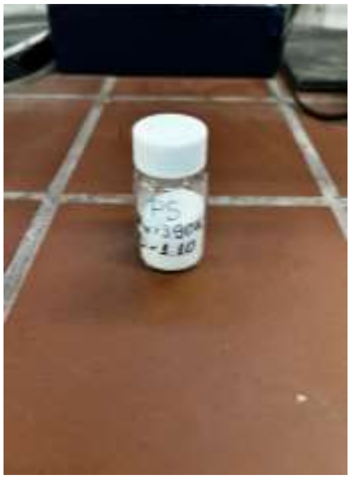

Polystyrene (MW: 390.000) (52 mg)

Yield of benzoic acid (51%)

Mixed Polystyrene Products Applications

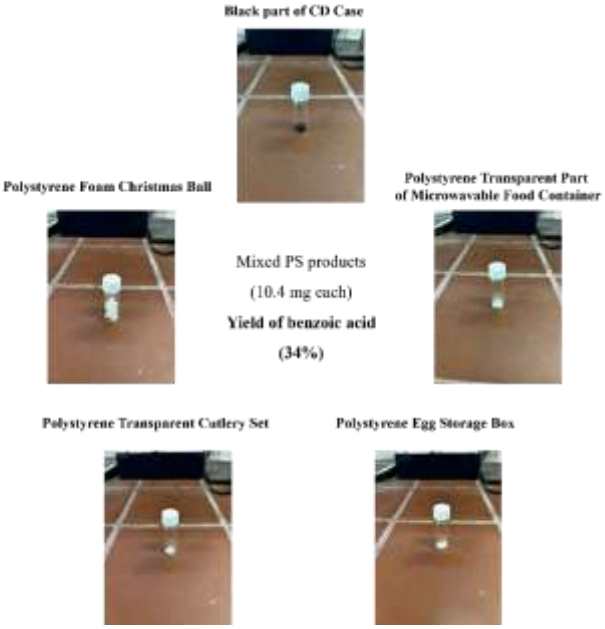

Figure S2. Application 1 of the photochemical protocol to 5 different PS products.

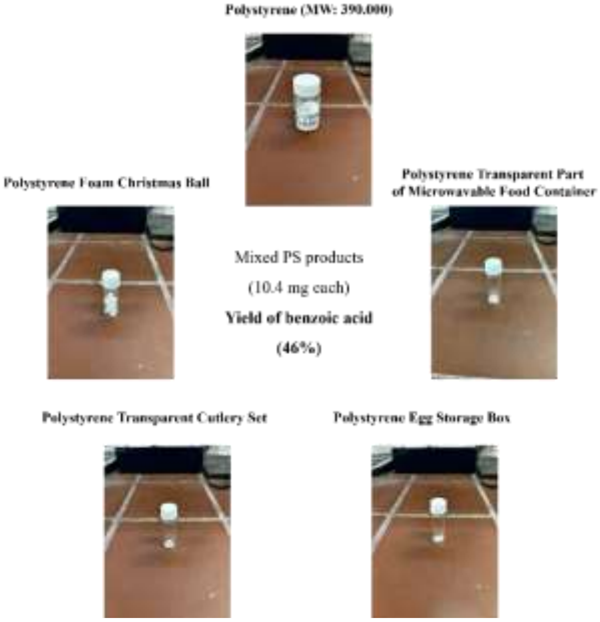

Figure S3. Application 2 of the photochemical protocol to 5 different PS products.

Large Scale Photochemical Aerobic Upcycling of Polystyrene

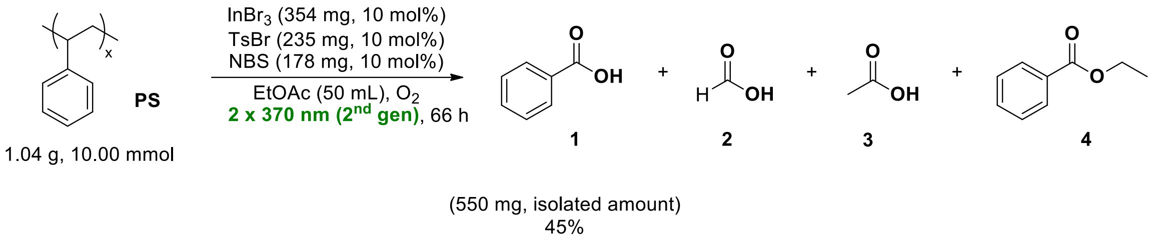

ratio between benzoic acid and ethyl benzoate based on ^{1}***H-NMR spectroscopy* (1:0.07)**

In a test tube containing polystyrene (1.04 g, 10.00 mmol based on the repeating unit, CAS Number: 9052-95-3, 1.00 equiv.), indium tribromide ($InBr_3$) (354 mg, 1.00 mmol, 0.10 equiv.), tosyl bromide (TsBr) (235 mg, 1.00 mmol, 0.10 equiv.), *N*-bromosuccinimide (NBS) (178 mg, 1.00 mmol, 0.10 equiv.) and EtOAc (50 mL) were added. The reaction mixture was sealed with a septum and parafilm and was placed in an ultrasonic bath for 30 minutes. Then, a first degassing was performed using vacuum and argon flow, followed by a second degassing using oxygen flow. The atmosphere in the test tube was maintained by attaching four balloons filled with oxygen. The reaction mixture was stirred and irradiated with two 2nd generation Kessil lamps at 370 nm for 66 hours. After reaction completion, the reaction mixture was diluted with CH_2Cl_2 (25 mL) and filtered under vacuum to remove the insoluble residues (150 mg) corresponding to the starting material or lower molecular weight polymeric/oligomeric species. The filtrate was then concentrated *in vacuo*. Then, CH_2Cl_2 (50 mL) and aq. NaOH 1N (50 mL) were added to the flask and the crude reaction mixture was stirred for 15 min. The layers were separated in a separatory funnel. The aqueous phase was then acidified with concentrated HCl until pH = 1. Then, the aqueous layer was extracted with CH_2Cl_2 (3 x 50 mL) and the combined organic layers were dried over Na_2SO_4 and concentrated *in vacuo*, affording the desired benzoic acid (**1**) in 45% yield (550 mg, 4.50 mmol). The first organic layer (CH_2Cl_2) was also concentrated revealing the existence of ethyl benzoate (**4**) in small amounts. Formic acid (**2**) and acetic acid (**3**) were also detected in the NMR spectrum of the crude reaction mixture.

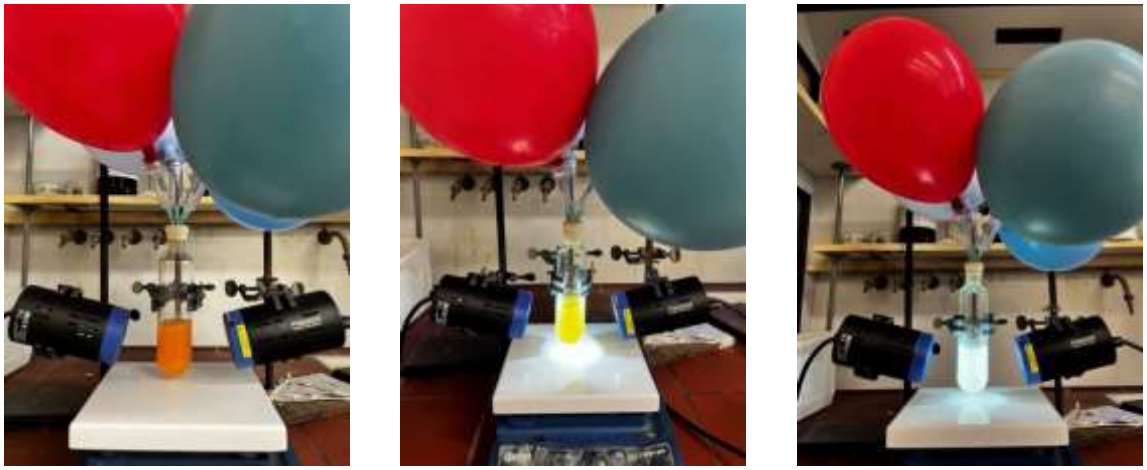

Figure S4. A: Reaction setup for the large-scale experiment before irradiation, **B**: Beginning of the reaction, **C**: Progress of the reaction – discoloration of the crude reaction mixture.

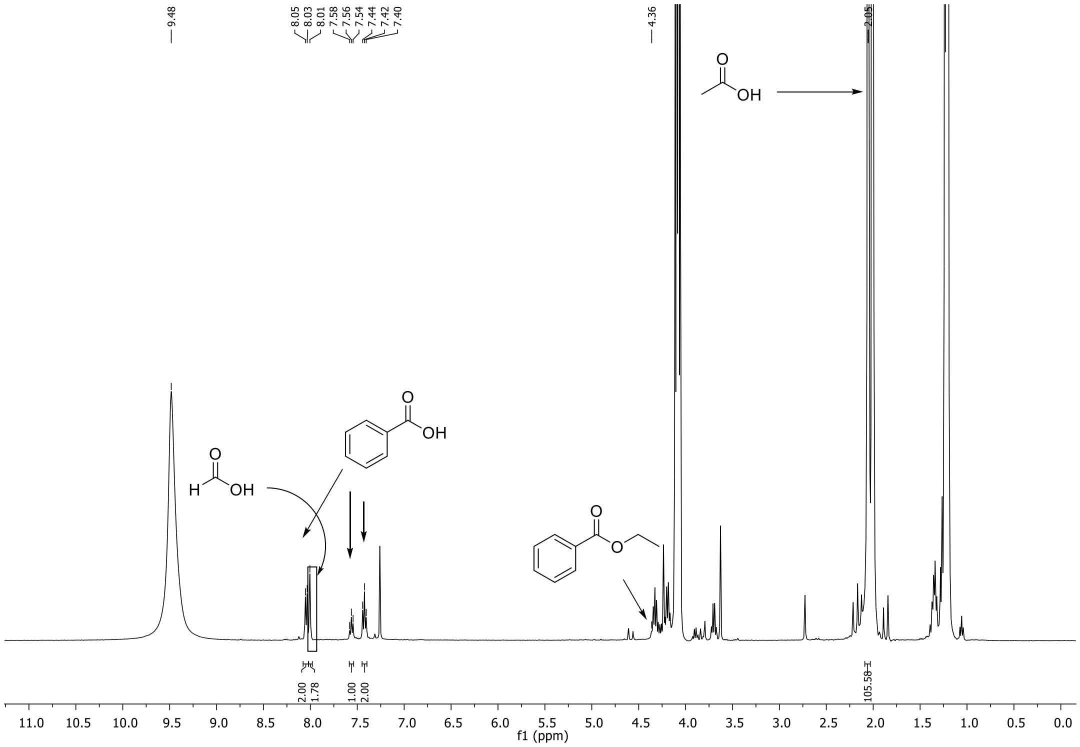

^{1}H NMR spectrum (400 MHz, $CDCl_3$) of the crude reaction mixture from the large-scale reaction before filtration and solvent evaporation: Identifying the presence of benzoic acid (1), formic acid (2), and acetic acid (3) as the major products and ethyl benzoate (4) as minor product from the photochemical aerobic upcycling process.

Progress of the Large Scale Reaction *via* ^{1}H-NMR

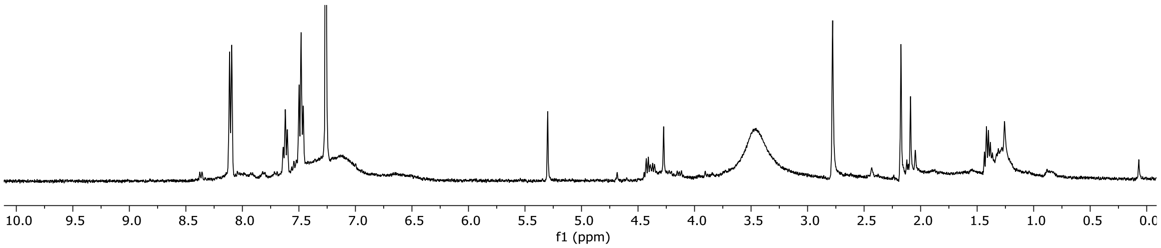

^{1}H-NMR of the large scale reaction after 18 h

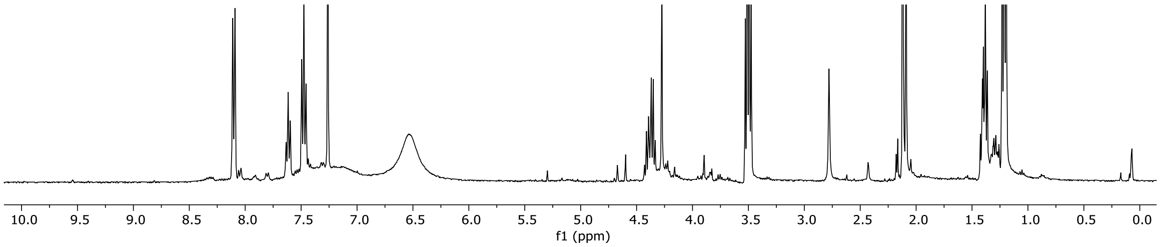

^{1}H-NMR of the large scale reaction after 24 h

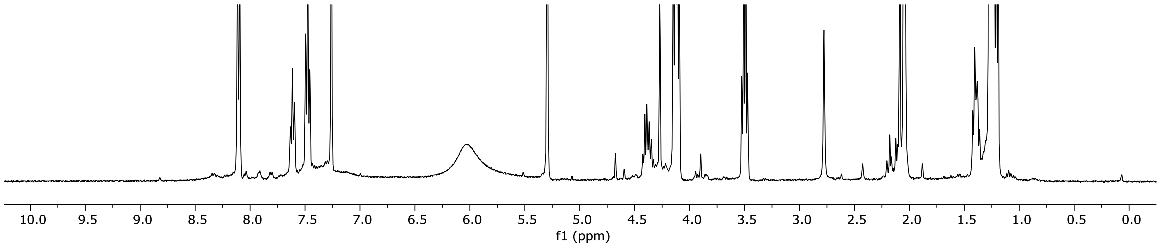

^{1}H-NMR of the large scale reaction after 42 h

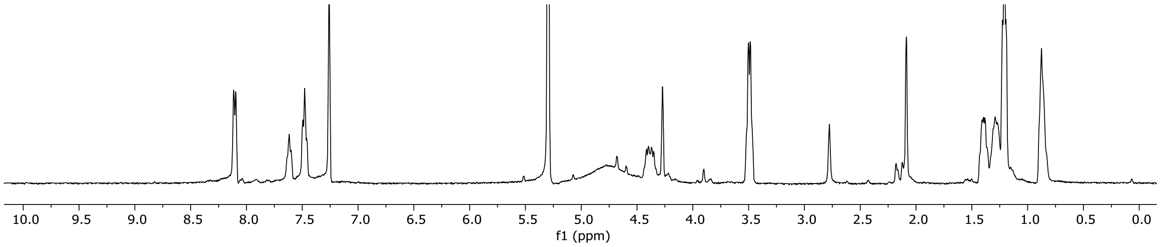

^{1}H-NMR of the large scale reaction after 48 h

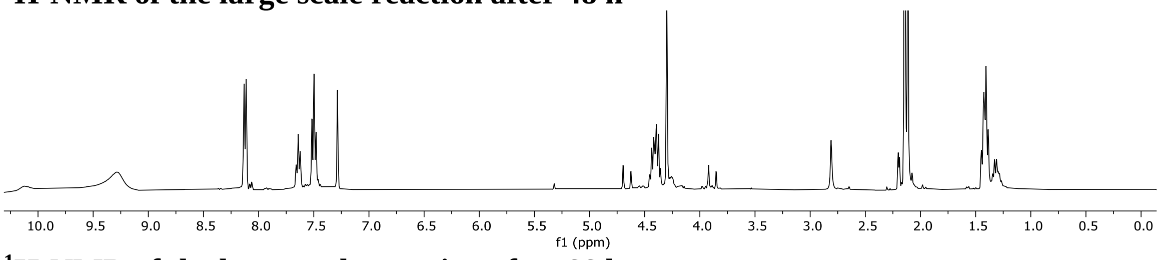

^{1}H-NMR of the large scale reaction after 66 h

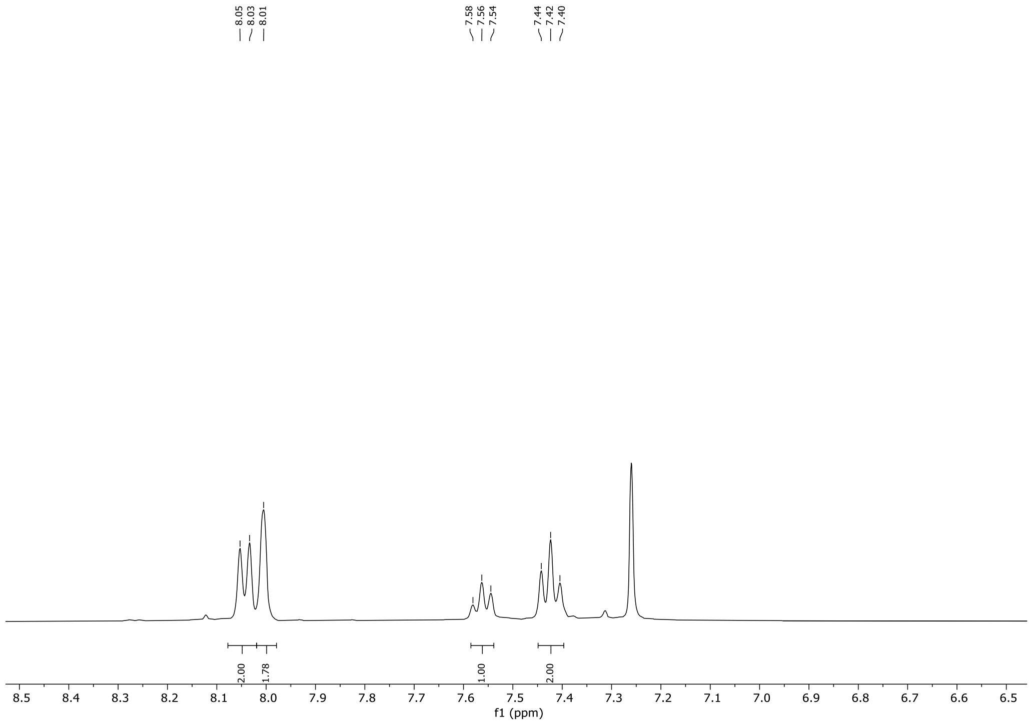

Zoom in ^{1}H NMR (400 MHz, $CDCl_3$) spectrum from 8.50 to 6.50 ppm of the large-scale reaction, presenting benzoic acid (1) and formic acid (2).

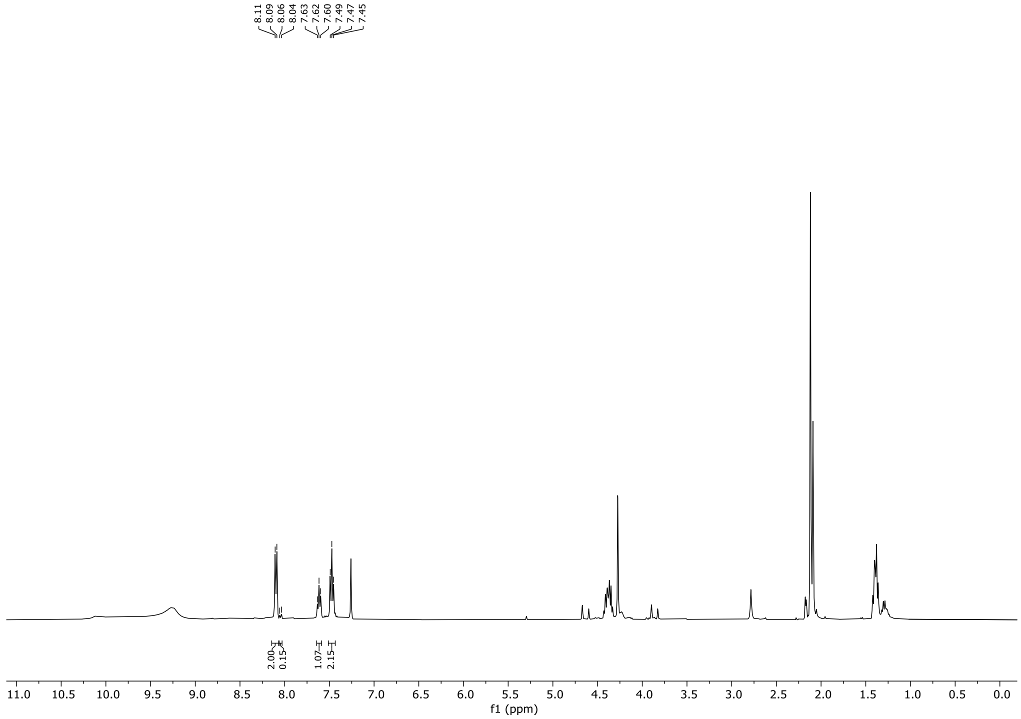

^{1}H NMR spectrum (400 MHz, $CDCl_3$) of the crude reaction mixture from the large-scale reaction after filtration and solvent evaporation showing benzoic acid (1) as the major product and ethyl benzoate (4) as a minor byproduct.

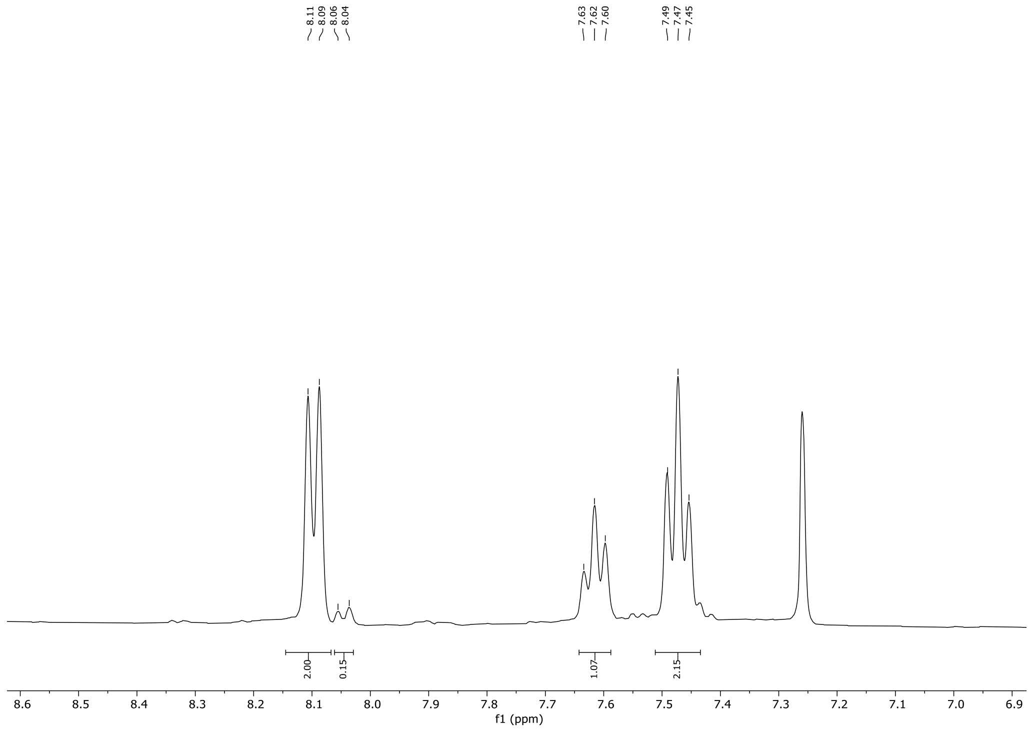

Zoom in ^{1}H NMR (400 MHz, CDCl$_3$) spectrum from 8.60 to 6.90 ppm of the large-scale reaction, presenting benzoic acid (1) and ethyl benzoate (4).

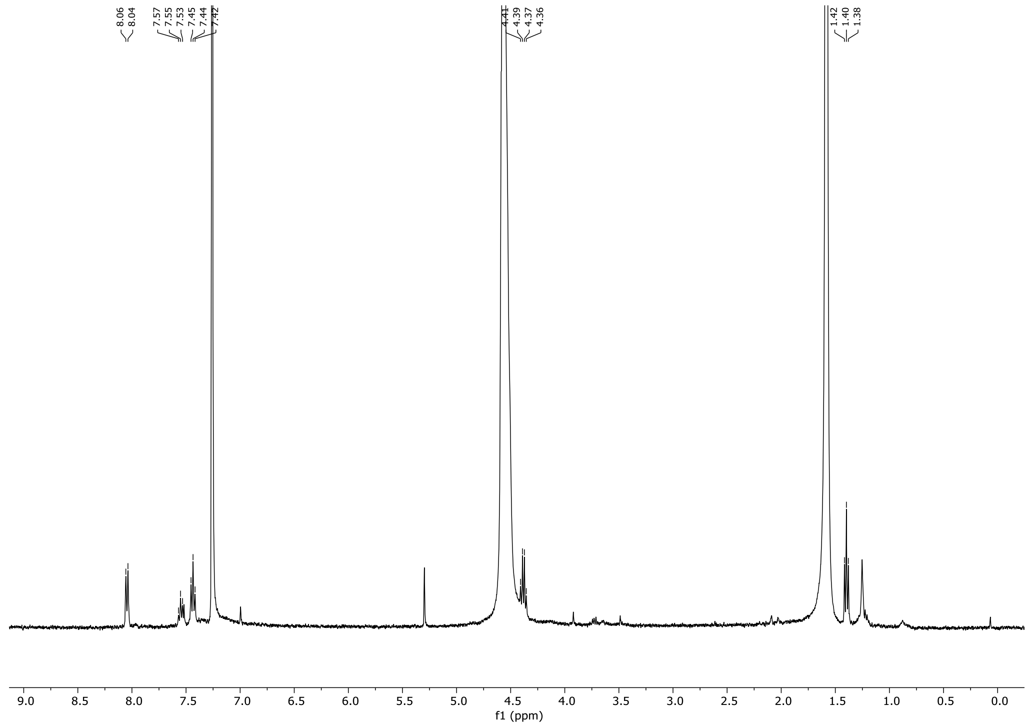

^{1}H NMR spectrum (400 MHz, CDCl$_3$) of the first organic layer after the addition of NaOH 1N, indicating the presence of ethyl benzoate (4) as a reaction byproduct.

Synthesis of Butane-1,3-diyldibenzene (5) for Mechanistic Studies

2,4-Diphenylbutan-2-ol (S1)[2]

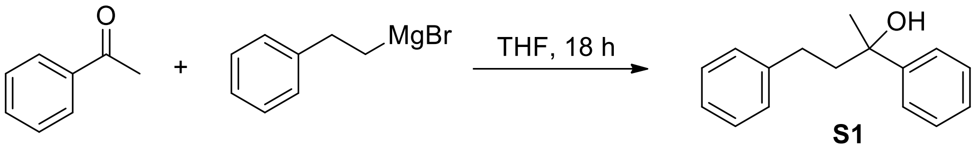

A flame-dried round-bottom flask was charged with magnesium turnings (209 mg, 8.60 mmol, 1.60 equiv.) and a small crystal of iodine under an argon atmosphere. Dry THF (5 mL) was added, followed by the dropwise addition of (2-bromoethyl)benzene (1.17 mL, 8.60 mmol, 1.60 equiv.). The mixture was stirred for 30 minutes, after which acetophenone (0.63 mL, 5.40 mmol, 1.00 equiv.) diluted in dry THF (5 mL) was added dropwise. Stirring was continued for 18 h at room temperature. The reaction was diluted with Et_2O (10 mL), quenched with aqueous NH_4Cl (5 mL), and further diluted with H_2O (10 mL). The layers were separated, and the aqueous phase was extracted with Et_2O (2 x 30 mL). The combined organic layers were washed with brine (20 mL), dried over Na_2SO_4, filtered, and concentrated under reduced pressure. The desired product **S1** was purified by column chromatography. Colourless oil; Yield 65%; **^{1}H NMR** (400 MHz, $CDCl_3$) δ: 7.52 (2H, d, J = 7.5 Hz, ArH), 7.41 (2H, t, J = 7.5 Hz, ArH), 7.34-7.24 (3H, m, ArH), 7.23-7.12 (3H, m, ArH), 2.72-2.61 (1H, m, PhCH*H*), 2.54-2.41 (1H, m, PhCH*H*), 2.24-2.09 (2H, m, CH_2), 1.81 (1H, br s, OH), 1.65 (3H, s, CH_3); **^{13}C NMR** ($CDCl_3$) δ: 147.5, 142.2, 128.4, 128.3, 128.2, 126.6, 125.7, 124.7, 74.7, 45.9, 30.5, 30.4; **MS (ESI)** *m/z* 227 $[M+H]^+$.

(*E*)-But-2-ene-1,3-diyldibenzene and but-3-ene-1,3-diyldibenzene (S2)[2]

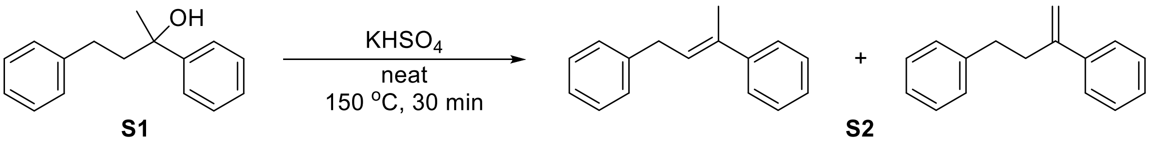

S1 (543 mg, 2.40 mmol, 1.00 equiv.) was added to a round bottom flask, charged with $KHSO_4$ (586 mg, 4.30 mmol, 1.80 equiv.) and heated neat at 150 °C for 30 min. Upon

reaction completion, the reaction mixture was diluted with CH_2Cl_2 (20 mL) and filtered through celite. The filtrate was evaporated *in vacuo* and the crude product mixture was utilized directly without further purification to the next step (51% yield).

Butane-1,3-diyldibenzene (5)[2]

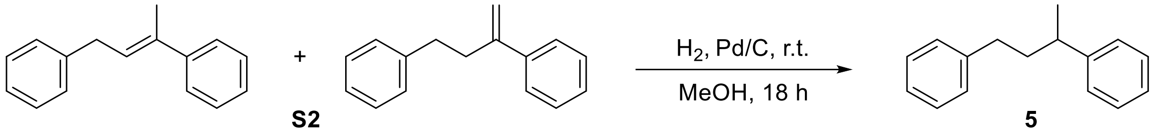

A round-bottom flask containing the product mixture **S2** (208 mg, 1.00 mmol, 1.00 equiv.) was charged with methanol (10 mL) and a catalytic amount of Pd/C (10 % w/w, 30 mg). The reaction mixture was stirred at room temperature for 18 h under a hydrogen atmosphere. Upon completion, it was diluted with methanol (20 mL) and filtered through celite. The filtrate was concentrated under reduced pressure, and the crude reaction mixture was purified by column chromatography affording **5** in 99% yield. Colourless oil; ^{1}**H NMR** (400 MHz, $CDCl_3$) δ: 7.56-7.23 (10H, m, ArH), 2.95-2.82 (1H, m, PhCH), 2.75-2.62 (2H, m, $PhCH_2$), 2.19-2.01 (2H, m, CH_2), 1.47-1.45 (3H, d, J = 7.0 Hz, CH_3); ^{13}**C NMR** ($CDCl_3$) δ: 147.2, 142.5, 128.4, 128.3, 128.2, 127.0, 125.9, 125.6, 39.9, 39.5, 33.9, 22.5; **MS (ESI)** *m/z* 211 $[M+H]^+$.

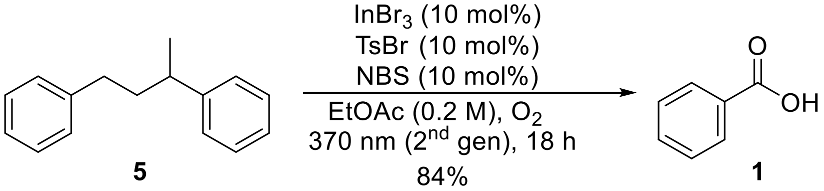

In a test tube containing butane-1,3-diyldibenzene (**5**) (63 mg, 0.30 mmol, 1.00 equiv.), indium tribromide ($InBr_3$) (11 mg, 0.03 mmol, 0.10 equiv.), tosyl bromide (TsBr) (7 mg, 0.03 mmol, 0.10 equiv.), *N*-bromosuccinimide (NBS) (5 mg, 0.03 mmol, 0.10 equiv.) and EtOAc (1.5 mL) were added. The reaction mixture was sealed with a septum and parafilm and was placed in an ultrasonic bath for 10 minutes. Then, a first degassing was performed using vacuum and argon flow, followed by a second degassing using oxygen

flow. The atmosphere in the test tube was maintained by attaching two balloons filled with oxygen. The reaction mixture was stirred and irradiated with a 2nd generation Kessil lamp at 370 nm for 18 hours. After reaction completion, the reaction mixture was diluted with CH_2Cl_2 (2.5 mL) and filtered under vacuum to remove the insoluble residues corresponding to the starting material or lower molecular weight polymeric/oligomeric species. The filtrate was then concentrated *in vacuo.* Then, CH_2Cl_2 (5 mL) and aq. NaOH 1N (2.5 mL) were added to the flask and the crude reaction mixture was stirred for 10 min. The layers were separated in a separatory funnel. The aqueous phase was then acidified with concentrated HCl until pH = 1. Then, the aqueous layer was extracted with CH_2Cl_2 (3 x 5 mL) and the combined organic layers were dried over Na_2SO_4 and concentrated *in vacuo*, affording the desired benzoic acid (**1**) in 84% yield.

Direct Infusion-High Resolution Mass Spectrometry (DI-HRMS) Mechanistic Studies

Instrumentation

High Resolution Mass Spectra were recorded with a Q-TOF (Time of Flight Mass Spectrometer) Bruker Maxis Impact with electrospray ionization (ESI) source. N_2 was used as collision gas and positive ionization mode was used for all MS experiments. The data acquisition was carried out with Data Analysis from Bruker Daltonics (version 4.1). Acetonitrile LC-MS gradient was obtained from Carlo Erba Reagents (Chaussée du Vexin, France). Source conditions: End plate offset 500V, Capillary 4500V, Nebulizer 0.4 bar, dry gas 4.0 L/min, dry temperature 180 ^{o}C and Quadrupole conditions: Ion energy 5 eV, Collision energy 10 eV, Transfer time 143 μs, Collision ion RF 3500 vpp, Pre pulse storage 1μs.

HRMS studies were performed with an ESI source under positive and negative ionization mode. The annotation of the intermediates was based on the exact mass high accuracy (mass error lower than 5 ppm).

Products of the photochemical oxidation of butane-1,3-diyldibenzene (5) under 370 nm 2nd generation Kessil lamp irradiation

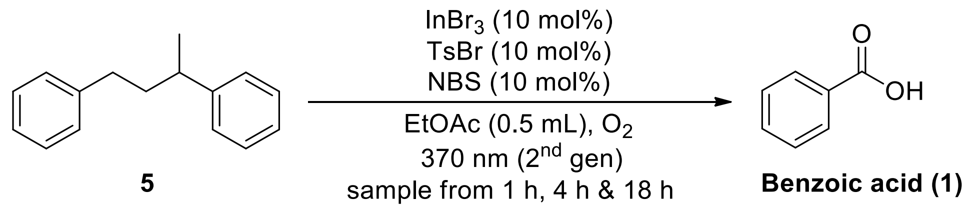

The products of the oxidation of the photochemical cleavage of butane-1,3-diyldibenzene (**5**), upon irradiation with 370 nm 2nd generation Kessil lamp, were monitored for 18 hours by DI-HRMS. Butane-1,3-diyldibenzene (**5**) (21 mg, 0.10 mmol), indium

tribromide ($InBr_3$) (3.6 mg, 0.01 mmol, 0.10 equiv.), tosyl bromide (TsBr) (2.4 mg, 0.01 mmol, 0.10 equiv.) and *N*-bromosuccinimide (NBS) (1.8 mg, 0.01 mmol, 0.10 equiv.) were dissolved in EtOAc (0.5 mL). The reaction mixture was sealed with a septum and parafilm. Then, a first degassing was performed using vacuum and argon flow, followed by a second degassing using oxygen flow. The atmosphere in the test tube was maintained by attaching two balloons filled with oxygen. The reaction mixture was stirred and irradiated with a 2nd generation Kessil lamp at 370 nm for 18 hours. At the specific time of study (1 h, 4 h and 18 h), a sample of the reaction mixture (10 μL) was first diluted with 990 μL methanol and 100 μL of that sample were further diluted with 900 μL of methanol. Finally, 100 μL were injected for DI-HRMS analysis.

A suspect analysis approach revealed the formation of peaks corresponding to butane-1,3-diyldibenzene (**5**), intermediates containing hydroxy (**21** or **22**) or peroxy (**12** or **13**) groups, as well as oxygenated compound **11** and benzoic acid (**1**). Additionally, we were able to identify **3, 4, 14-20** and **23-27**.

Intermediates observed after 1 h irradiation

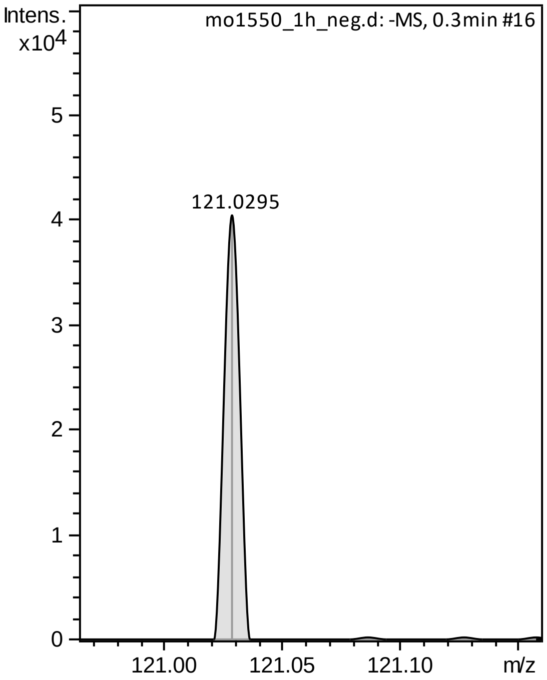

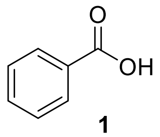

1

Exact Mass Calculated for $[M-H]^-$ ($C_7H_5O_2^-$)	Exact Mass Found for $[M-H]^-$($C_7H_5O_2^-$)
121.0295	121.0295

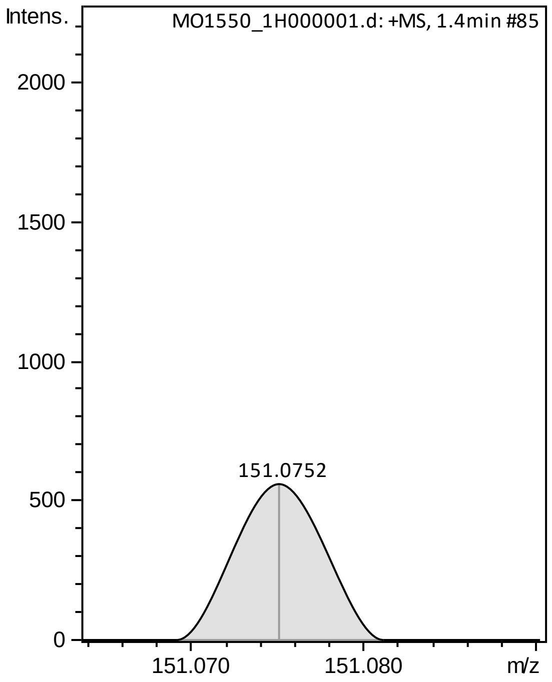

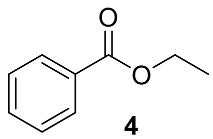

4

Exact Mass Calculated for $[M+H]^+$ ($C_9H_{11}O_2^+$)	Exact Mass Found for $[M+H]^+$ ($C_9H_{11}O_2^+$)
151.0754	151.0752

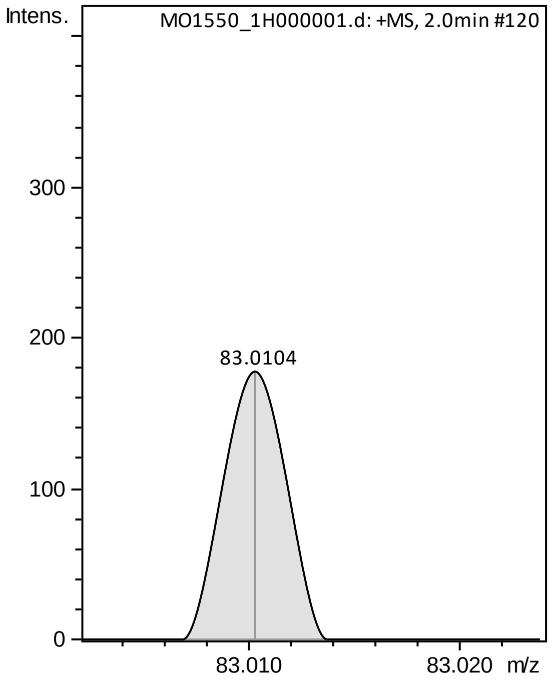

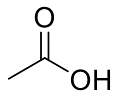

3

Exact Mass Calculated for $[M+Na]^+$ ($C_2H_4NaO_2^+$)	Exact Mass Found for $[M+Na]^+$ ($C_2H_4NaO_2^+$)
83.0104	83.0104

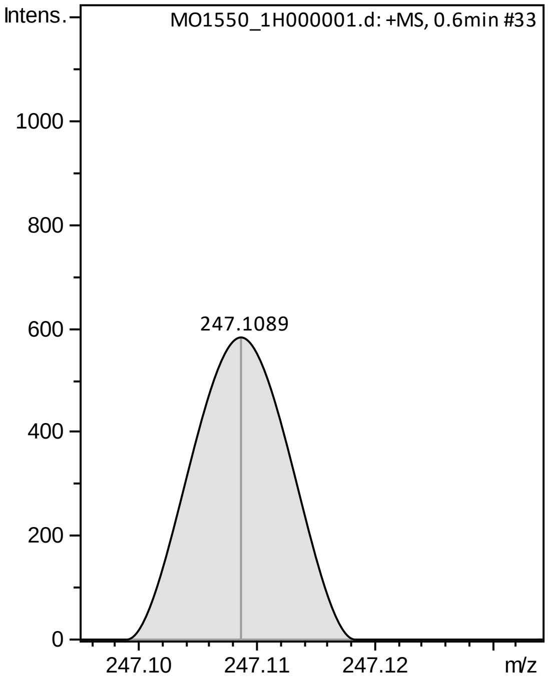

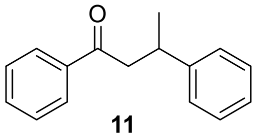

11

Exact Mass Calculated for [M+Na]+ ($C_{16}H_{16}NaO^+$)	Exact Mass Found for [M+Na]+ ($C_{16}H_{16}NaO^+$)
247.1093	247.1089

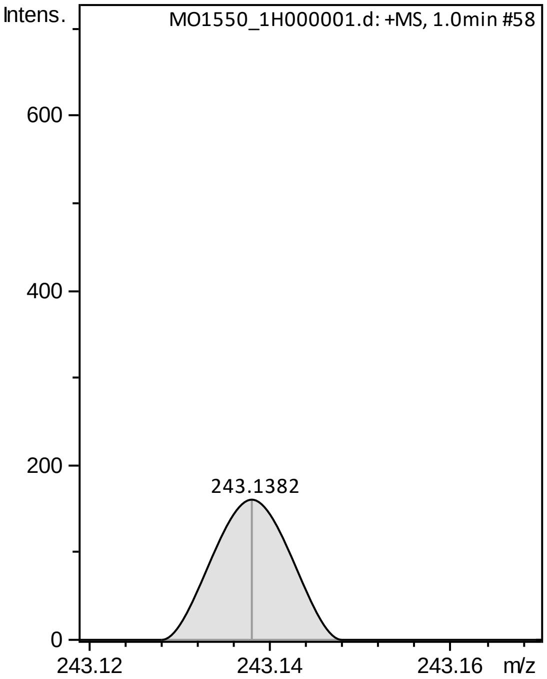

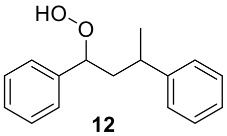

12

or

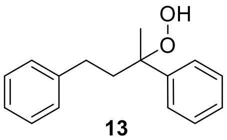

13

Exact Mass Calculated for [M+H]+ ($C_{16}H_{19}O_2^+$)	Exact Mass Found for [M+H]+ ($C_{16}H_{19}O_2^+$)
243.1380	243.1382

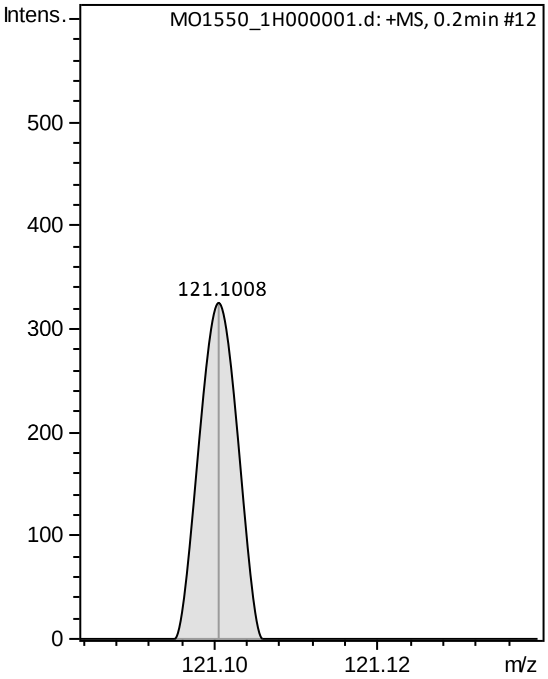

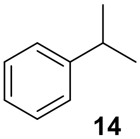

14

Exact Mass Calculated for $[M+H]^+$ ($C_9H_{13}^+$)	Exact Mass Found for $[M+H]^+$ ($C_9H_{13}^+$)
121.1012	121.1008

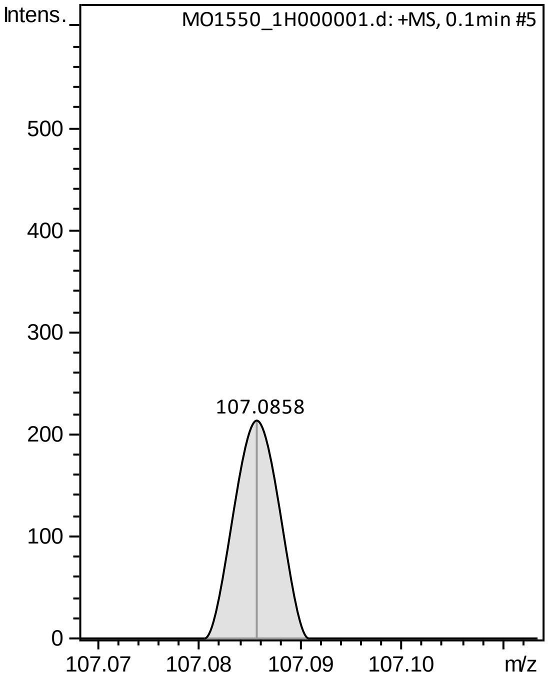

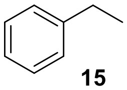

15

Exact Mass Calculated for $[M+H]^+$ ($C_8H_{11}^+$)	Exact Mass Found for $[M+H]^+$ ($C_8H_{11}^+$)
107.0855	107.0858

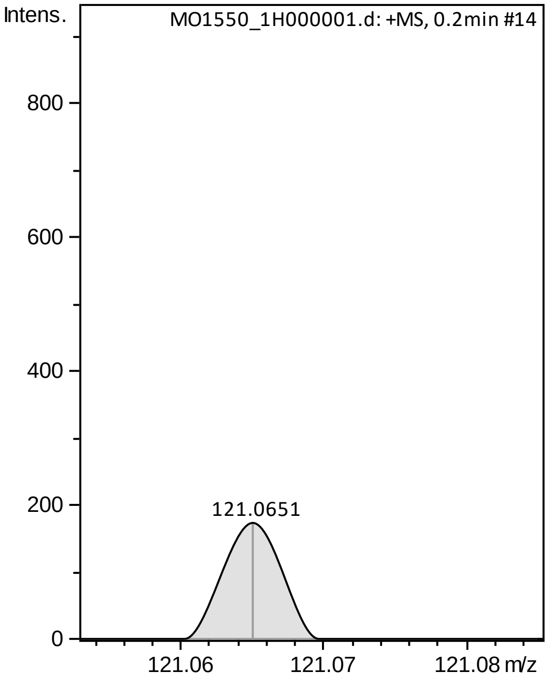

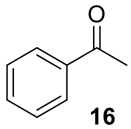

16

Exact Mass Calculated for [M+H]$^+$ ($C_8H_9O^+$)	Exact Mass Found for [M+H]$^+$ ($C_8H_9O^+$)
121.0648	121.0651

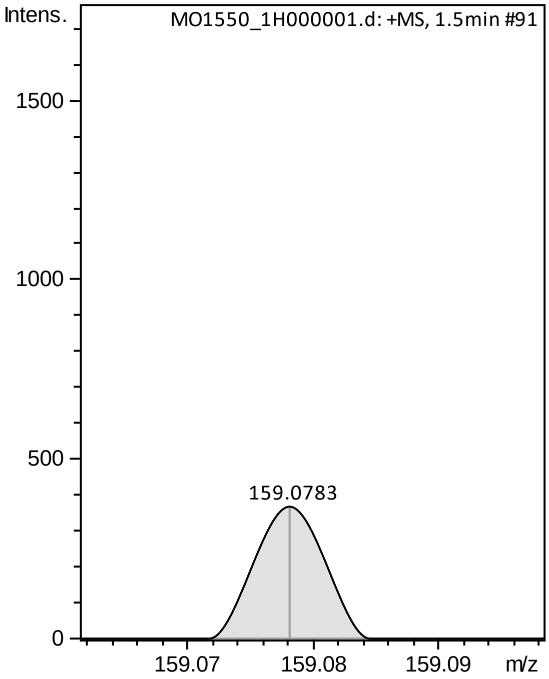

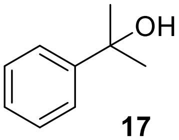

17

Exact Mass Calculated for [M+Na]$^+$ ($C_9H_{12}NaO^+$)	Exact Mass Found for [M+Na]$^+$ ($C_9H_{12}NaO^+$)
159.0780	159.0783

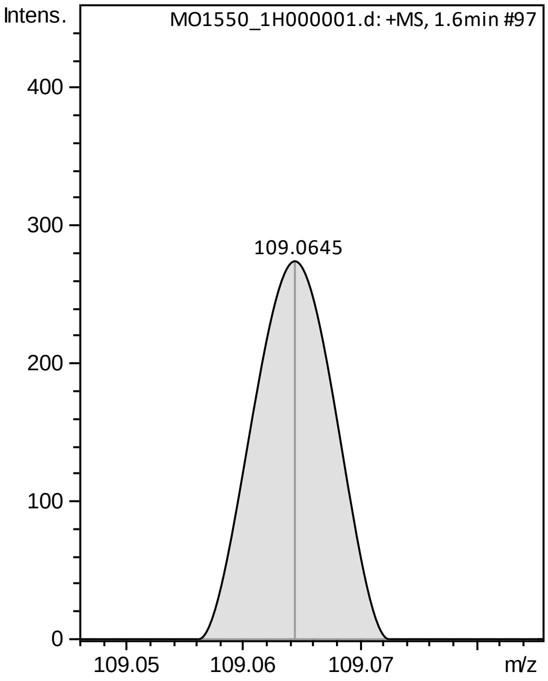

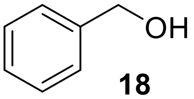

18

Exact Mass Calculated for $[M+H]^+$ ($C_7H_9O^+$)	Exact Mass Found for $[M+H]^+$ ($C_7H_9O^+$)
109.0648	109.0645

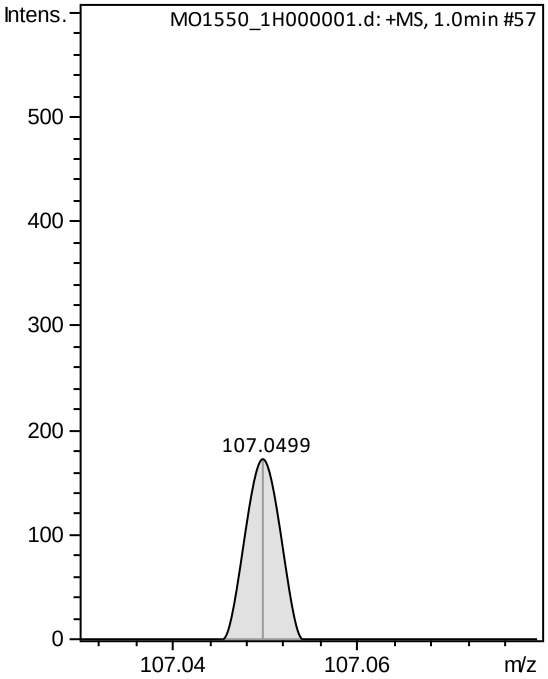

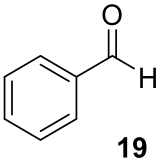

19

Exact Mass Calculated for $[M+H]^+$ ($C_7H_7O^+$)	Exact Mass Found for $[M+H]^+$ ($C_7H_7O^+$)
107.0491	107.0499

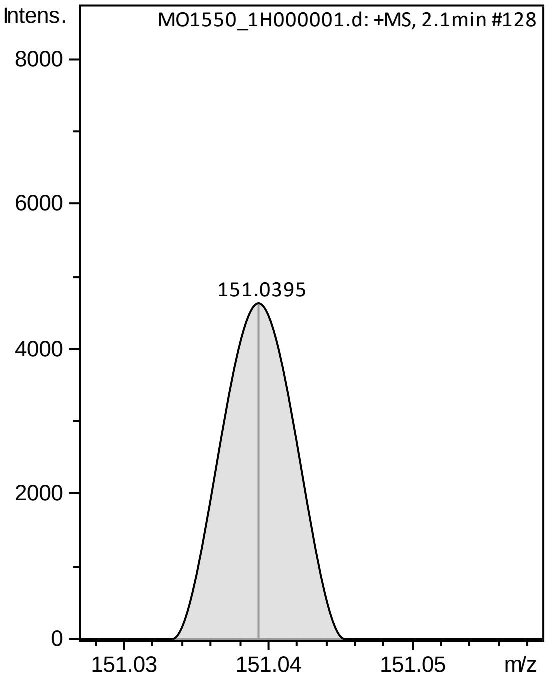

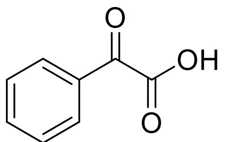

20

Exact Mass Calculated for $[M+H]^+$ ($C_8H_7O_3^+$)	Exact Mass Found for $[M+H]^+$ ($C_8H_7O_3^+$)
151.0390	151.0395

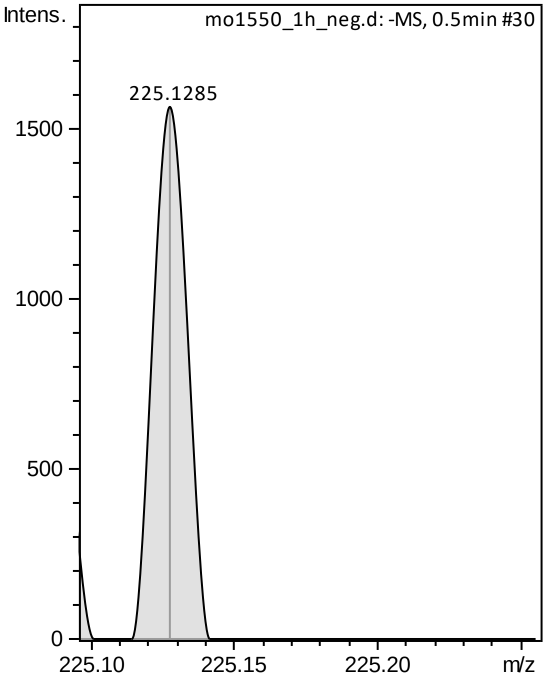

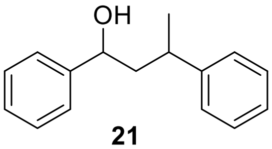

or

21

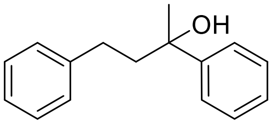

22

Exact Mass Calculated for $[M-H]^-$ ($C_{16}H_{17}O^-$)	Exact Mass Found for $[M-H]^-$ ($C_{16}H_{17}O^-$)
225.1285	225.1285

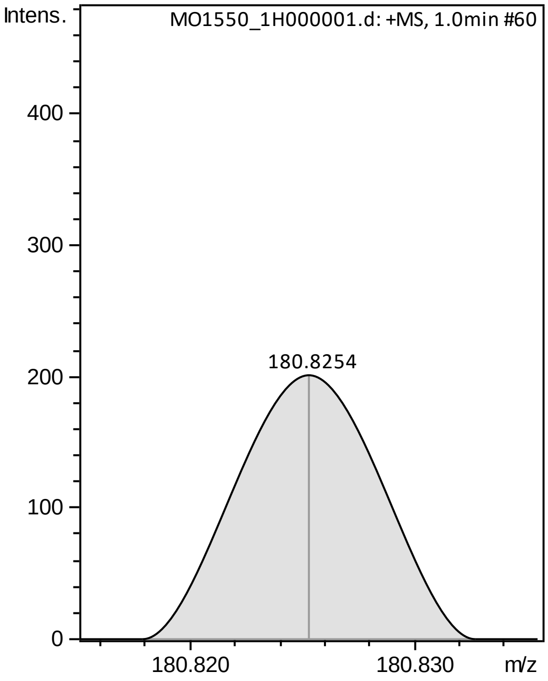

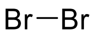

23

Exact Mass Calculated for $[M+Na]^+$ (Br_2Na^+)	Exact Mass Found for $[M+Na]^+$ (Br_2Na^+)
180.8259	180.8254

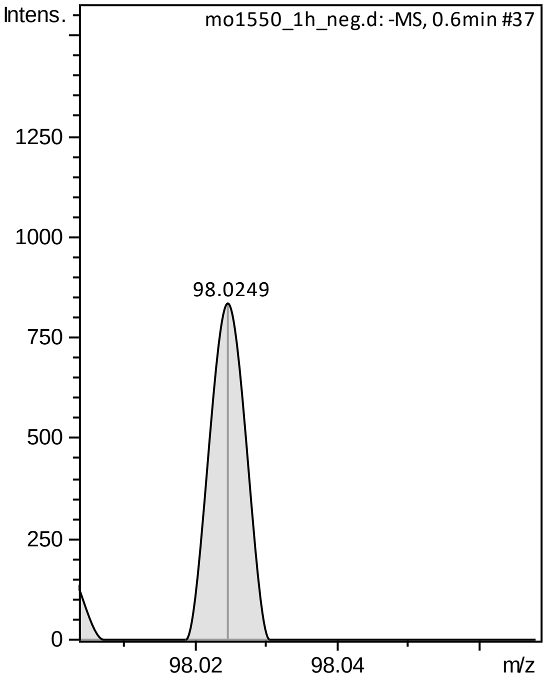

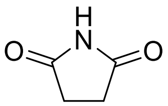

24

Exact Mass Calculated for $[M-H]^-$ ($C_4H_4NO_2^-$)	Exact Mass Found for $[M-H]^-$ ($C_4H_4NO_2^-$)
98.0248	98.0249

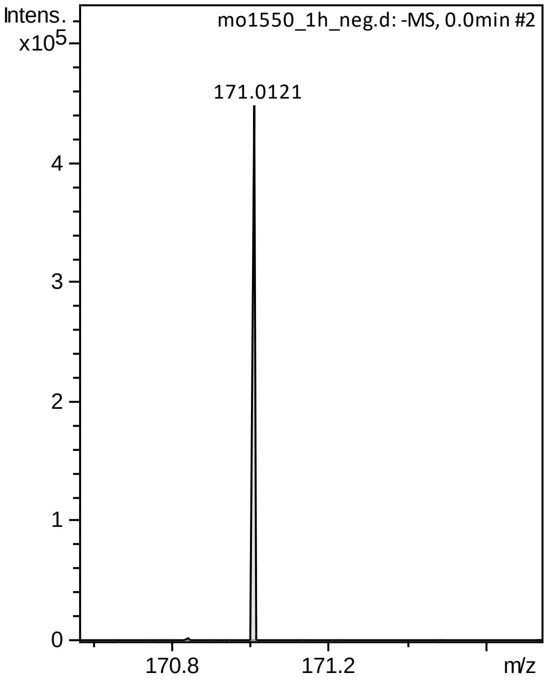

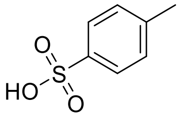

25

Exact Mass Calculated for $[M-H]^-$ ($C_7H_7O_3S^-$)	Exact Mass Found for $[M-H]^-$ ($C_7H_7O_3S^-$)
171.0121	171.0121

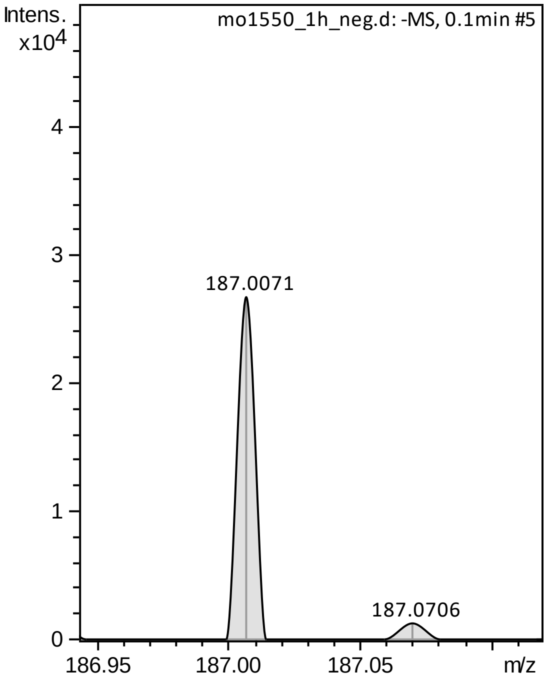

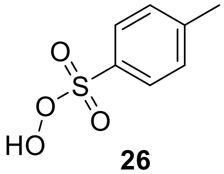

26

Exact Mass Calculated for $[M-H]^-$ ($C_7H_7O_4S^-$)	Exact Mass Found for $[M-H]^-$ ($C_7H_7O_4S^-$)
187.0071	187.0071

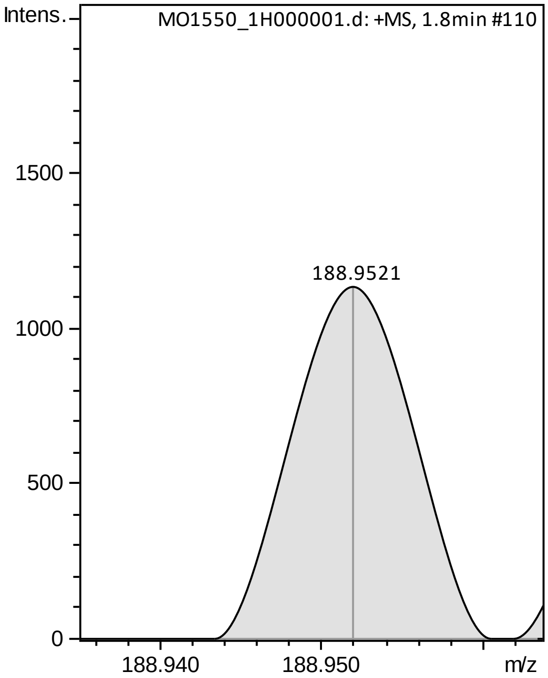

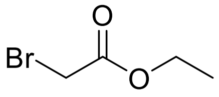

27

Exact Mass Calculated for [M+Na]$^+$ ($C_4H_7BrNaO_2^+$)	Exact Mass Found for [M+Na]$^+$ ($C_4H_7BrNaO_2^+$)
188.9522	188.9521

Intermediates observed after 4 h irradiation

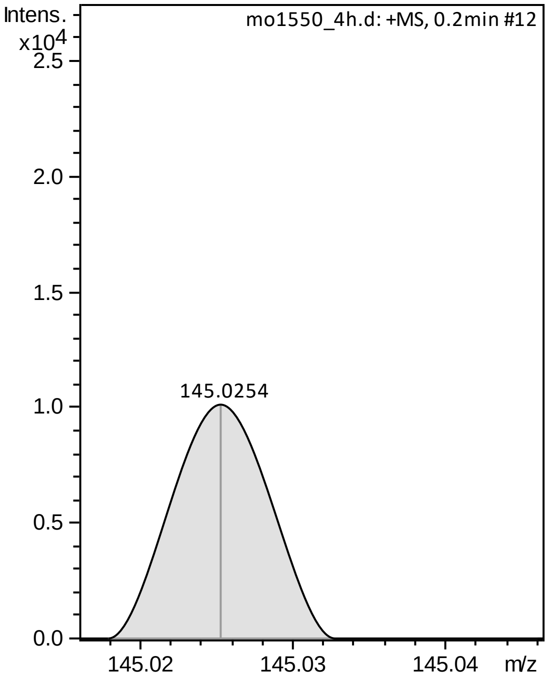

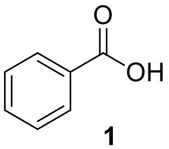

1

Exact Mass Calculated for [M+Na]$^+$ ($C_7H_6NaO_2^+$)	Exact Mass Found for [M+Na]$^+$ ($C_7H_6NaO_2^+$)
145.0260	145.0254

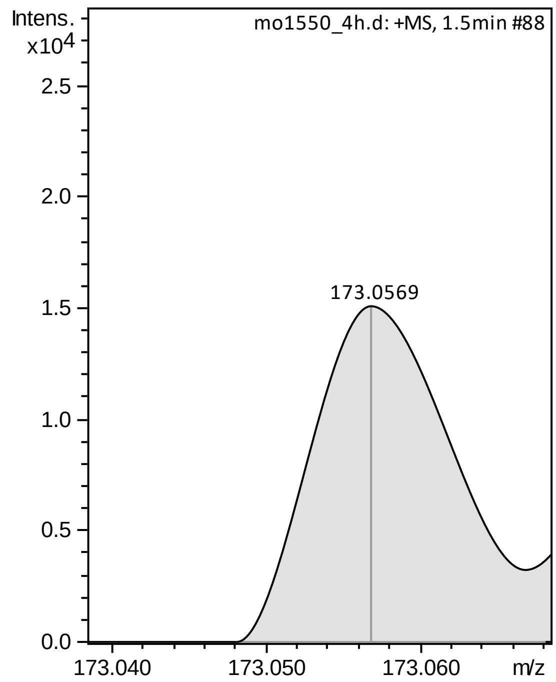

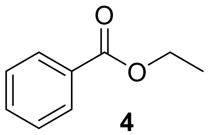

4

Exact Mass Calculated for $[M+Na]^+$ ($C_9H_{10}NaO_2^+$)	Exact Mass Found for $[M+Na]^+$ ($C_9H_{10}NaO_2^+$)
173.0573	173.0569

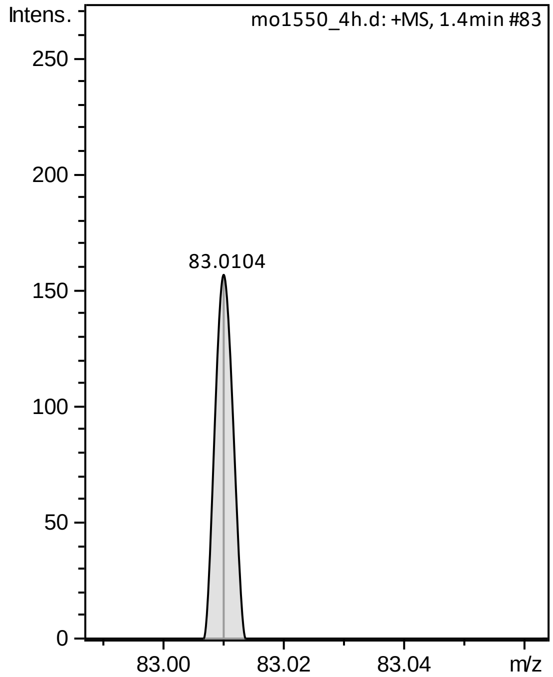

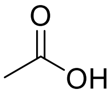

3

Exact Mass Calculated for $[M+Na]^+$ ($C_2H_4NaO_2^+$)	Exact Mass Found for $[M+Na]^+$ ($C_2H_4NaO_2^+$)
83.0104	83.0104

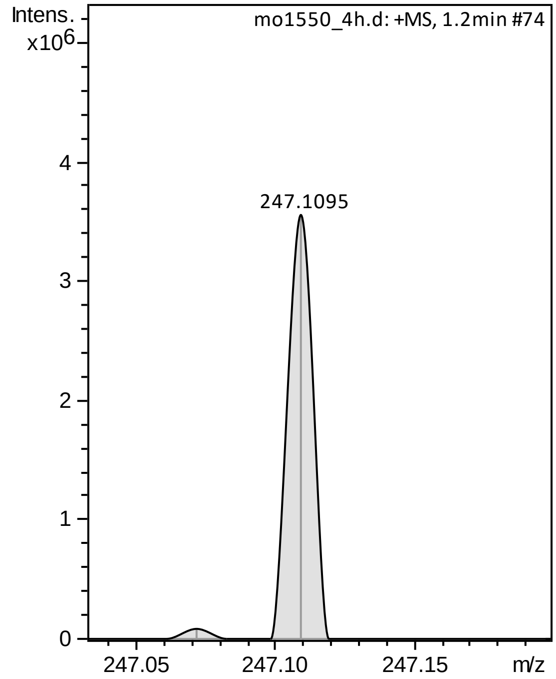

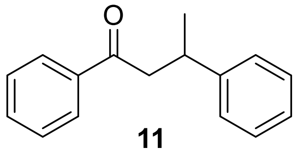

11

Exact Mass Calculated for $[M+Na]^+$ ($C_{16}H_{16}NaO^+$)	Exact Mass Found for $[M+Na]^+$ ($C_{16}H_{16}NaO^+$)
247.1093	247.1095

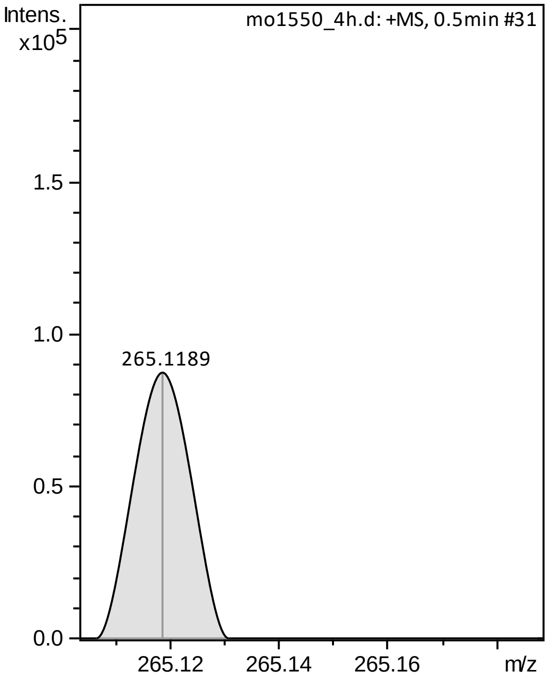

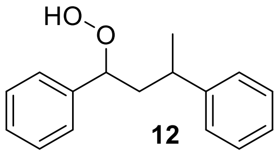

12

or

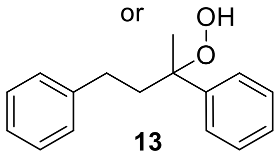

13

Exact Mass Calculated for $[M+Na]^+$ ($C_{16}H_{18}NaO_2^+$)	Exact Mass Found for $[M+Na]^+$ ($C_{16}H_{18}NaO_2^+$)
265.1199	265.1189

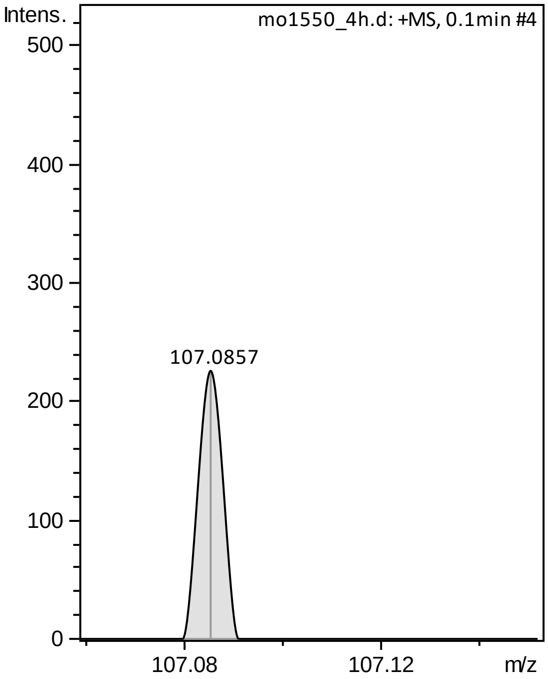

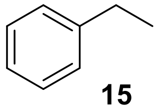

15

Exact Mass Calculated for $[M+H]^+$ ($C_8H_{11}^+$)	Exact Mass Found for $[M+H]^+$ ($C_8H_{11}^+$)
107.0855	107.0857

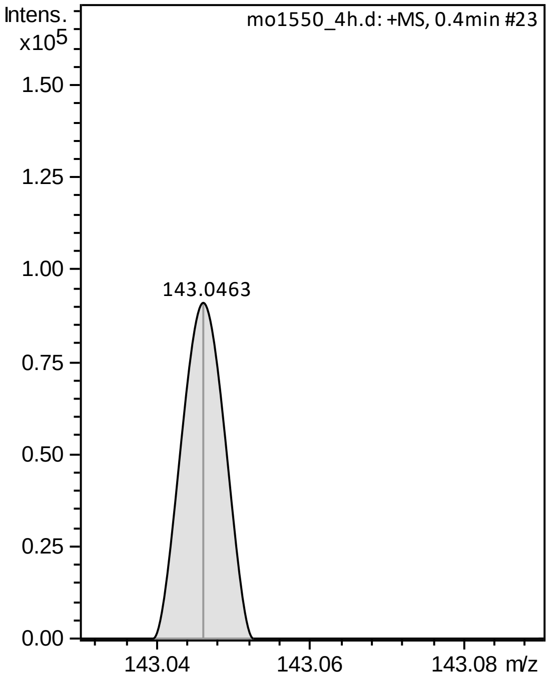

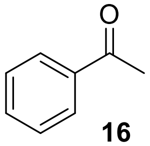

16

Exact Mass Calculated for $[M+Na]^+$ ($C_8H_8NaO^+$)	Exact Mass Found for $[M+Na]^+$ ($C_8H_8NaO^+$)
143.0467	143.0463

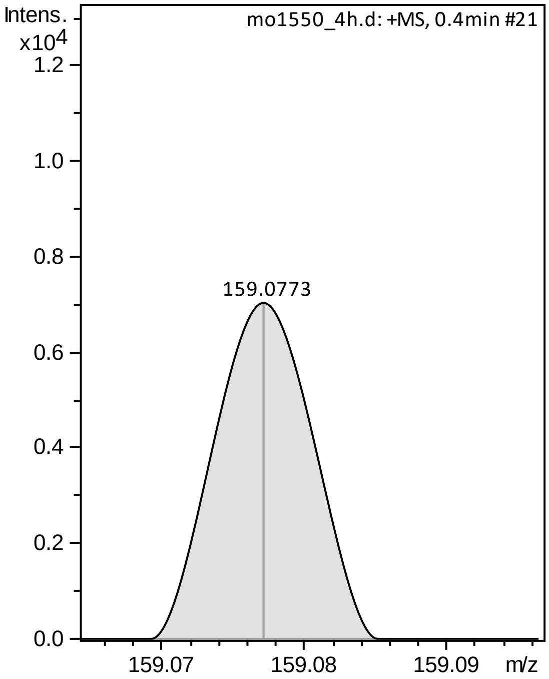

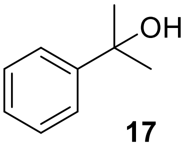

17

Exact Mass Calculated for $[M+Na]^+$ ($C_9H_{12}NaO^+$)	Exact Mass Found for $[M+Na]^+$ ($C_9H_{12}NaO^+$)
159.0780	159.0773

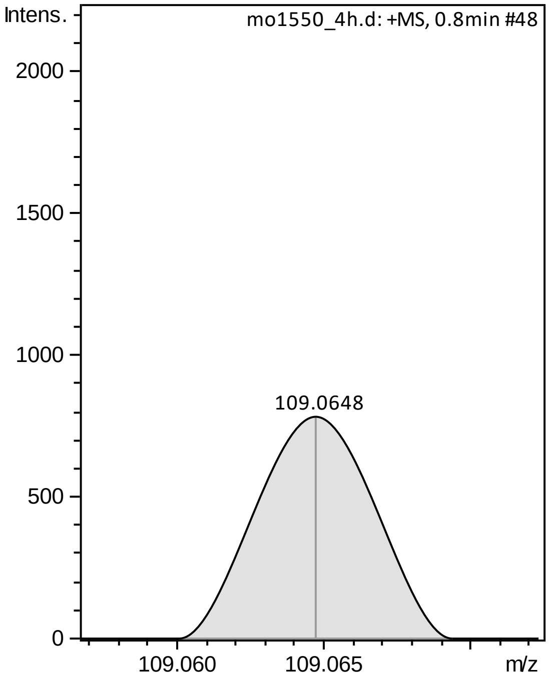

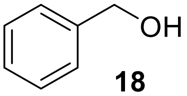

18

Exact Mass Calculated for $[M+H]^+$ ($C_7H_9O^+$)	Exact Mass Found for $[M+H]^+$ ($C_7H_9O^+$)
109.0648	109.0648

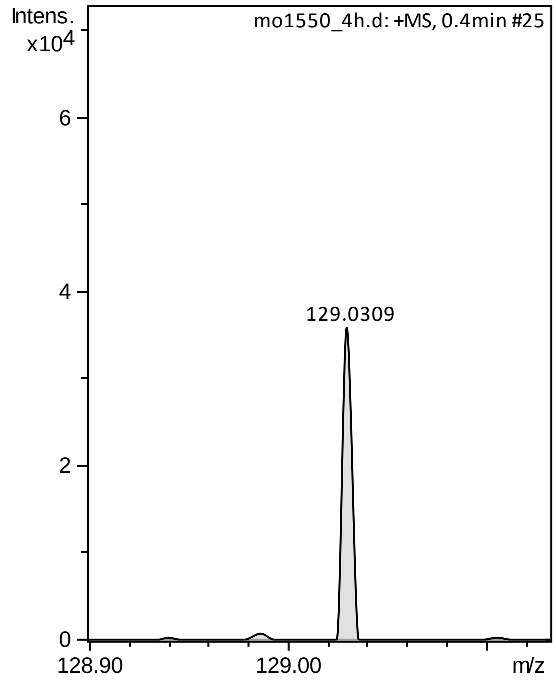

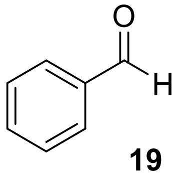

19

Exact Mass Calculated for $[M+Na]^+$ ($C_7H_6NaO^+$)	Exact Mass Found for $[M+Na]^+$ ($C_7H_6NaO^+$)
129.0311	129.0309

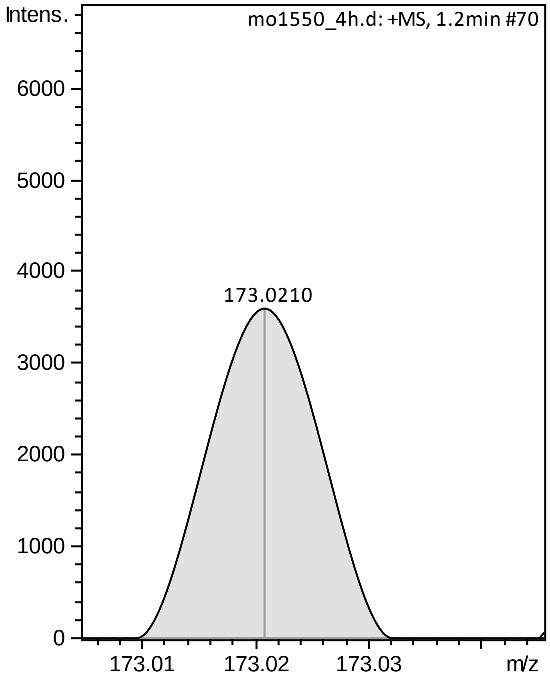

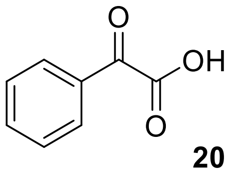

20

Exact Mass Calculated for $[M+Na]^+$ ($C_8H_6NaO_3^+$)	Exact Mass Found for $[M+Na]^+$ ($C_8H_6NaO_3^+$)
173.0209	173.0210

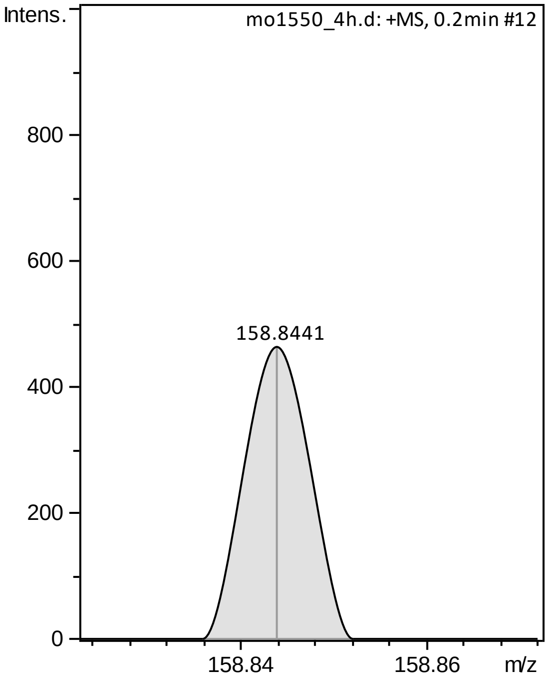

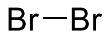

23

Exact Mass Calculated for $[M+H]^+$ (Br_2H^+)	Exact Mass Found for $[M+H]^+$ (Br_2H^+)
158.8440	158.8441

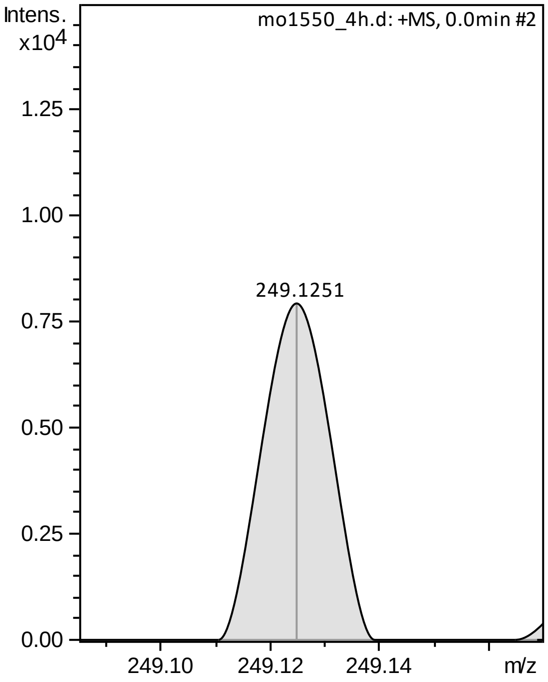

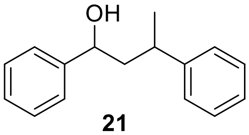

or

21

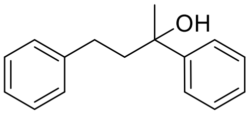

22

Exact Mass Calculated for $[M+Na]^+$ ($C_{16}H_{18}NaO^+$)	Exact Mass Found for $[M+Na]^+$ ($C_{16}H_{18}NaO^+$)
249.1250	249.1251

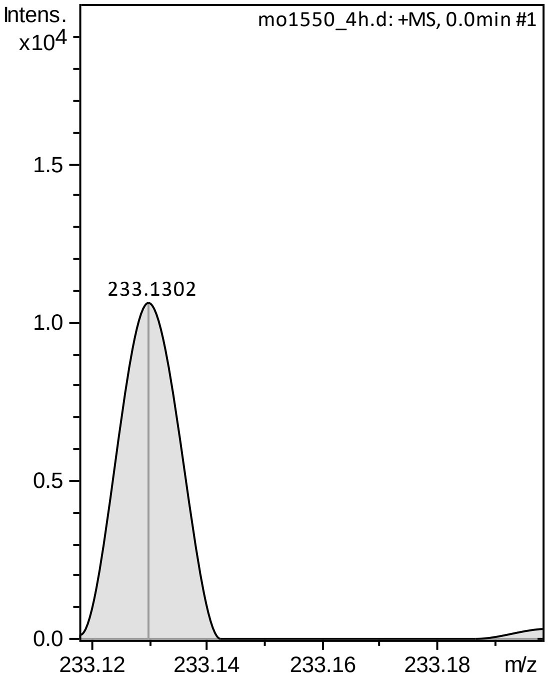

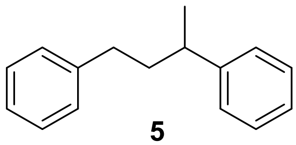

5

Exact Mass Calculated for $[M+Na]^+$ ($C_{16}H_{18}Na^+$)	Exact Mass Found for $[M+Na]^+$ ($C_{16}H_{18}Na^+$)
233.1301	233.1302

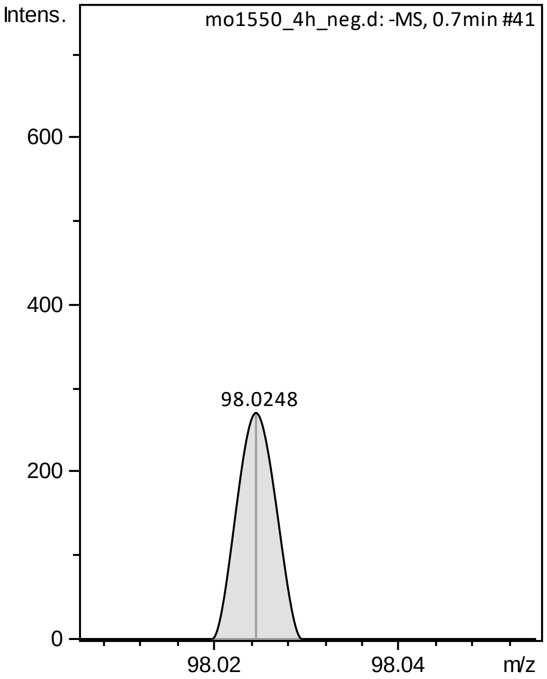

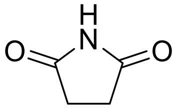

24

Exact Mass Calculated for $[M-H]^-$ ($C_4H_4NO_2^-$)	Exact Mass Found for $[M-H]^-$ ($C_4H_4NO_2^-$)
98.0248	98.0248

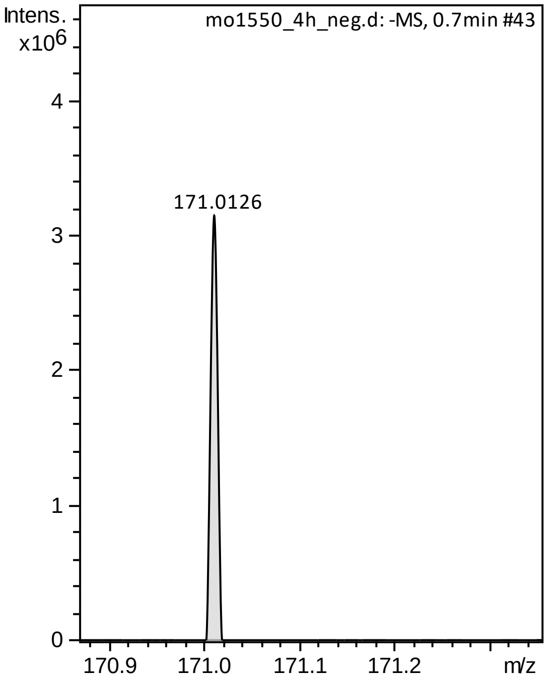

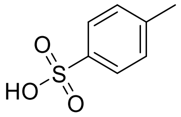

25

Exact Mass Calculated for [M-H]$^-$ ($C_7H_7O_3S^-$)	Exact Mass Found for [M-H]$^-$ ($C_7H_7O_3S^-$)
171.0121	171.0126

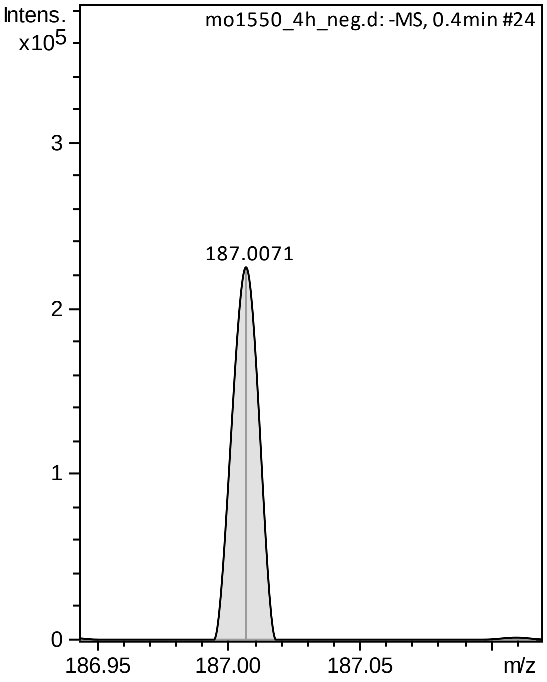

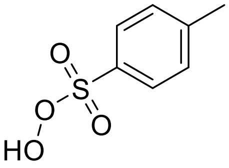

26

Exact Mass Calculated for [M-H]$^-$ ($C_7H_7O_4S^-$)	Exact Mass Found for [M-H]$^-$ ($C_7H_7O_4S^-$)
187.0071	187.0071

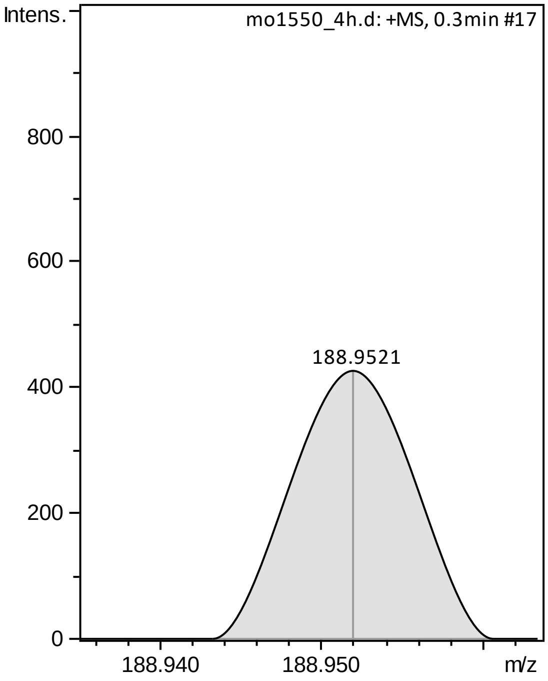

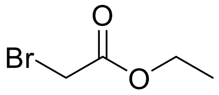

27

Exact Mass Calculated for $[M+Na]^+$ ($C_4H_7BrNaO_2^+$)	Exact Mass Found for $[M+Na]^+$ ($C_4H_7BrNaO_2^+$)
188.9522	188.9521

Intermediates observed after 18 h irradiation

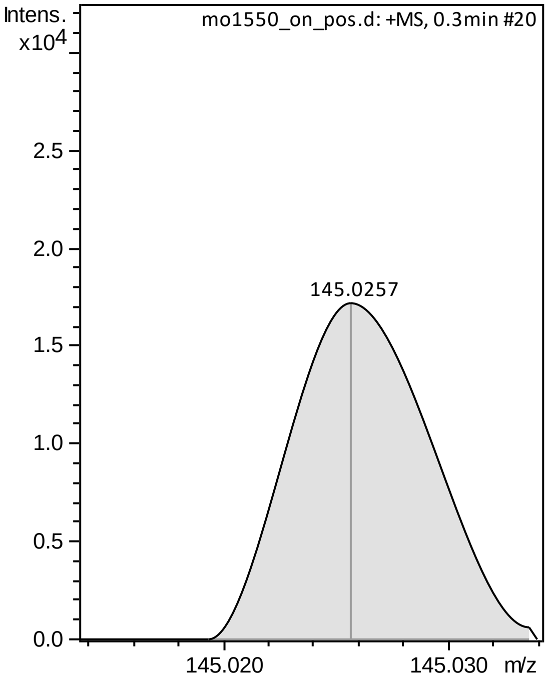

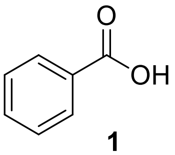

1

Exact Mass Calculated for $[M+Na]^+$ ($C_7H_6NaO_2^+$)	Exact Mass Found for $[M+Na]^+$ ($C_7H_6NaO_2^+$)
145.0260	145.0257

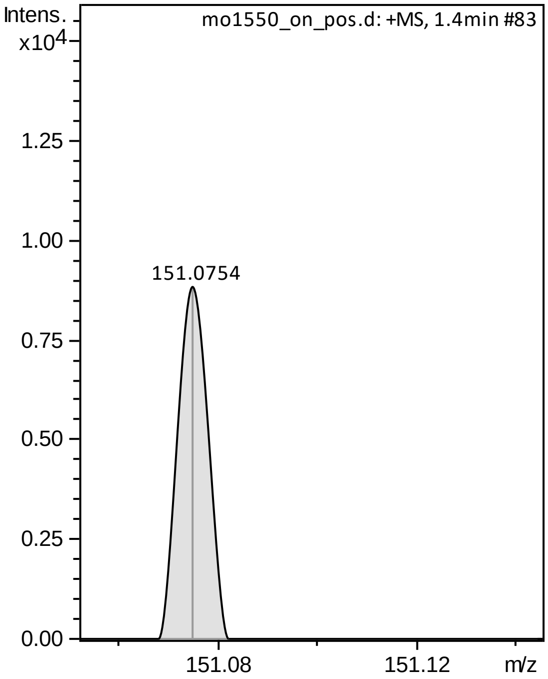

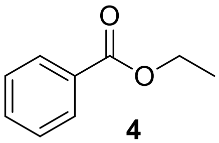

4

Exact Mass Calculated for [M+H]$^+$ ($C_9H_{11}O_2^+$)	Exact Mass Found for [M+H]$^+$ ($C_9H_{11}O_2^+$)
151.0754	151.0754

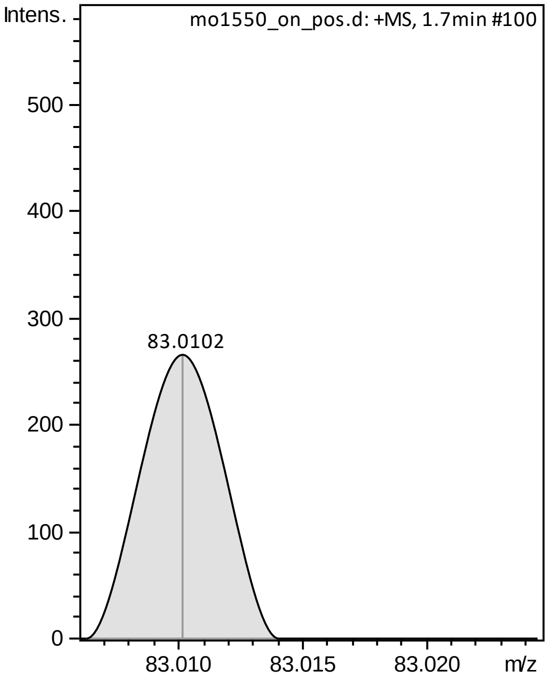

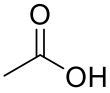

3

Exact Mass Calculated for [M+Na]$^+$ ($C_2H_4NaO_2^+$)	Exact Mass Found for [M+Na]$^+$ ($C_2H_4NaO_2^+$)
83.0104	83.0102

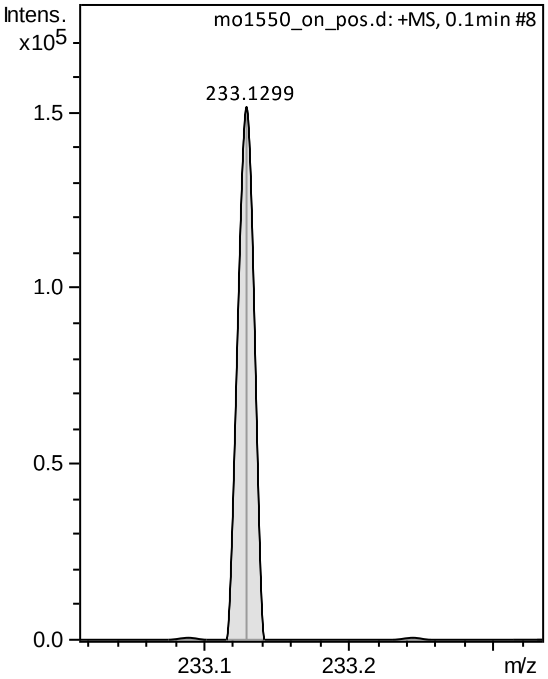

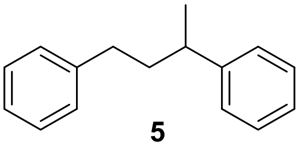

5

Exact Mass Calculated for $[M+Na]^+$ ($C_{16}H_{18}Na^+$)	Exact Mass Found for $[M+Na]^+$ ($C_{16}H_{18}Na^+$)
233.1301	233.1299

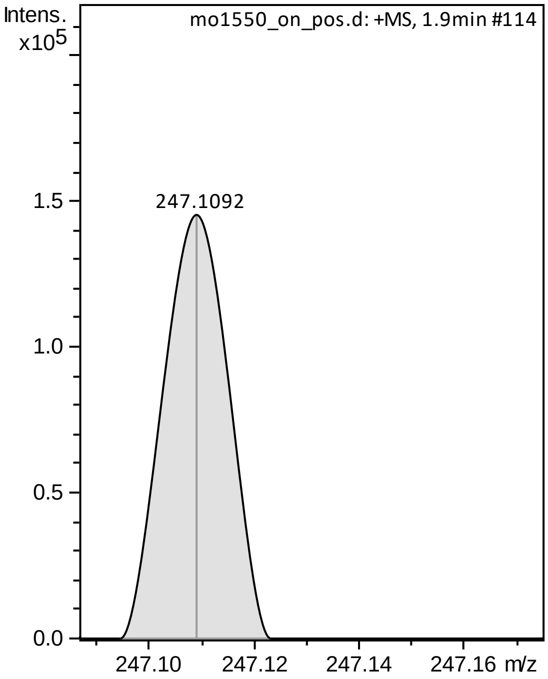

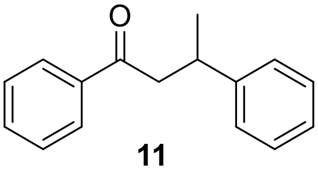

11

Exact Mass Calculated for $[M+Na]^+$ ($C_{16}H_{16}NaO^+$)	Exact Mass Found for $[M+Na]^+$ ($C_{16}H_{16}NaO^+$)
247.1093	247.1092

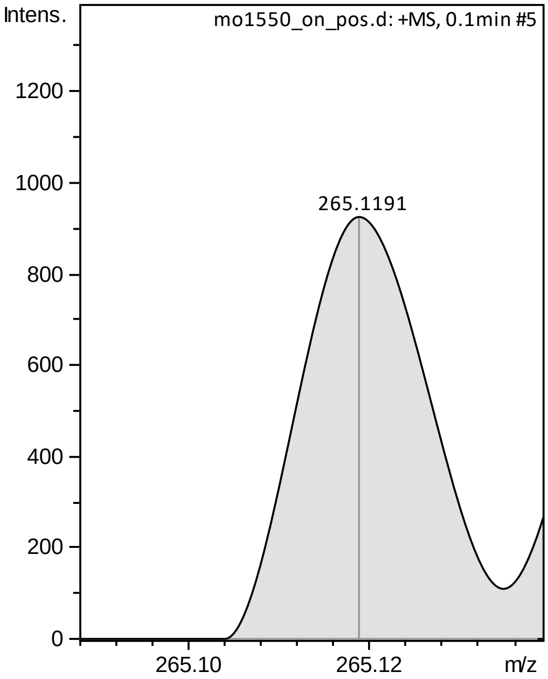

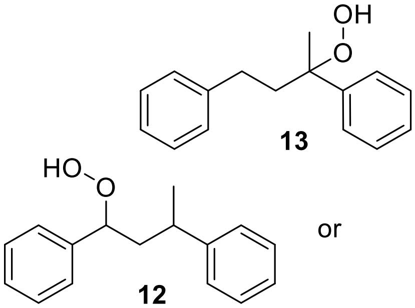

13

or

12

Exact Mass Calculated for $[M+Na]^+$ ($C_{16}H_{18}NaO_2^+$)	Exact Mass Found for $[M+Na]^+$ ($C_{16}H_{18}NaO_2^+$)
265.1199	265.1191

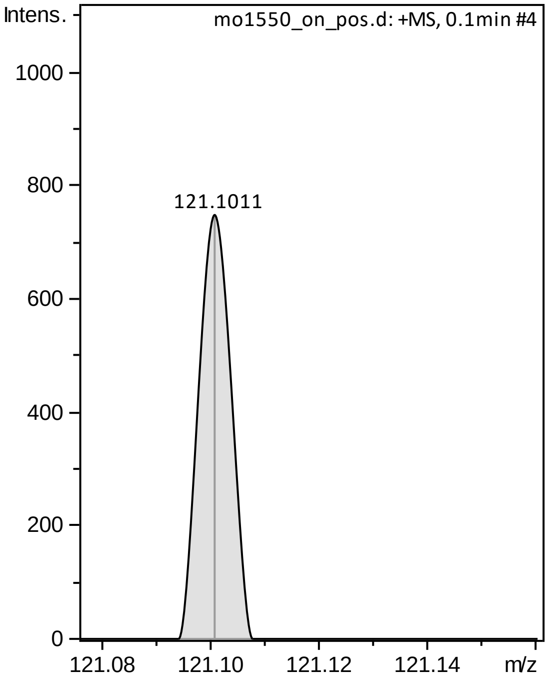

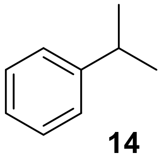

14

Exact Mass Calculated for $[M+H]^+$ ($C_9H_{13}^+$)	Exact Mass Found for $[M+H]^+$ ($C_9H_{13}^+$)
121.1012	121.1011

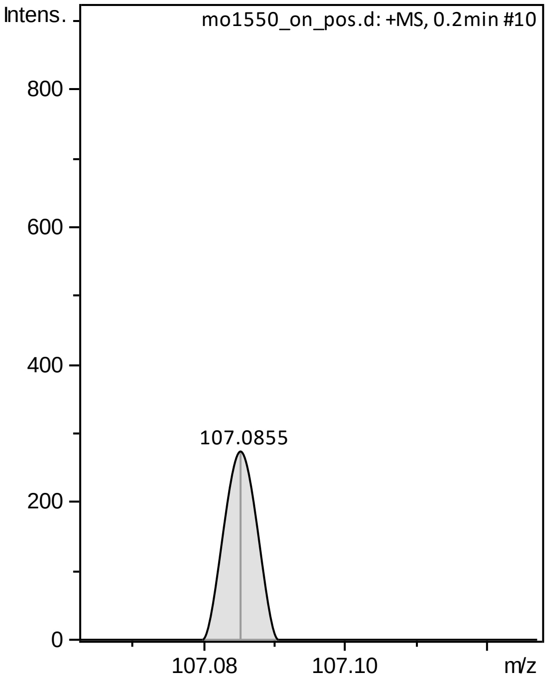

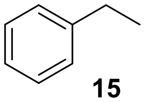

15

Exact Mass Calculated for $[M+H]^+$ ($C_8H_{11}^+$)	Exact Mass Found for $[M+H]^+$ ($C_8H_{11}^+$)
107.0855	107.0855

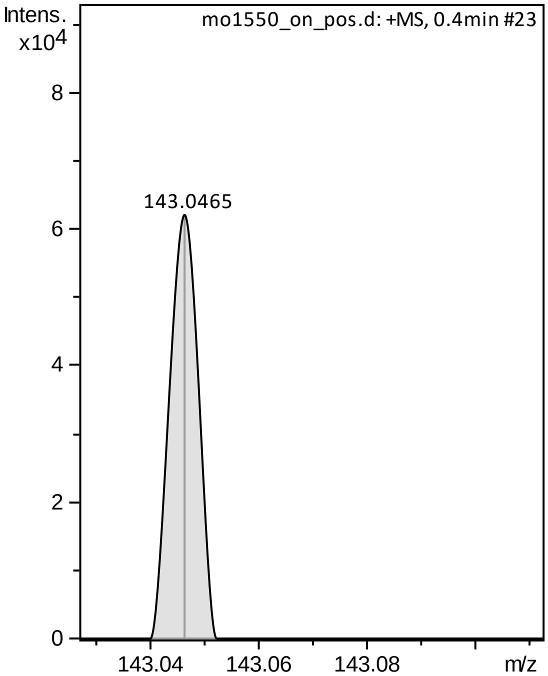

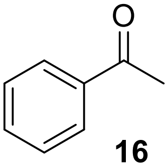

16

Exact Mass Calculated for $[M+Na]^+$ ($C_8H_8NaO^+$)	Exact Mass Found for $[M+Na]^+$ ($C_8H_8NaO^+$)
143.0467	143.0465

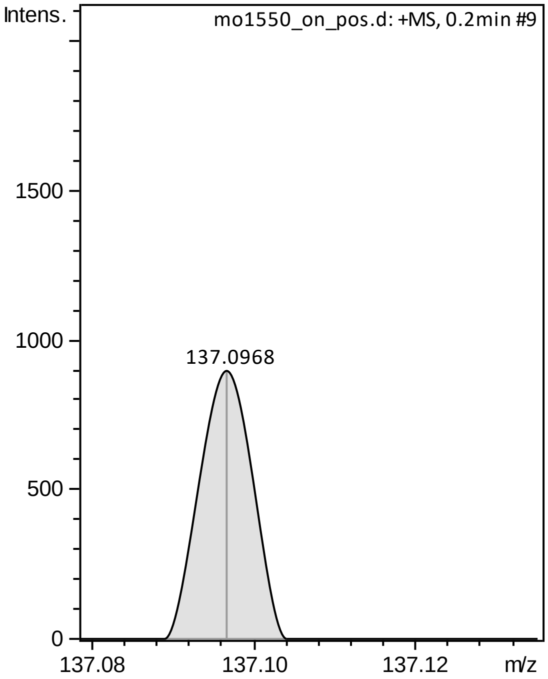

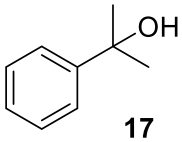

17

Exact Mass Calculated for $[M+H]^+$ ($C_9H_{13}O^+$)	Exact Mass Found for $[M+H]^+$ ($C_9H_{13}O^+$)
137.0961	137.0968

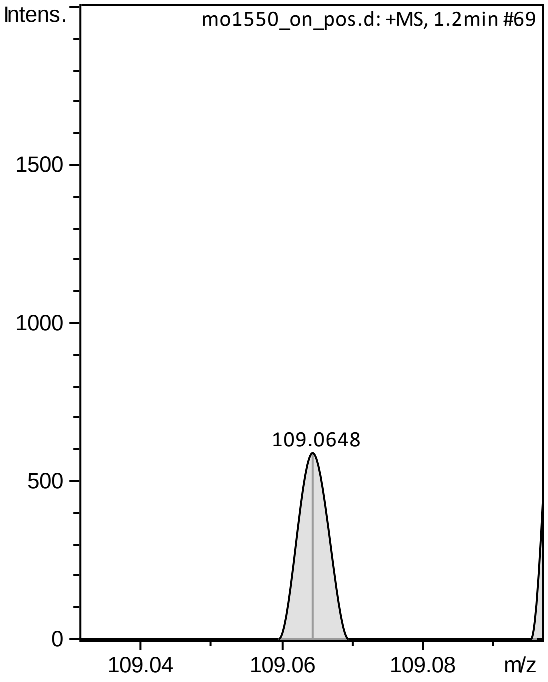

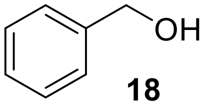

18

Exact Mass Calculated for $[M+H]^+$ ($C_7H_9O^+$)	Exact Mass Found for $[M+H]^+$ ($C_7H_9O^+$)
109.0648	109.0648

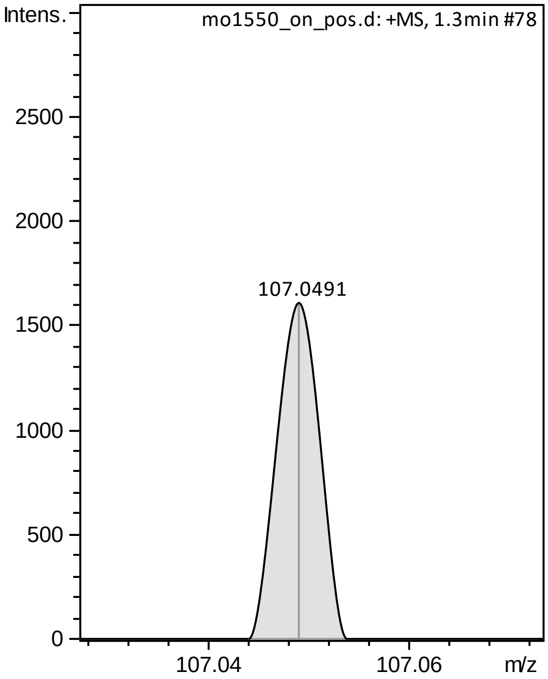

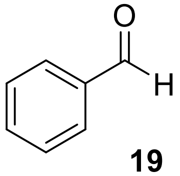

19

Exact Mass Calculated for $[M+H]^+$ ($C_7H_7O^+$)	Exact Mass Found for $[M+H]^+$ ($C_7H_7O^+$)
107.0491	107.0491

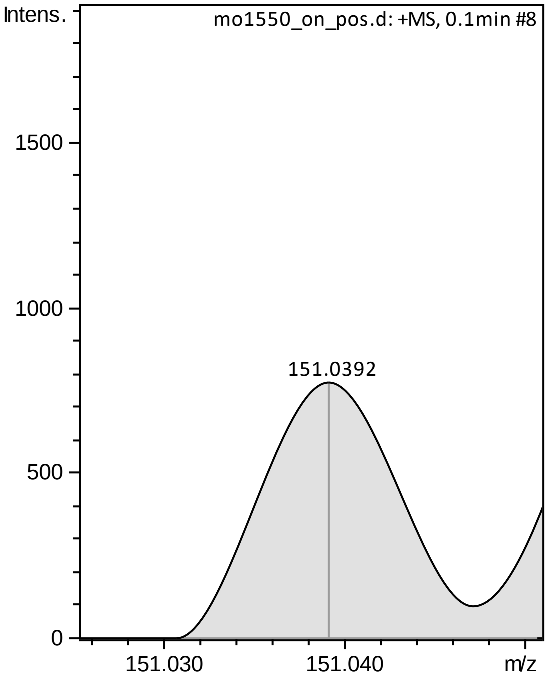

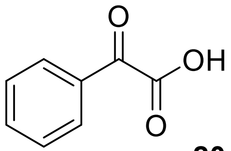

20

Exact Mass Calculated for $[M+H]^+$ ($C_8H_7O_3^+$)	Exact Mass Found for $[M+H]^+$ ($C_8H_7O_3^+$)
151.0390	151.0392

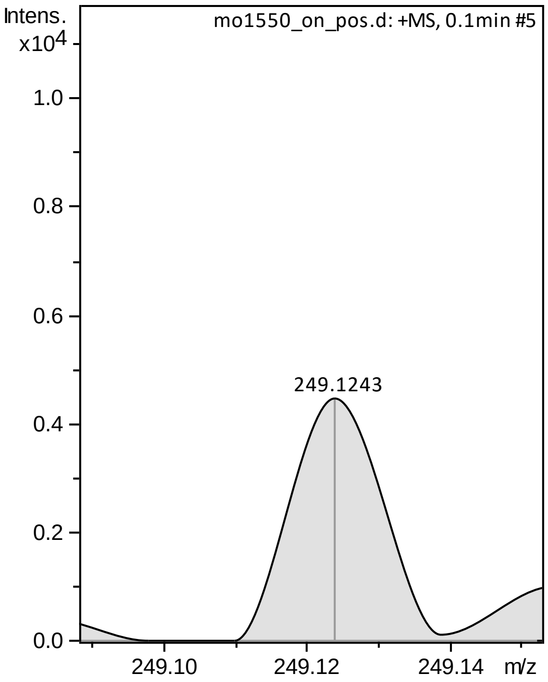

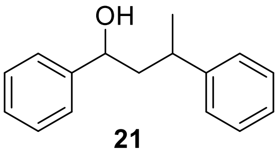

or

21

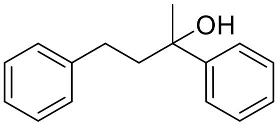

22

Exact Mass Calculated for [M+Na]$^+$ ($C_{16}H_{18}NaO^+$)	Exact Mass Found for [M+Na]$^+$ ($C_{16}H_{18}NaO^+$)
249.1250	249.1243

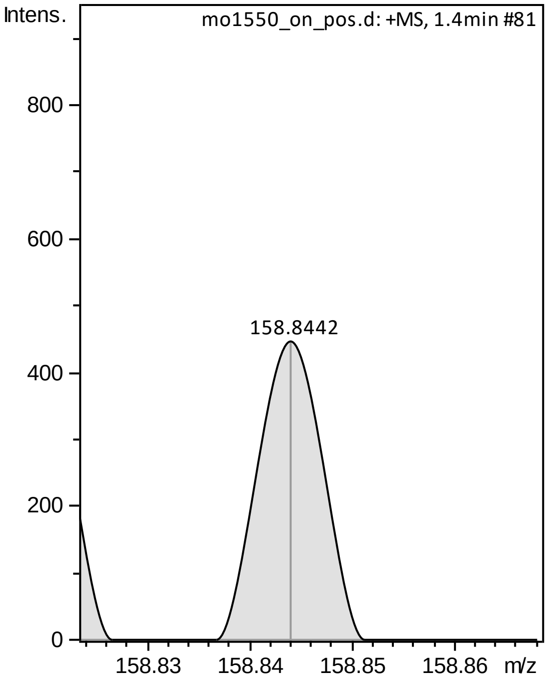

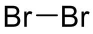

23

Exact Mass Calculated for [M+H]$^+$ (Br_2H^+)	Exact Mass Found for [M+H]$^+$ (Br_2H^+)
158.8440	158.8442

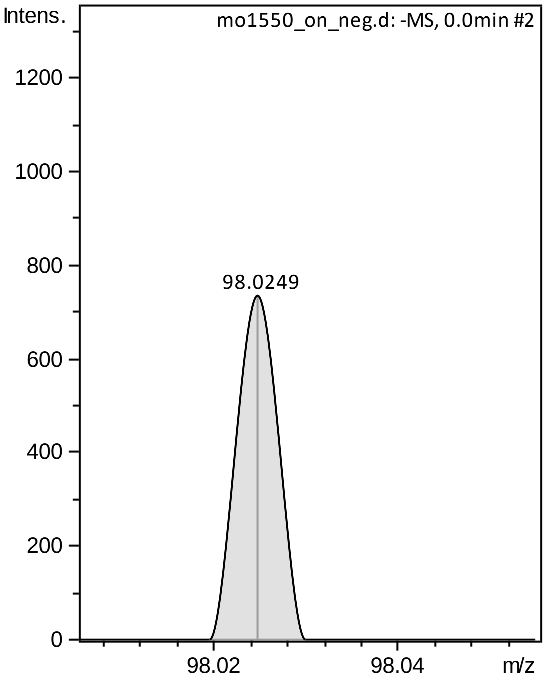

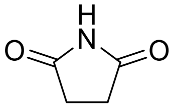

24

Exact Mass Calculated for [M-H]$^-$ ($C_4H_4NO_2^-$)	Exact Mass Found for [M-H]$^-$ ($C_4H_4NO_2^-$)
98.0248	98.0249

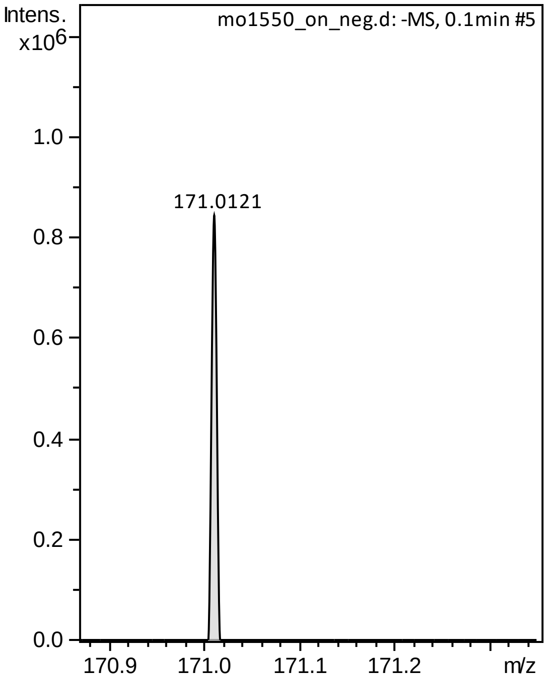

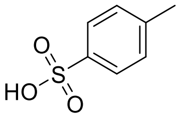

25

Exact Mass Calculated for [M-H]$^-$ ($C_7H_7O_3S^-$)	Exact Mass Found for [M-H]$^-$ ($C_7H_7O_3S^-$)
171.0121	171.0121

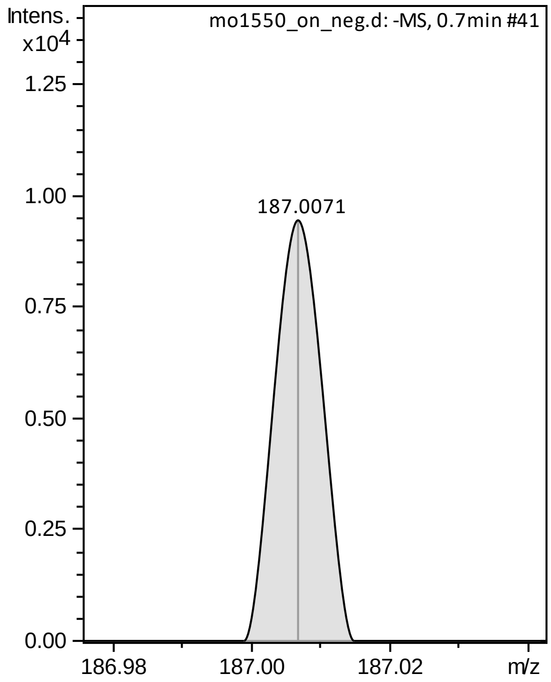

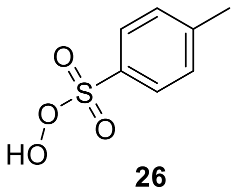

26

Exact Mass Calculated for [M-H]$^-$ ($C_7H_7O_4S^-$)	Exact Mass Found for [M-H]$^-$ ($C_7H_7O_4S^-$)
187.0071	187.0071

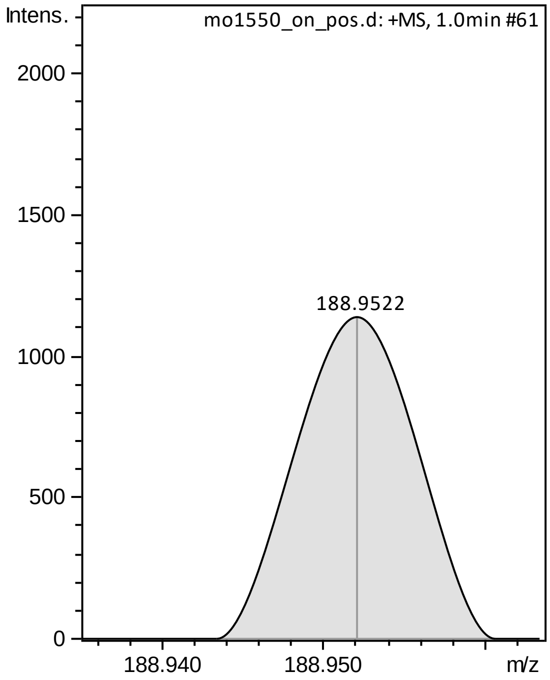

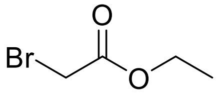

27

Exact Mass Calculated for [M+Na]$^+$ ($C_4H_7BrNaO_2^+$)	Exact Mass Found for [M+Na]$^+$ ($C_4H_7BrNaO_2^+$)
188.9522	188.9522

Products of the photochemical oxidation of butane-1,3-diyldibenzene (5) under 370 nm 2nd generation Kessil lamp irradiation in the presence of TEMPO as the radical scavenger

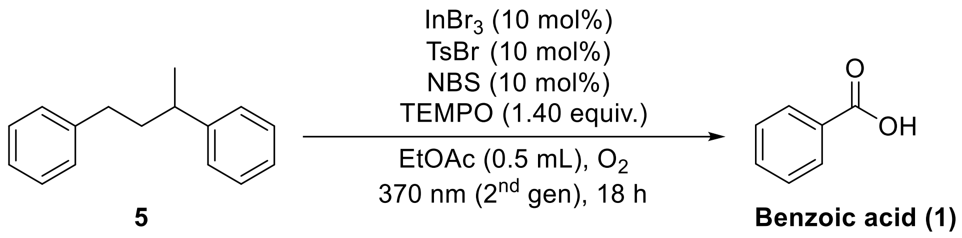

The products of the oxidation of the photochemical cleavage of butane-1,3-diyldibenzene (**5**), upon irradiation with 370 nm 2nd generation Kessil lamp, were monitored for 18 hours by DI-HRMS. Butane-1,3-diyldibenzene (**5**) (21 mg, 0.10 mmol), indium tribromide ($InBr_3$) (3.6 mg, 0.01 mmol, 0.10 equiv.), tosyl bromide (TsBr) (2.4 mg, 0.01 mmol, 0.10 equiv.), *N*-bromosuccinimide (NBS) (1.8 mg, 0.01 mmol, 0.10 equiv.) and TEMPO (21.9 mg, 0.14 mmol, 1.40 equiv.) were dissolved in EtOAc (0.5 mL). The reaction mixture was sealed with a septum and parafilm. Then, a first degassing was performed using vacuum and argon flow, followed by a second degassing using oxygen flow. The atmosphere in the test tube was maintained by attaching two balloons filled with oxygen. The reaction mixture was stirred and irradiated with a 2nd generation Kessil lamp at 370 nm for 18 hours. After 18 hours, a sample of the reaction mixture (10 μL) was first diluted with 990 μL methanol and 100 μL of that sample were further diluted with 900 μL of methanol. Finally, 100 μL were injected for DI-HRMS analysis.

A suspect analysis approach revealed peaks corresponding to intermediates **28-39**.

Intermediates observed after 18 h irradiation

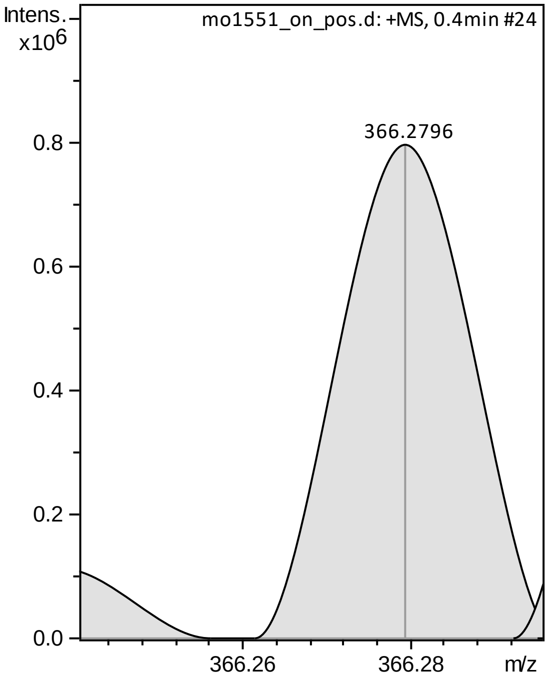

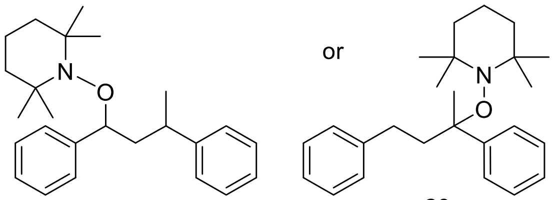

28 **29**

Exact Mass Calculated for $[M+H]^+$ ($C_{25}H_{36}NO^+$)	Exact Mass Found for $[M+H]^+$ ($C_{25}H_{36}NO^+$)
366.2791	366.2796

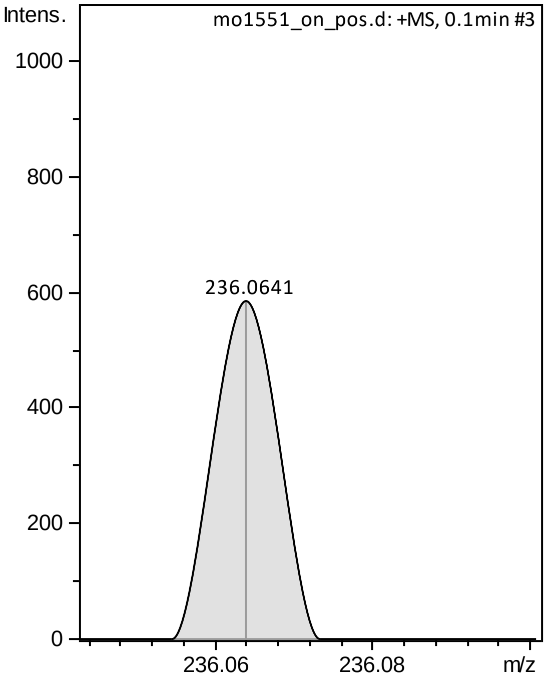

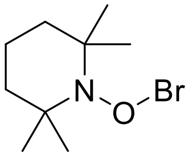

30

Exact Mass Calculated for $[M+H]^+$ ($C_9H_{19}BrNO^+$)	Exact Mass Found for $[M+H]^+$ ($C_9H_{19}BrNO^+$)
236.0645	236.0641

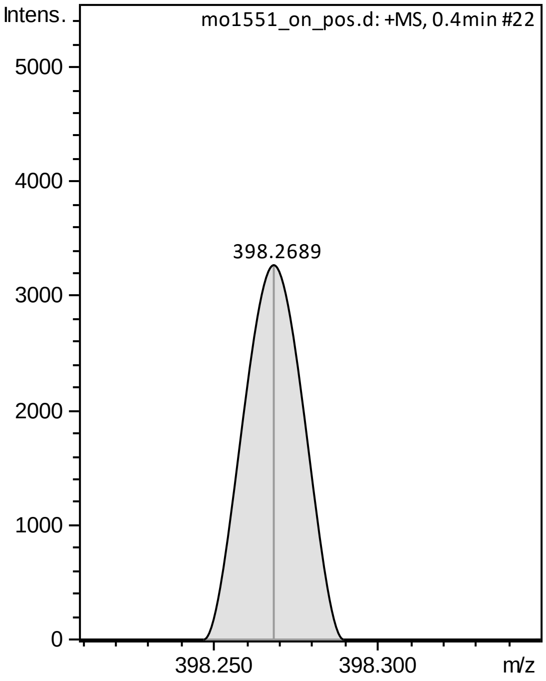

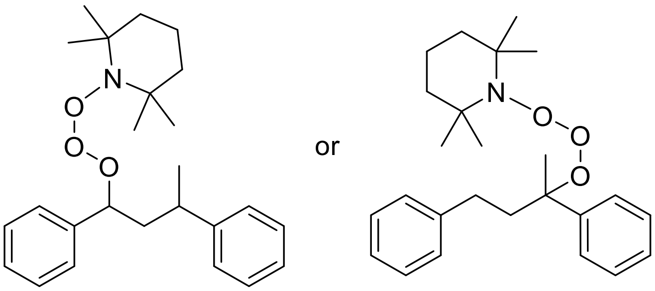

31 or 32

Exact Mass Calculated for $[M+H]^+$ ($C_{25}H_{36}NO_3^+$)	Exact Mass Found for $[M+H]^+$ ($C_{25}H_{36}NO_3^+$)
398.2690	398.2689

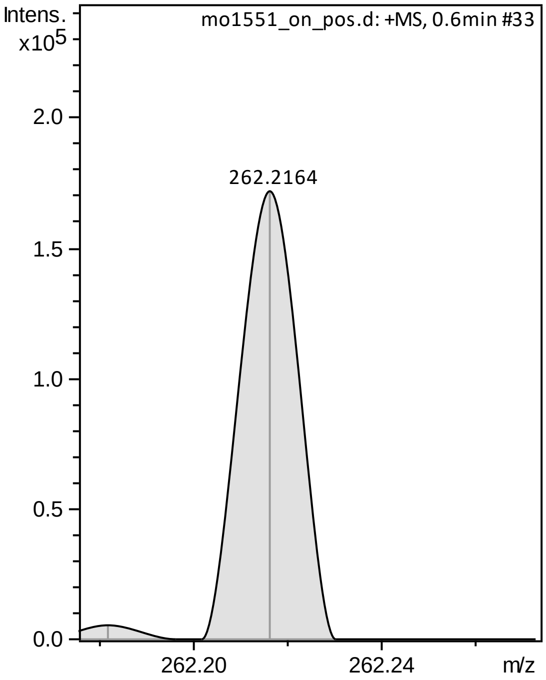

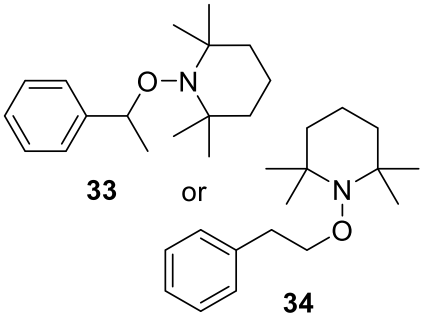

33 or 34

Exact Mass Calculated for $[M+H]^+$ ($C_{17}H_{28}NO^+$)	Exact Mass Found for $[M+H]^+$ ($C_{17}H_{28}NO^+$)
262.2165	262.2164

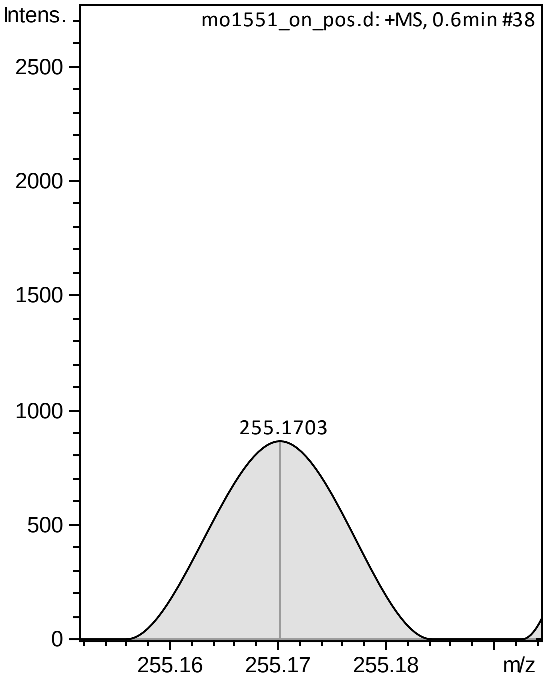

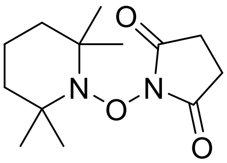

35

Exact Mass Calculated for [M+H]$^+$ ($C_{13}H_{23}N_2O_3^+$)	Exact Mass Found for [M+H]$^+$ ($C_{13}H_{23}N_2O_3^+$)
255.1703	255.1703

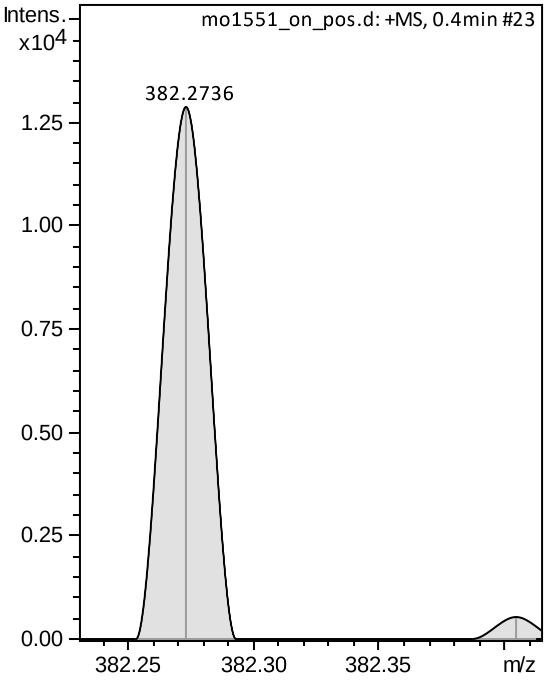

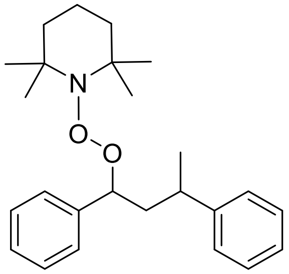

or

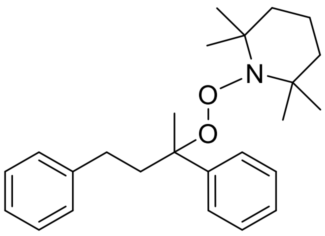

36 **37**

Exact Mass Calculated for [M+H]$^+$ ($C_{25}H_{36}NO_2^+$)	Exact Mass Found for [M+H]$^+$ ($C_{25}H_{36}NO_2^+$)
382.2741	382.2736

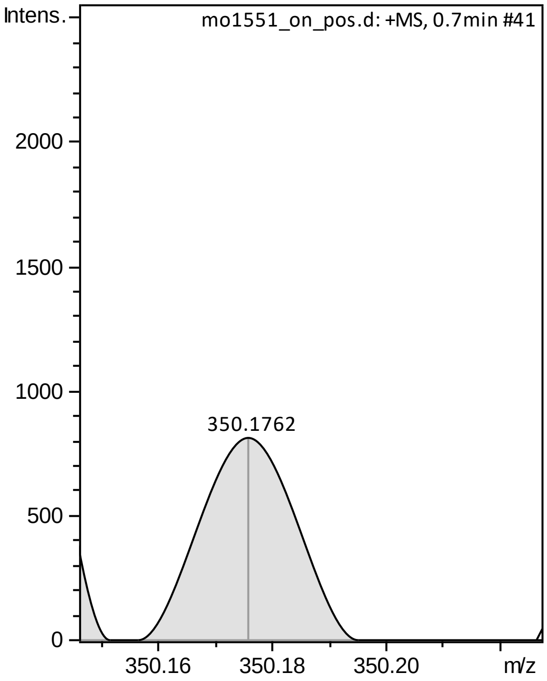

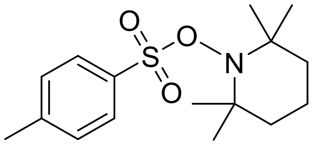

38

Exact Mass Calculated for [M+Na]$^+$ ($C_{17}H_{29}NNaO_3S^+$)	Exact Mass Found for [M+Na]$^+$ ($C_{17}H_{29}NNaO_3S^+$)
350.1760	350.1762

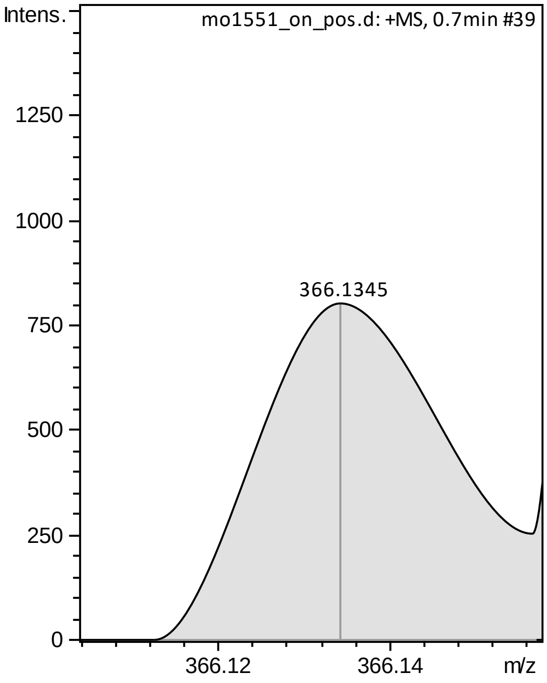

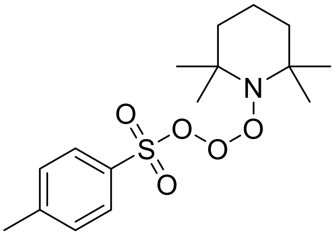

39

Exact Mass Calculated for [M+Na]$^+$ ($C_{16}H_{25}NNaO_5S^+$)	Exact Mass Found for [M+Na]$^+$ ($C_{16}H_{25}NNaO_5S^+$)
366.1346	366.1345

Further Mechanistic Studies

As the proposed mechanism involves the formation of a bromide-rich indium species, the $[InBr_4]^-$ complex was independently synthesized to confirm its accessibility under the reaction conditions. Since tosyl bromide and NBS are unable to furnish bromide anions directly without photochemical activation and radical pathways, tetrabutylammonium bromide was employed as the external bromide source, providing both the necessary halide and a stabilizing counterion. This preparation enabled observation of the complex by HRMS and assessment of its stability, thereby supporting its relevance to the mechanistic proposal.

Synthesis of $[NBu_4]^+[InBr_4]^-$ complex

A solution of $InBr_3$ (89 mg, 0.25 mmol, 1.00 equiv.) in ethanol (0.25 mL) was combined with a solution of TBABr (85 mg, 0.26 mmol, 1.05 equiv.) in ethanol (0.25 mL). Upon mixing, precipitation of a white solid was observed. The suspension was stirred at room temperature for 2 h, after which the solid was collected by filtration, washed thoroughly with cold ethanol (3 x 0.5 mL) to remove excess tetrabutylammonium salts and residual $InBr_3$, and dried under vacuum to afford 139 mg of $[NBu_4]^+[InBr_4]^-$ as a white crystalline solid. **HRMS** exact mass calculated for $[M]^-$ ($InBr_4^-$) requires *m/z* 434.5737, found *m/z* 434.5735.

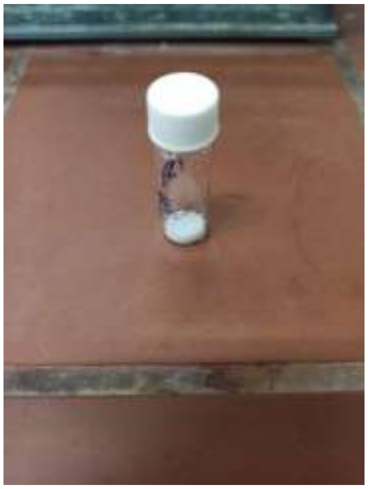

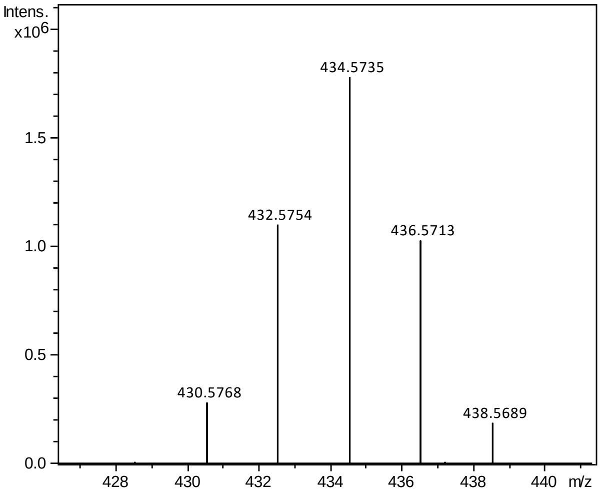

The independently synthesized $[NBu_4]^+[InBr_4]^-$ complex was subsequently employed in the photochemical reaction of PS, both on its own and in combination with TsBr or NBS individually.

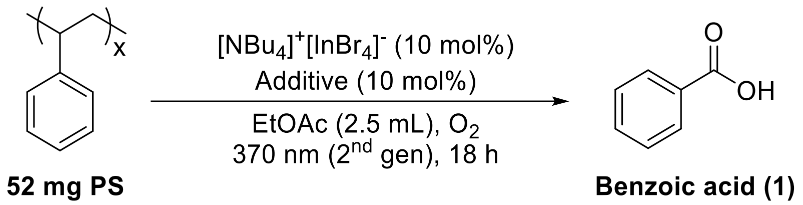

Entry	Complex	Additive	Yield (%)[a]
1	$[NBu_4]^+[InBr_4]^-$	-	22
2	$[NBu_4]^+[InBr_4]^-$	TsBr	34
3	$[NBu_4]^+[InBr_4]^-$	NBS	34

[a] Yield of isolated product, after base-acid wash and extractions.

We next analyzed the reaction mixture of the photochemical reaction of butane-1,3-diyldibenzene (**5**) under the optimum conditions and we were able to detect the $[InBr_4]^-$ species' peak at high intensity, consistently observed throughout the entire course of the study (at 1, 2, 3, 4, and 18 h).

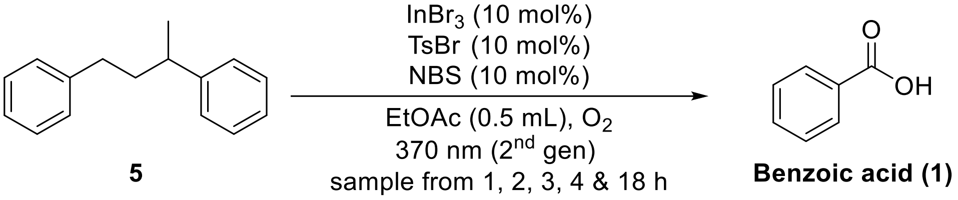

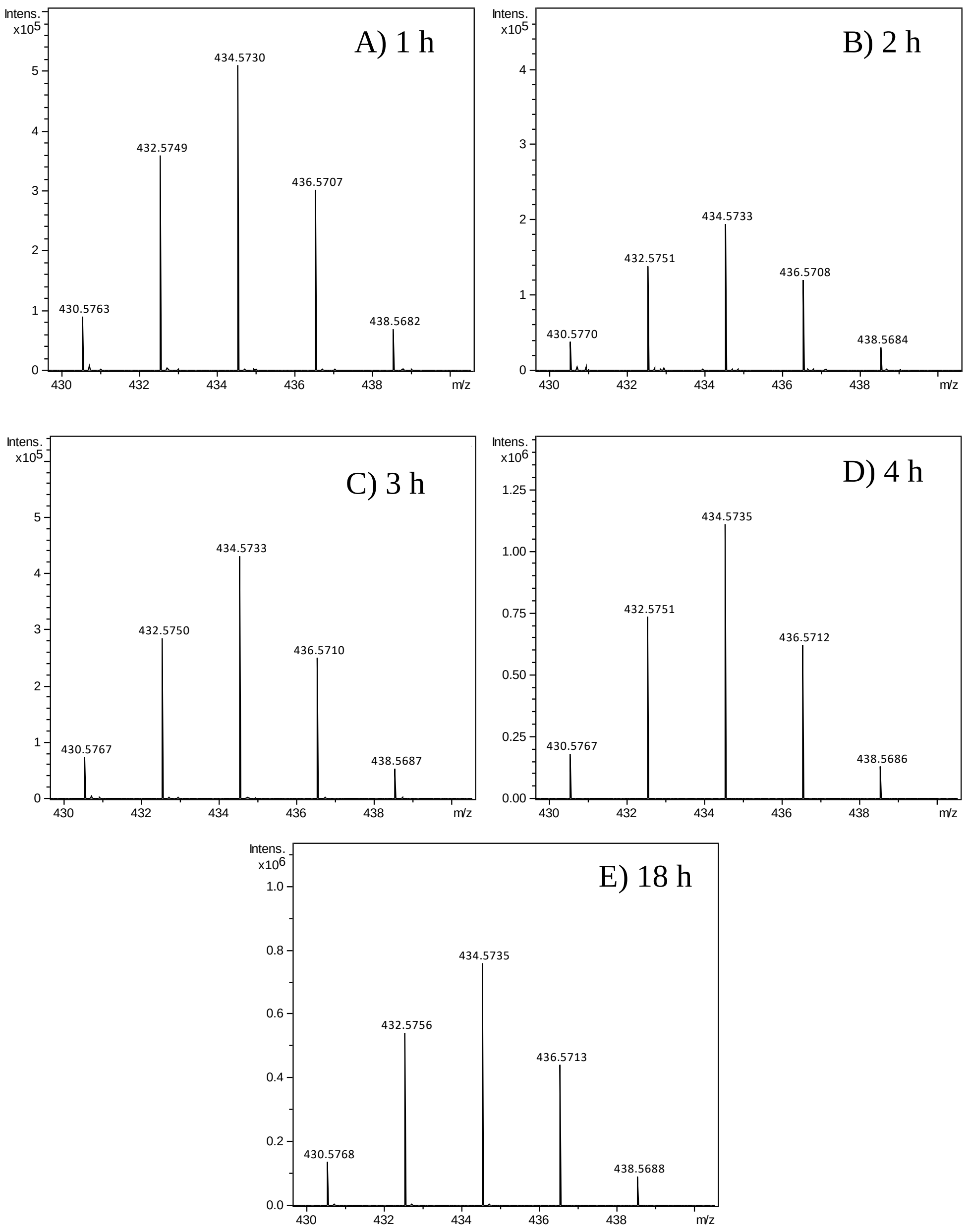

To determine whether tosyl bromide and *N*-bromosuccinimide (NBS) act through the same pathway to produce the $[InBr_4]^-$ species photochemically, each reagent was tested individually under identical conditions. In both cases, formation of the $[InBr_4]^-$ complex was confirmed.

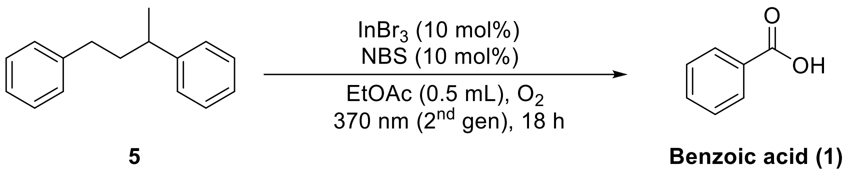

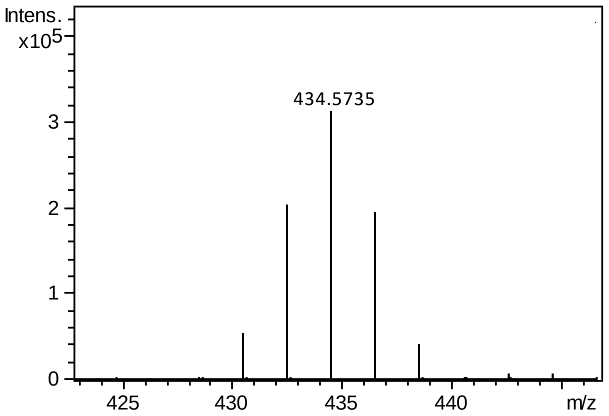

Peaks corresponding to $[InBr_4]^-$ detected in the photochemical upcycling reaction of PS to benzoic acid using $InBr_3$ and *N*-bromosuccinimide

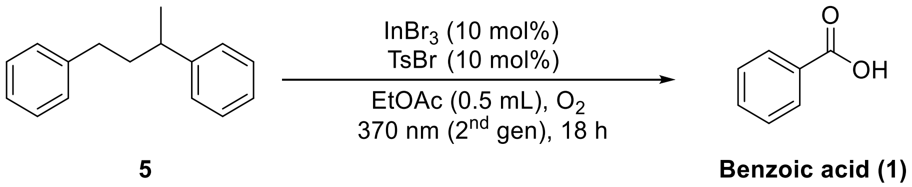

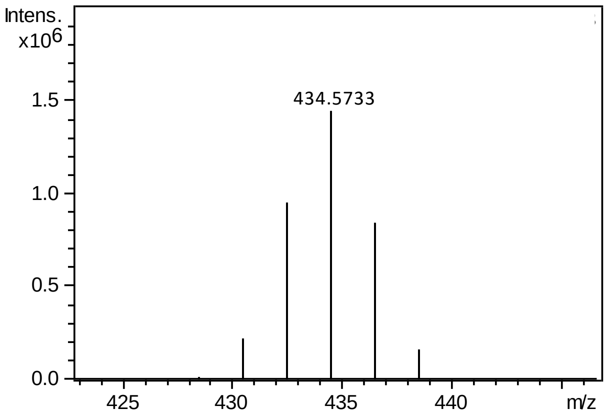

Peaks corresponding to $[InBr_4]^-$ detected in the photochemical upcycling reaction of PS to benzoic acid using $InBr_3$ and tosyl bromide

Mechanistic Studies

UV-Vis Spectra

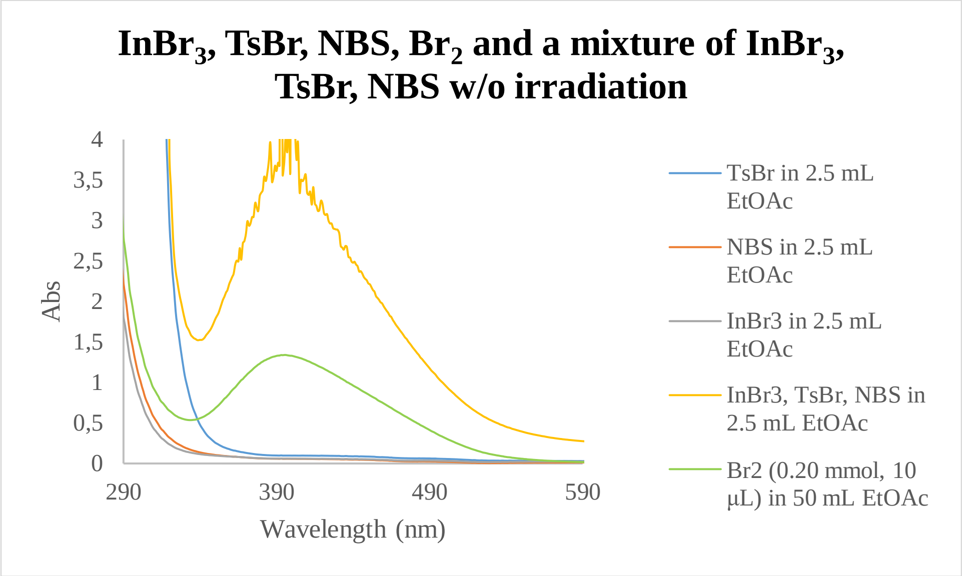

Figure S5. UV-Vis spectra of $InBr_3$ (0.05 mmol), TsBr (0.05 mmol), NBS (0.05 mmol), a mixture of $InBr_3$ (0.05 mmol), TsBr (0.05 mmol), NBS (0.05 mmol) in 2.5 mL EtOAc, and Br_2 (0.20 mmol, 10 µL) in 50 mL EtOAc without irradiation.

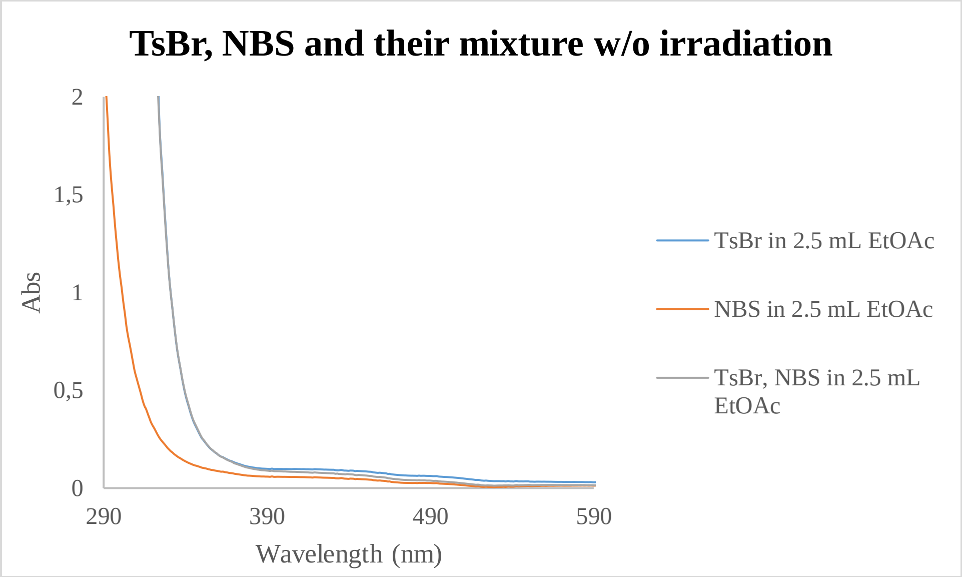

Figure S6. UV-Vis spectra of TsBr (0.05 mmol), NBS (0.05 mmol) and their mixture in 2.5 mL EtOAc without irradiation.

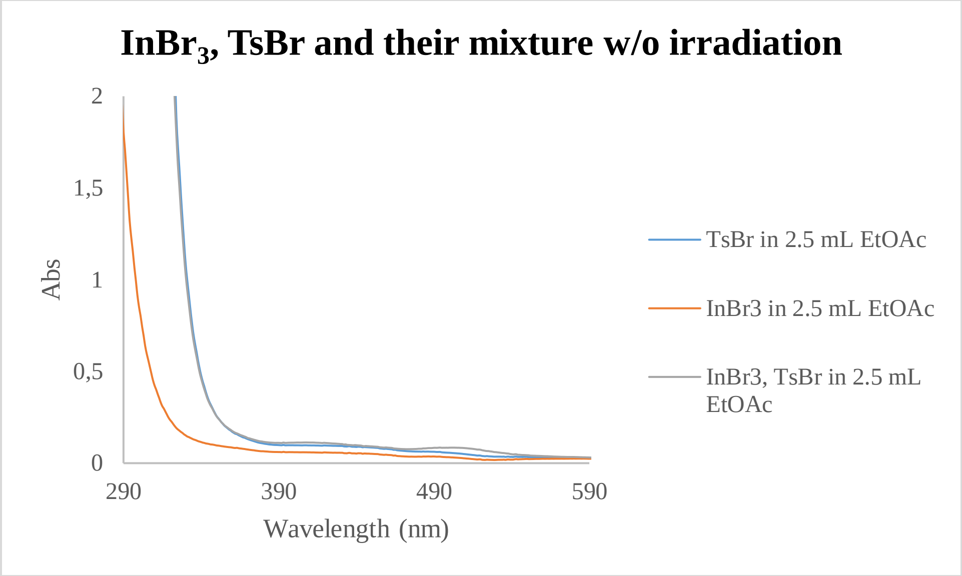

Figure S7. UV-Vis spectra of $InBr_3$ (0.05 mmol), TsBr (0.05 mmol) and their mixture in 2.5 mL EtOAc without irradiation.

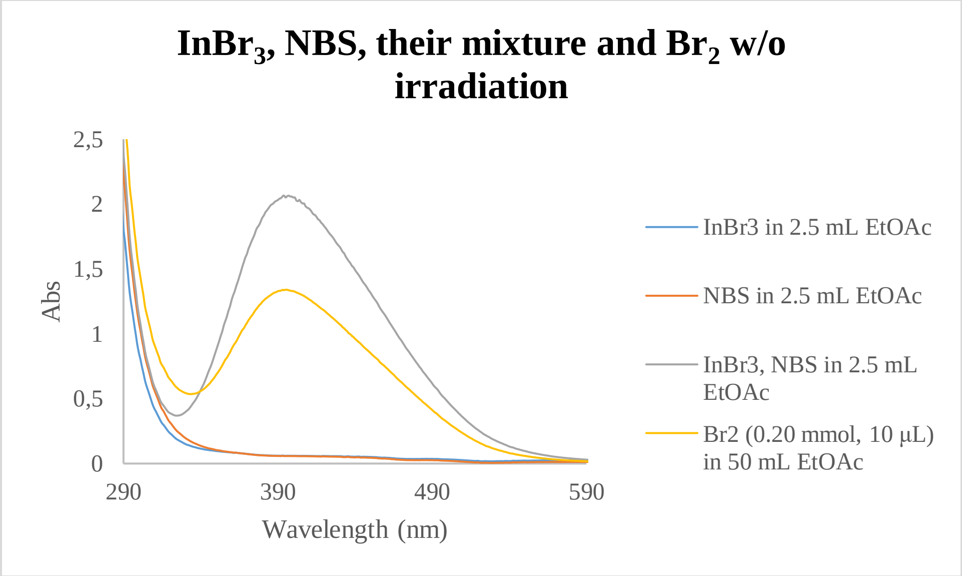

Figure S8. UV-Vis spectra of $InBr_3$ (0.05 mmol), NBS (0.05 mmol), their mixture in 2.5 mL EtOAc, and Br_2 (0.20 mmol, 10 µL) in 50 mL EtOAc without irradiation.

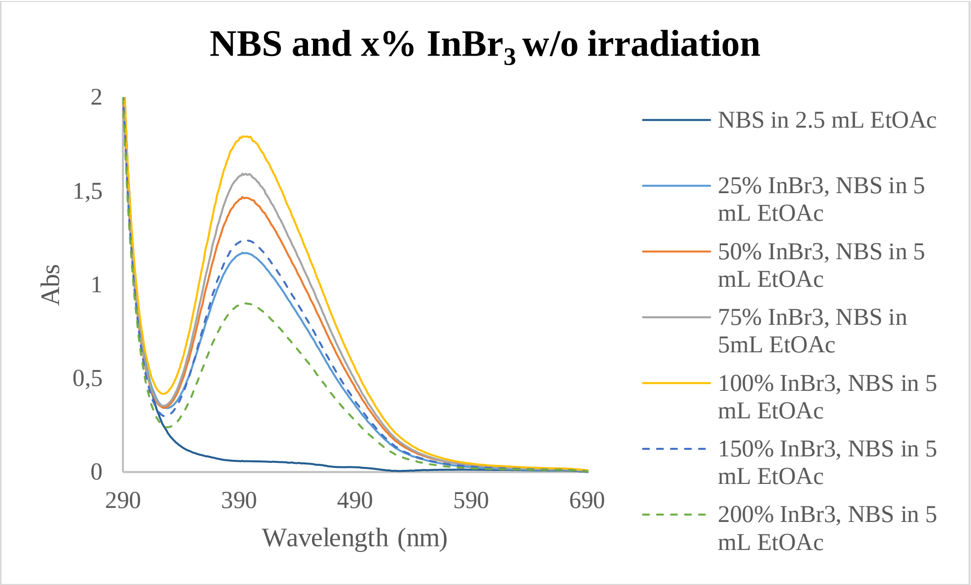

Figure S9. UV-Vis spectra of NBS (0.05 mmol) and $InBr_3$ (0.0125 mmol, 0.0250 mmol, 0.0375 mmol, 0.0500 mmol, 0.0750 mmol, 0.100 mmol) mixture in proportional ratios in 2.5 mL EtOAc without irradiation.

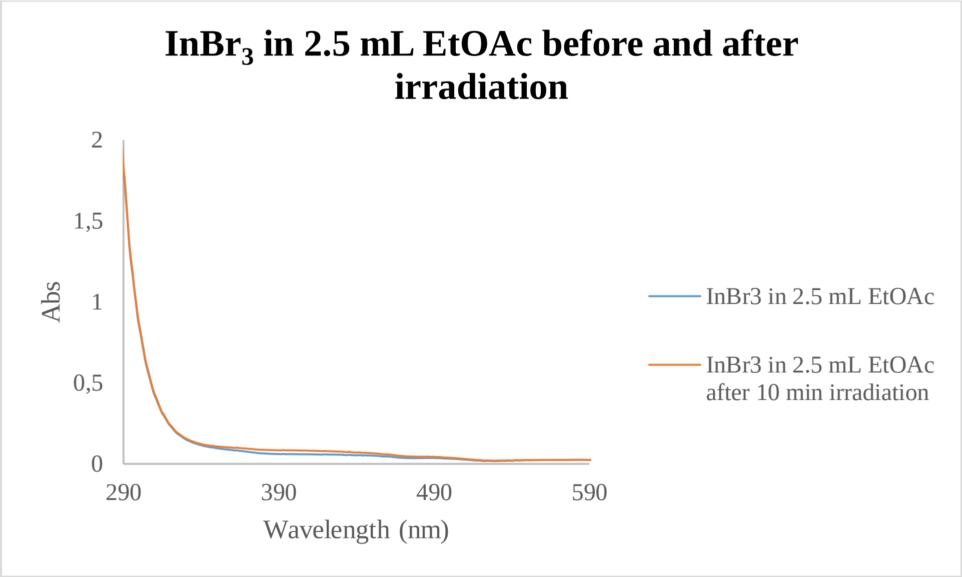

Figure S10. UV-Vis spectra of $InBr_3$ (0.05 mmol) in 2.5 mL EtOAc before and after 10 min of irradiation.

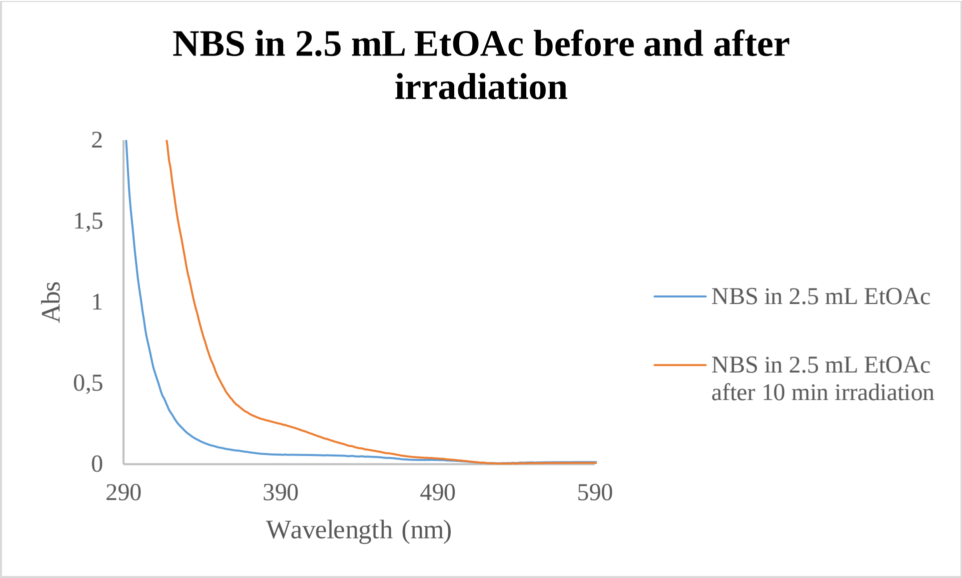

Figure S11. UV-Vis spectra of NBS (0.05 mmol) in 2.5 mL EtOAc before and after 10 min of irradiation.

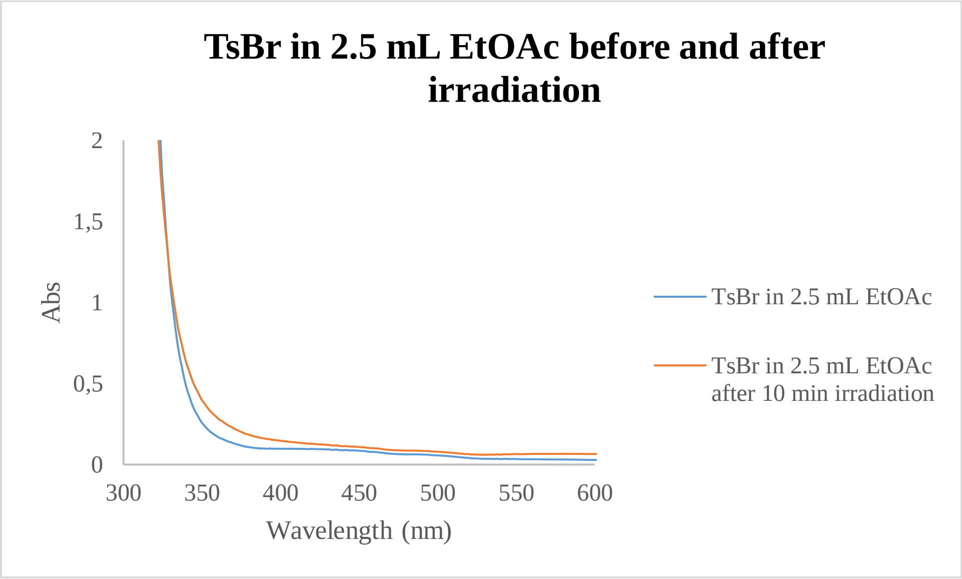

Figure S12. UV-Vis spectra of TsBr (0.05 mmol) in 2.5 mL EtOAc before and after 10 min of irradiation.

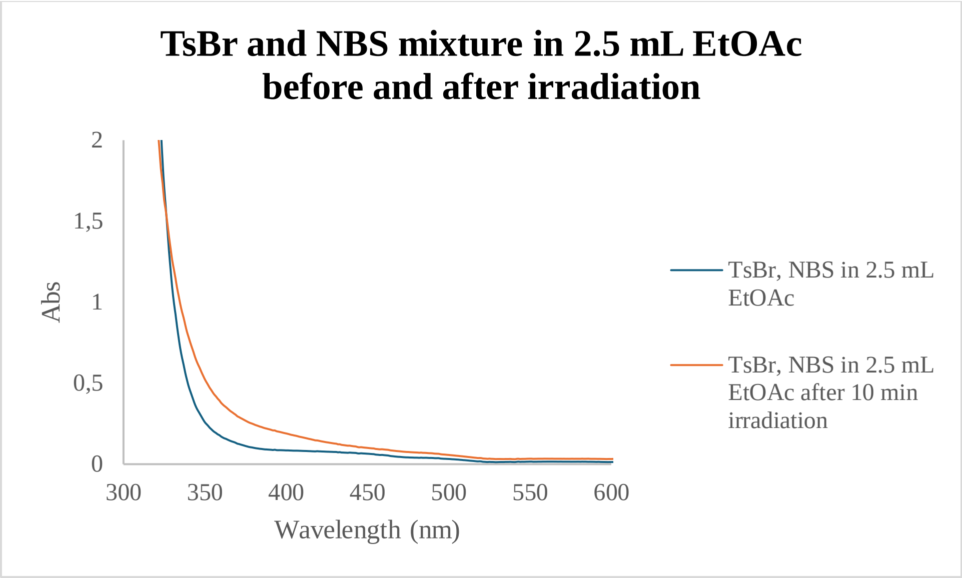

Figure S13. UV-Vis spectra of TsBr (0.05 mmol) and NBS (0.05 mmol) mixture in 2.5 mL EtOAc before and after 10 min of irradiation.

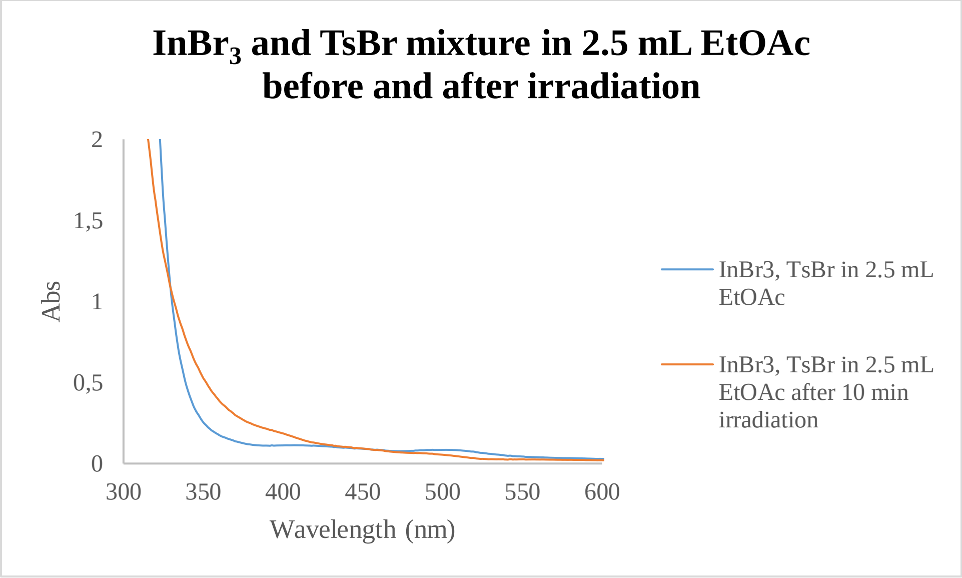

Figure S14. UV-Vis spectra of $InBr_3$ (0.05 mmol) and TsBr (0.05 mmol) mixture in 2.5 mL EtOAc before and after 10 min of irradiation.

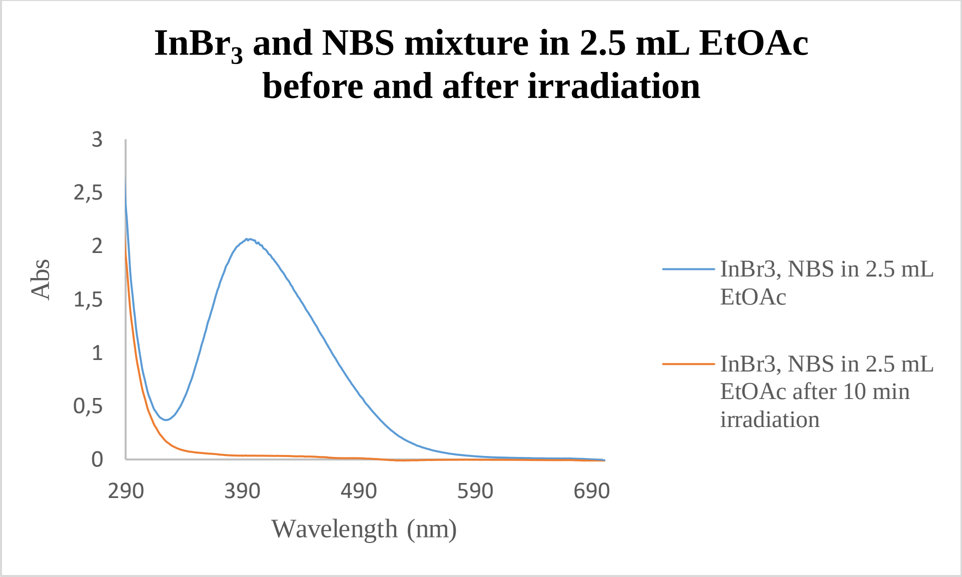

Figure S15. UV-Vis spectra of $InBr_3$ (0.05 mmol) and NBS (0.05 mmol) mixture in 2.5 mL EtOAc before and after 10 min of irradiation.

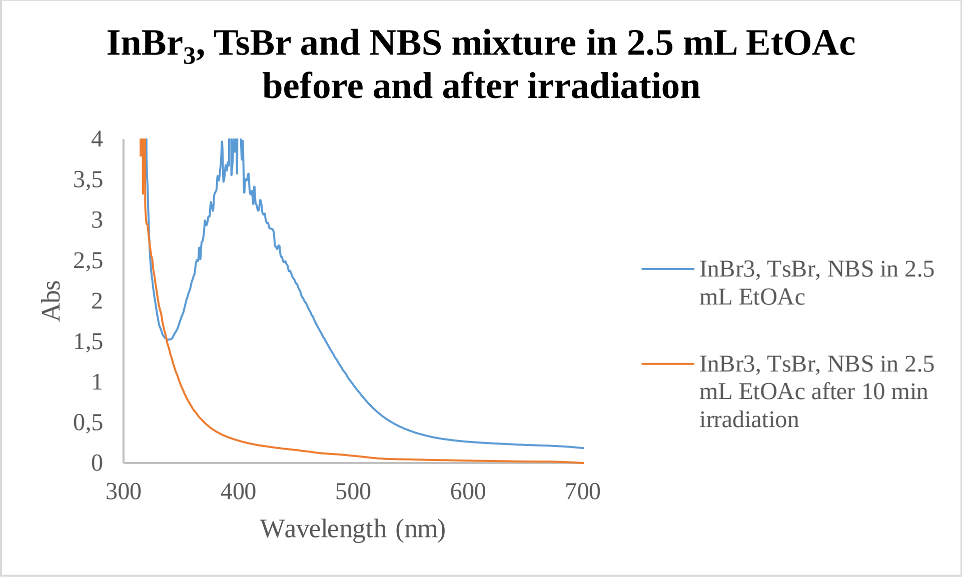

Figure S16. UV-Vis spectra of $InBr_3$ (0.05 mmol), TsBr (0.05 mmol) and NBS (0.05 mmol) mixture in 2.5 mL EtOAc before and after 10 min of irradiation.

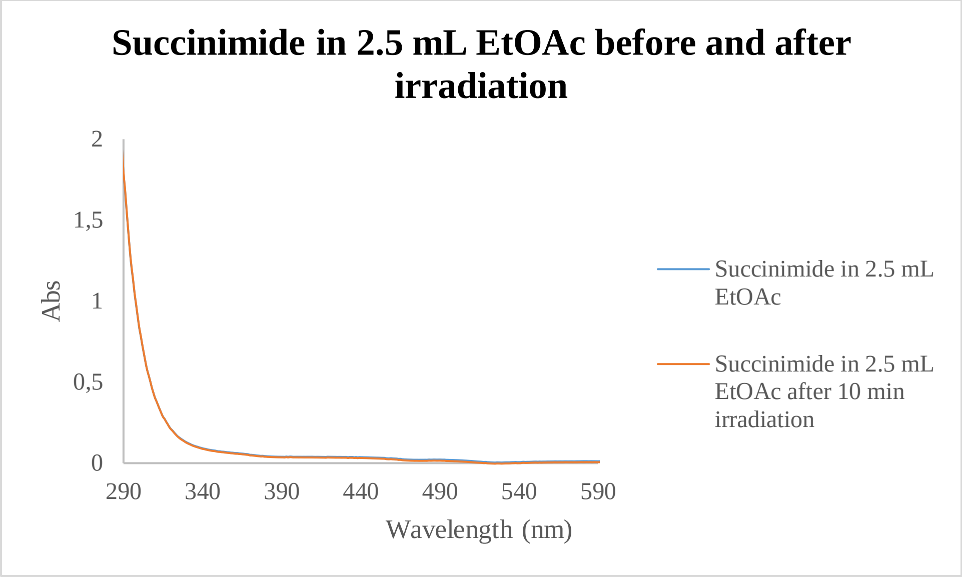

Figure S17. UV-Vis spectra of succinimide (0.05 mmol) in 2.5 mL EtOAc before and after 10 min of irradiation.

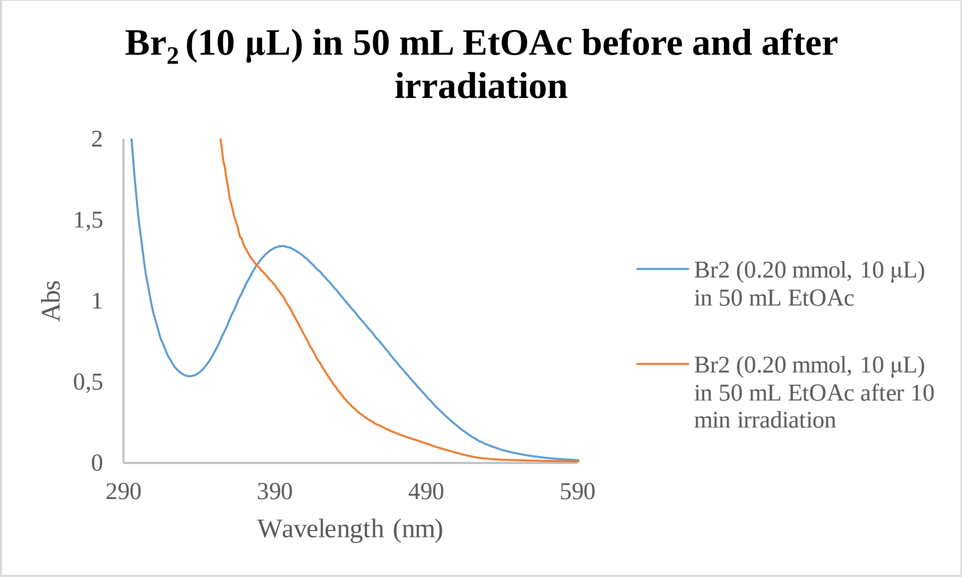

Figure S18. UV-Vis spectra of Br_2 (0.20 mmol, 10 µL) in 50 mL EtOAc before and after irradiation.

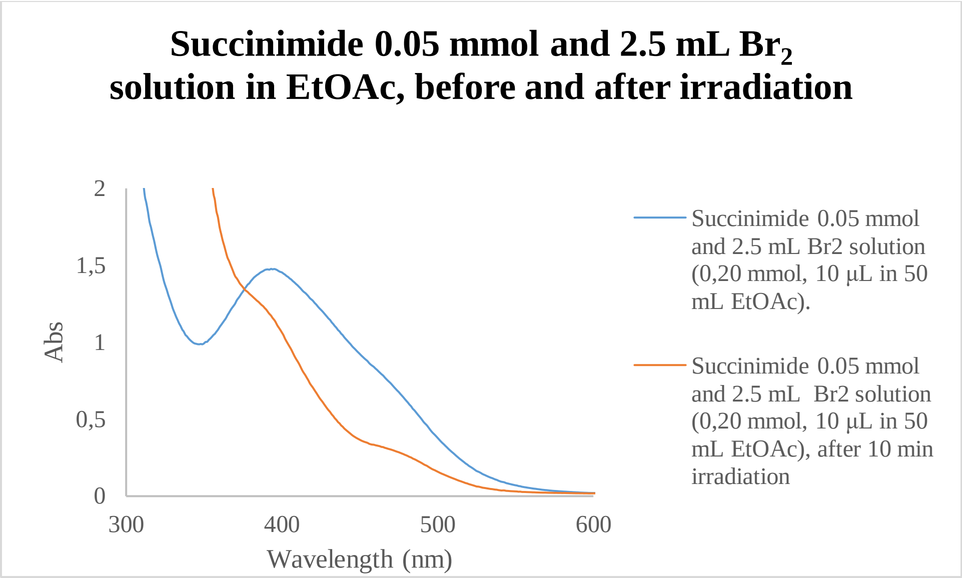

Figure S19. UV-Vis spectra of succinimide (0.05 mmol) and 2.5 mL Br_2 solution (0.20 mmol, 10 μL in 50 mL EtOAc) before and after 10 min irradiation.

Mechanistic Studies

NMR Spectra Studies

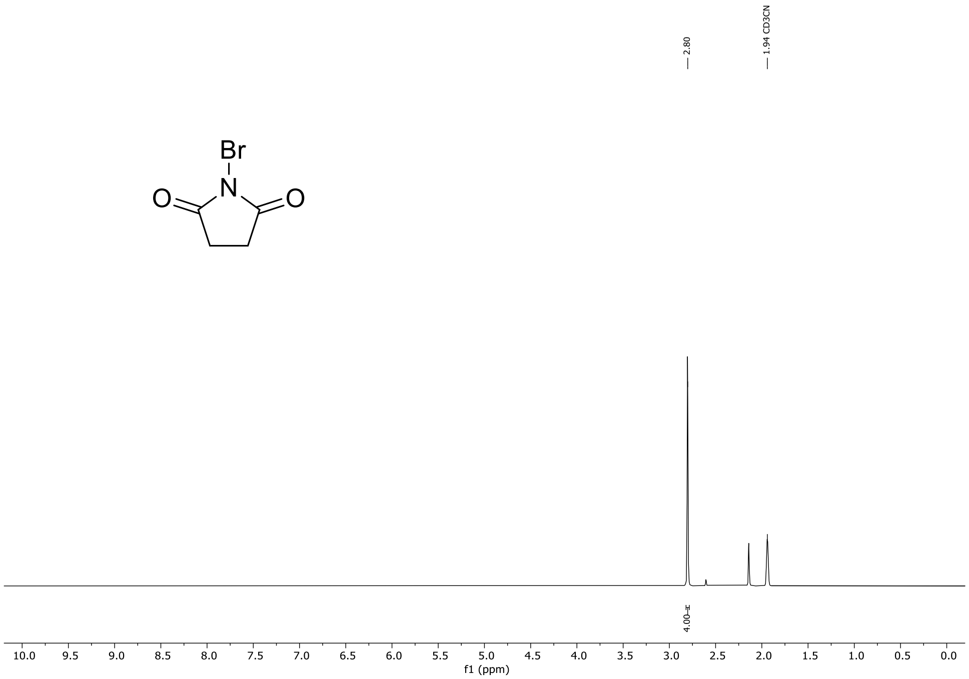

^{1}H NMR spectrum (400 MHz, acetonitrile-*d$_3$*) of NBS (0.1M) in CD_3CN.

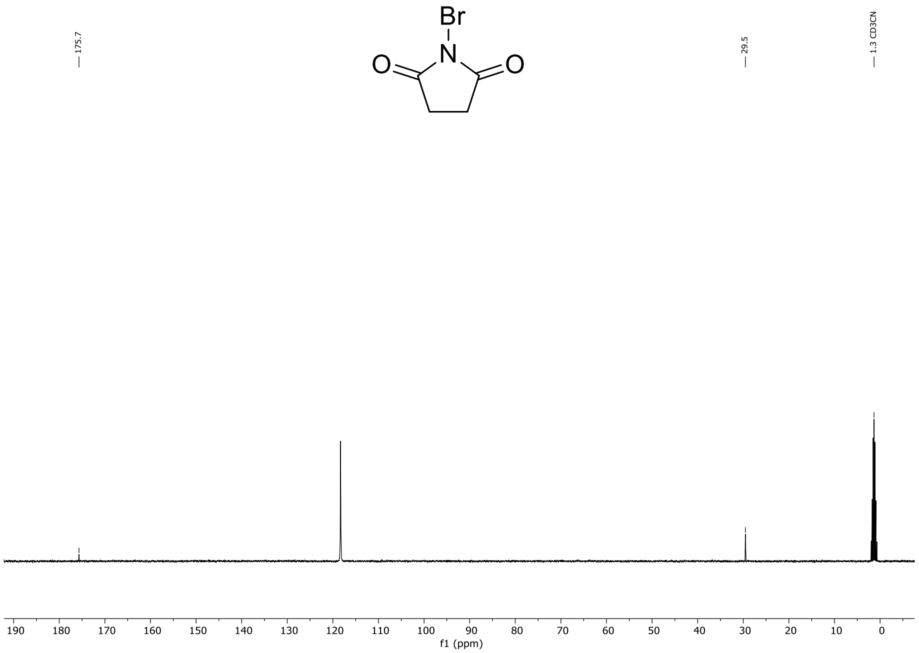

^{13}C NMR spectrum (100 MHz, acetonitrile-*d$_3$*) of NBS (0.1M) in CD_3CN.

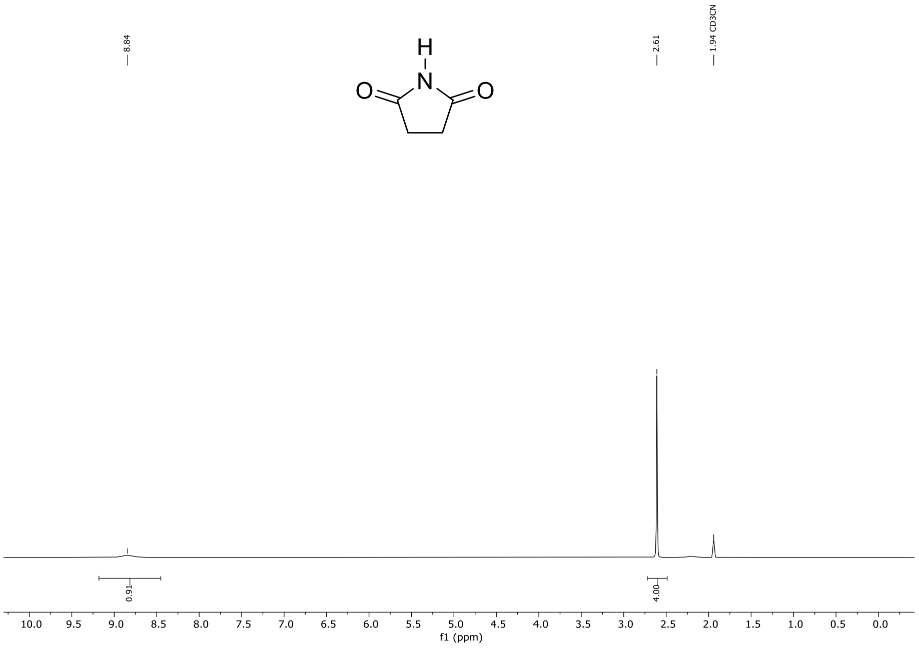

^{1}H NMR spectrum (400 MHz, acetonitrile-*d₃*) of succinimide (0.2M) in CD$_3$CN.

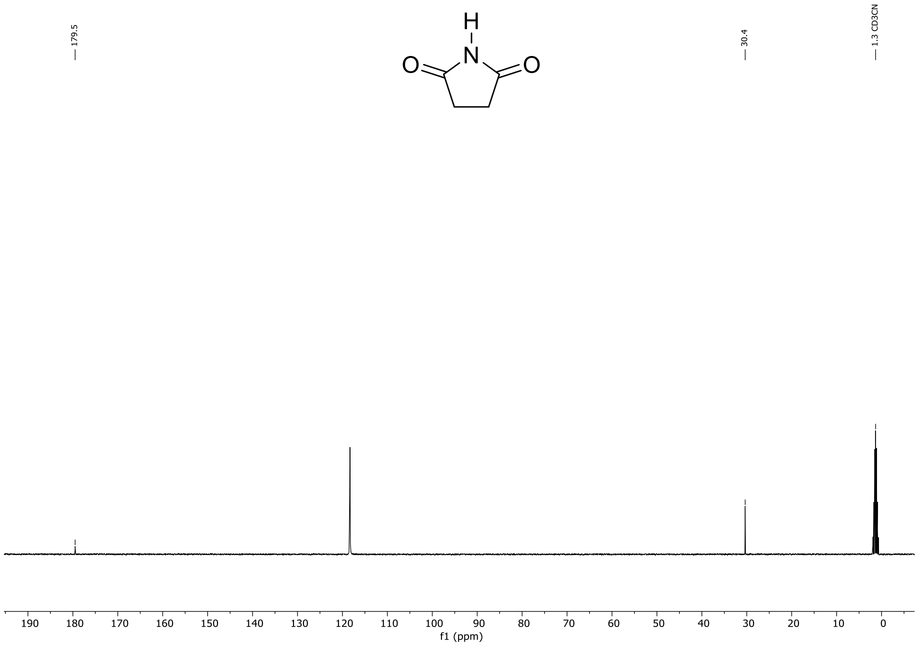

^{13}C NMR spectrum (100 MHz, acetonitrile-*d₃*) of succinimide (0.2M) in CD$_3$CN.

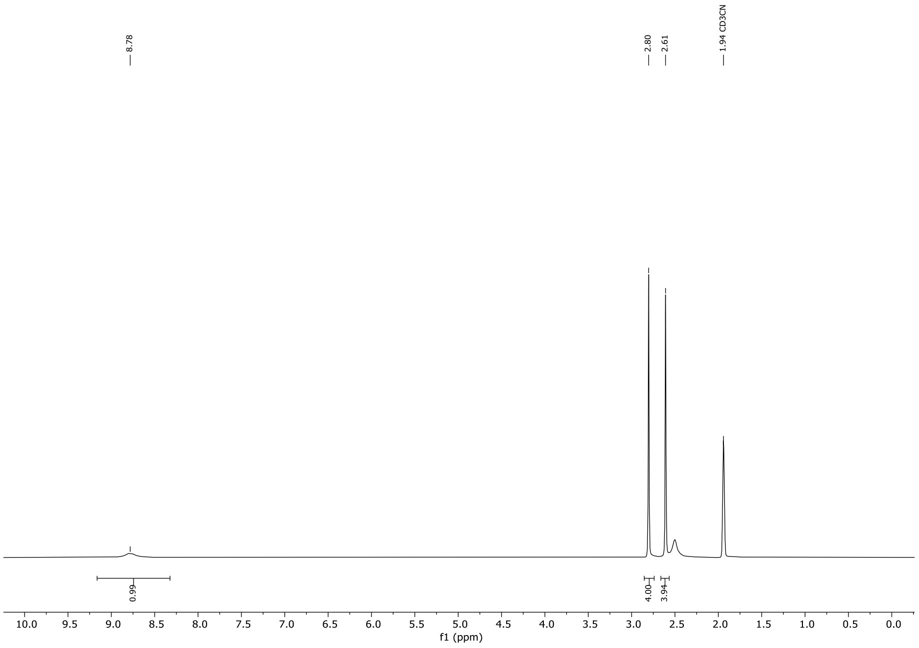

^{1}H NMR spectrum (400 MHz, acetonitrile-*d_3*) of NBS (0.1M) and $InBr_3$ (0.025M) in CD_3CN. The peak at 2.80 ppm corresponds to NBS (4H), while the new peak at 2.61 ppm corresponds to succinimide (4H), formed after the addition of $InBr_3$ in the NBS solution. By the integration, we observe a ratio of NBS:succinimide 1:0.99.

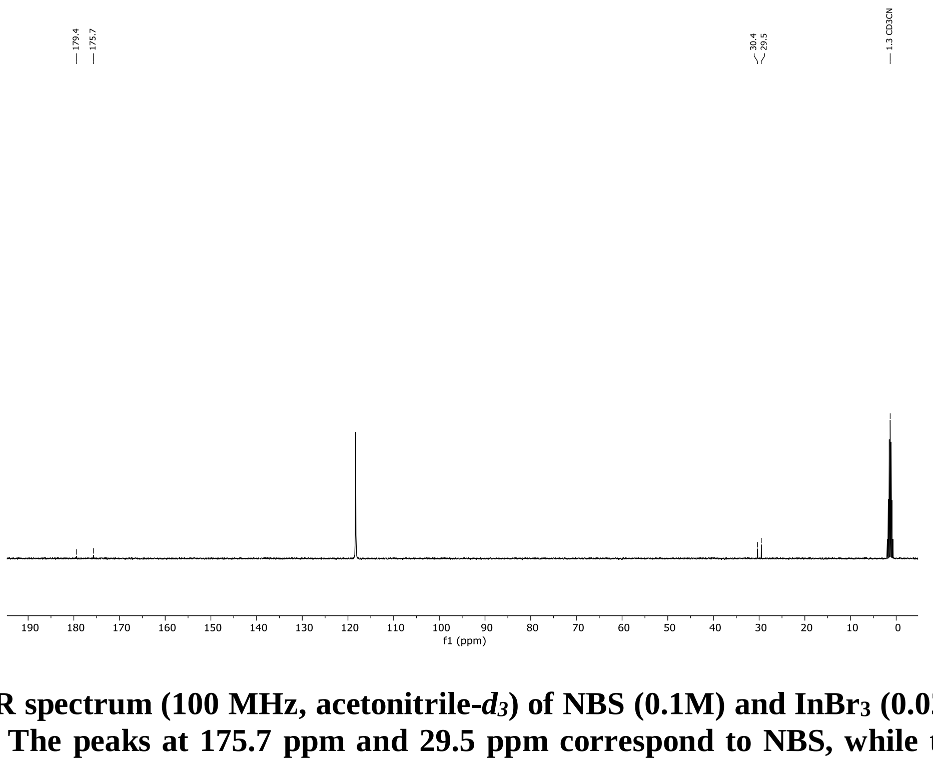

^{13}C NMR spectrum (100 MHz, acetonitrile-*d_3*) of NBS (0.1M) and $InBr_3$ (0.025M) in CD_3CN. The peaks at 175.7 ppm and 29.5 ppm correspond to NBS, while the new peaks at 179.4 ppm and 30.4 ppm correspond to succinimide, formed after the addition of $InBr_3$ in the NBS solution.

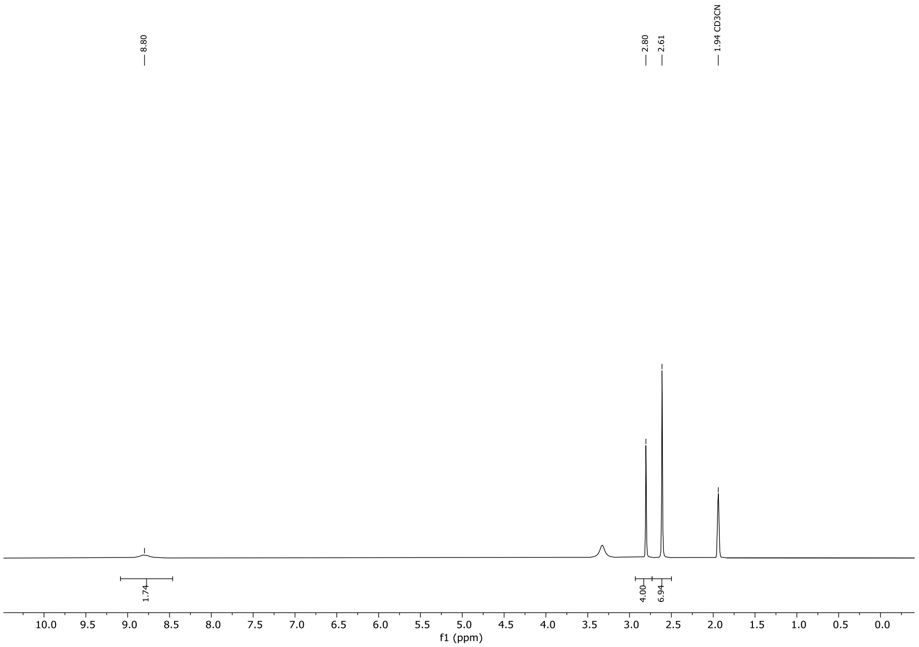

^{1}H NMR spectrum (400 MHz, acetonitrile-*d_3*) of NBS (0.1M) and $InBr_3$ (0.050M) in CD_3CN. By the integration, we observe a ratio of NBS:succinimide 1:1.74.

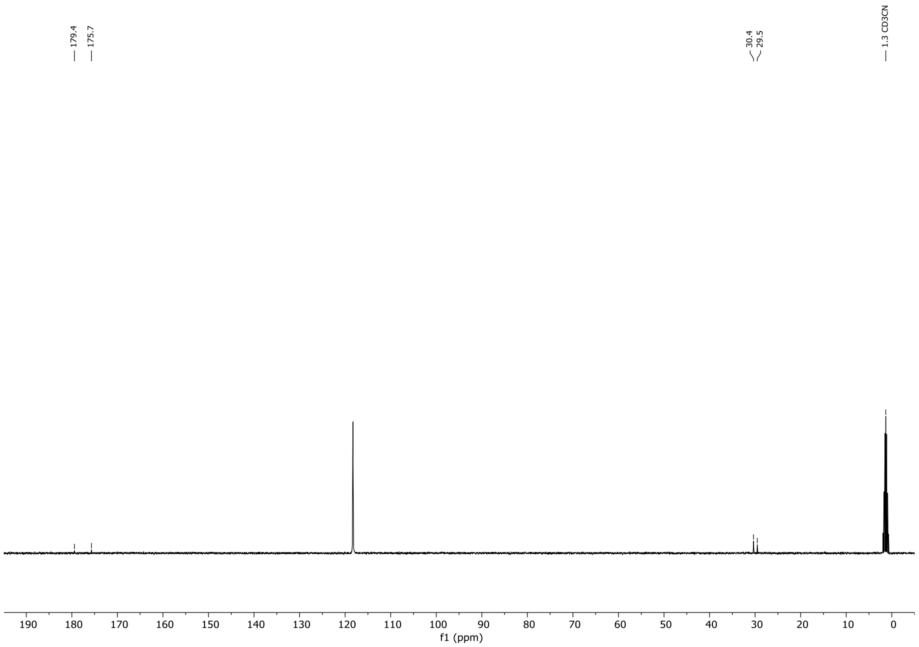

^{13}C NMR spectrum (100 MHz, acetonitrile-*d_3*) of NBS (0.1M) and $InBr_3$ (0.050M) in CD_3CN.

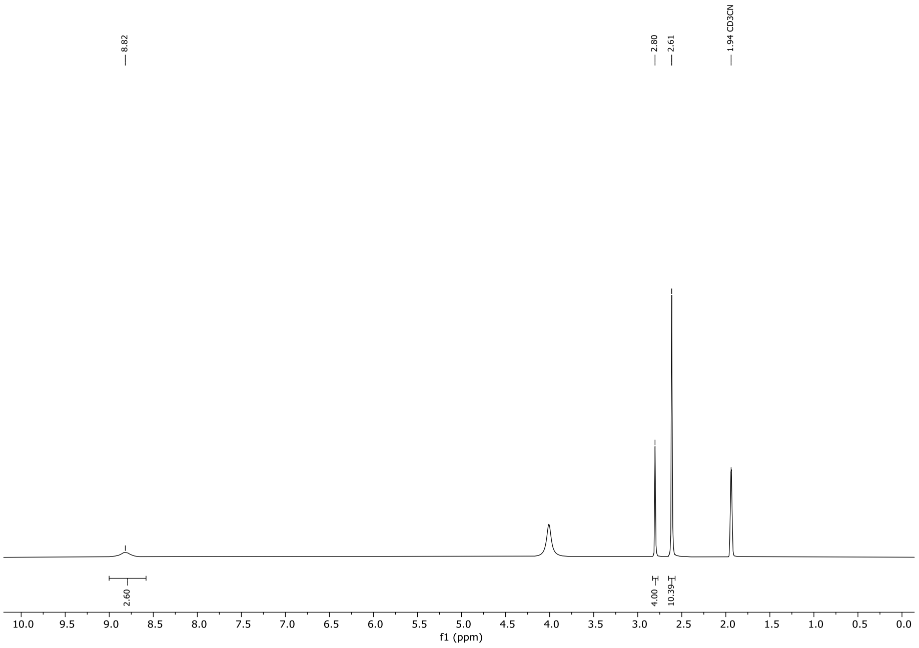

^{1}H NMR spectrum (400 MHz, acetonitrile-*d3*) of NBS (0.1M) and $InBr_3$ (0.075M) in CD_3CN. By the integration, we observe a ratio of NBS:succinimide 1:2.60.

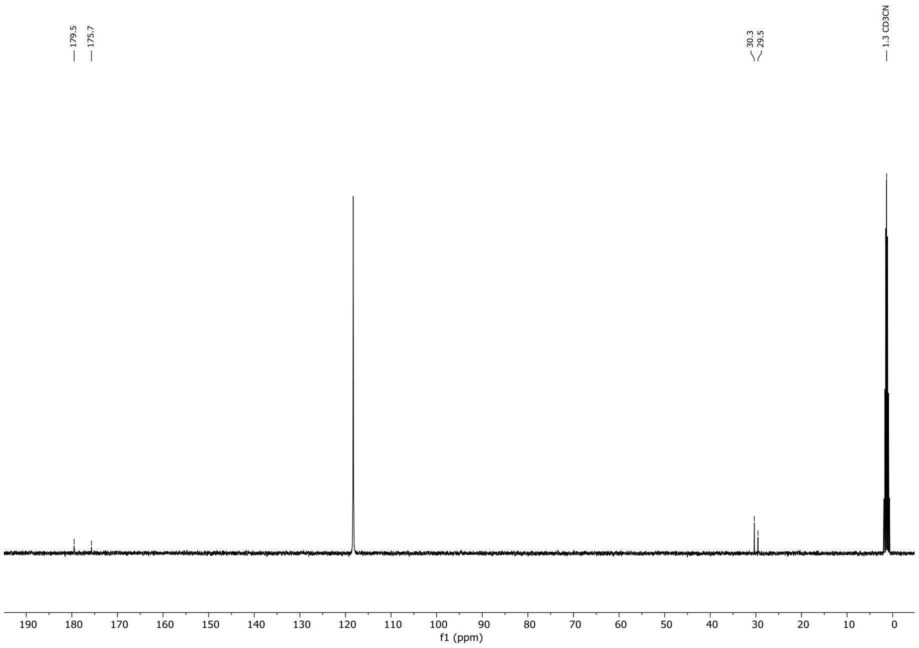

^{13}C NMR spectrum (100 MHz, acetonitrile-*d3*) of NBS (0.1M) and $InBr_3$ (0.075M) in CD_3CN.

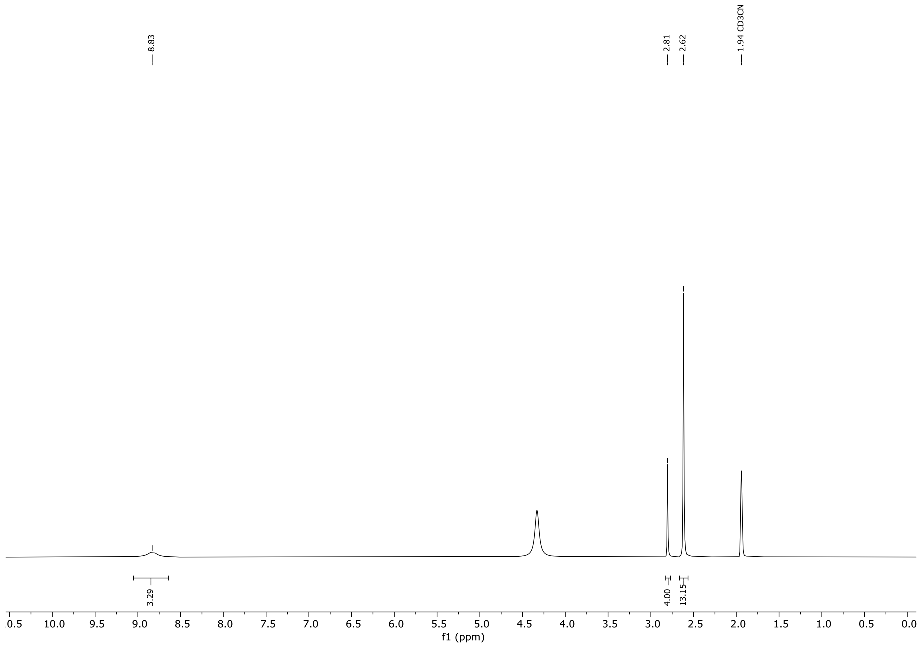

^{1}H NMR spectrum (400 MHz, acetonitrile-*d_3*) of NBS (0.1M) and $InBr_3$ (0.1M) in CD_3CN. By the integration, we observe a ratio of NBS:succinimide 1:3.29.

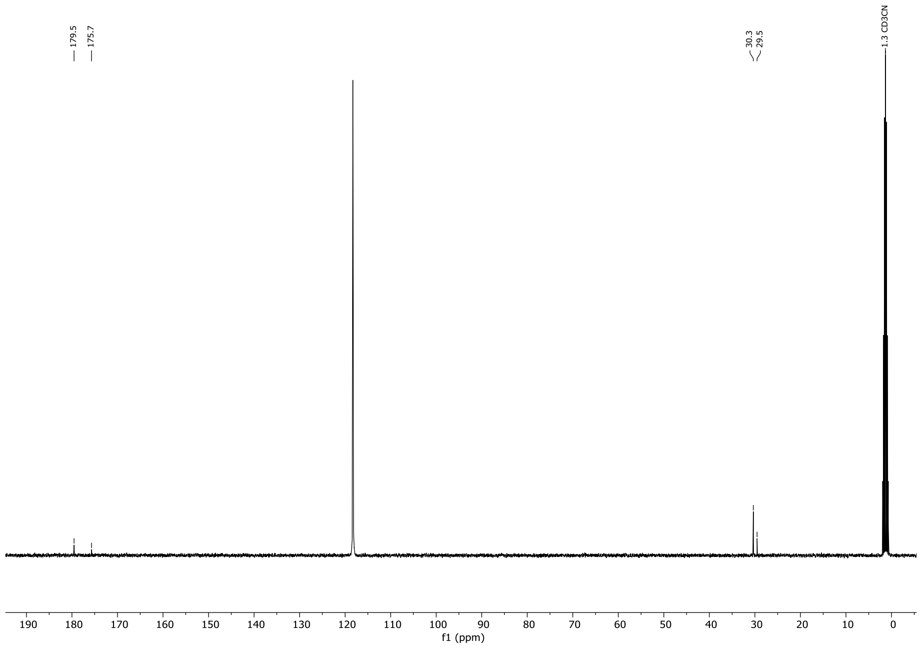

^{13}C NMR spectrum (100 MHz, acetonitrile-*d_3*) of NBS (0.1M) and $InBr_3$ (0.1M) in CD_3CN.

Further Functionalization Reactions of Benzoic Acid

Synthesis of Benzoyl Chloride (6)[3]

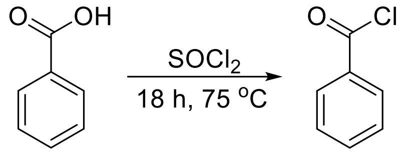

1 **6**

In a dry round-bottom flask, benzoic acid (**1**) (244 mg, 2.0 mmol, 1.00 equiv.), freshly obtained by acid-base washes from the photochemical aerobic upcycling of polystyrene, was introduced, followed by the addition of thionyl chloride (0.7 mL, 10.0 mmol, 5.00 equiv.). The resulting reaction mixture was then placed under reflux conditions and maintained overnight at a temperature of 75 °C under vigorous stirring. Upon completion of the reflux period, the mixture was allowed to cool to room temperature. Any excess of unreacted thionyl chloride was subsequently removed by rotary evaporation under reduced pressure, yielding benzoyl chloride (**6**) without further purification. Colorless liquid; Yield: 95%; **^{1}H NMR** (400 MHz, $CDCl_3$) δ: 8.11 (2H, d, J = 7.9 Hz, ArH), 7.68 (1H, t, J = 7.9 Hz, ArH), 7.51 (2H, t, J = 7.9 Hz, ArH); **^{13}C NMR** (100 MHz, $CDCl_3$) δ: 168.3, 135.3, 133.2, 131.3, 128.9.

General Procedure for the Synthesis of Esters from Benzoyl Chloride and Alcohols

In a round-bottom flask, the corresponding alcohol (0.36 mmol, 1.00 equiv.), triethylamine (75.3 μL, 0.54 mmol, 1.50 equiv.), and DMAP (5 mg, 0.04 mmol, 0.10 equiv.) were dissolved in dichloromethane (3.6 mL) and cooled at 0 °C. Benzoyl chloride (**6**) (56 mg, 0.40 mmol, 1.10 equiv.) was then added dropwise, and the reaction mixture was stirred at r.t., until complete consumption of the starting material, as monitored by TLC (8-18 h). Upon reaction completion, the reaction mixture was extracted with dichloromethane (10 mL) and washed with aqueous HCl 1 N (10 mL). The organic layer

was subsequently washed with brine (2 x 10 mL), dried over Na_2SO_4, filtered and concentrated under reduced pressure. The crude product was purified by column chromatography on silica gel.

(3*S*,8*S*,9*S*,10*R*,13*R*,14*S*,17*R*)-10,13-Dimethyl-17-((*R*)-6-methylheptan-2-yl)-2,3,4,7,8,9,10,11,12,13,14,15,16,17-tetradecahydro-1*H*-cyclopenta[*a*]phenanthren-3-yl benzoate (7)[4a]

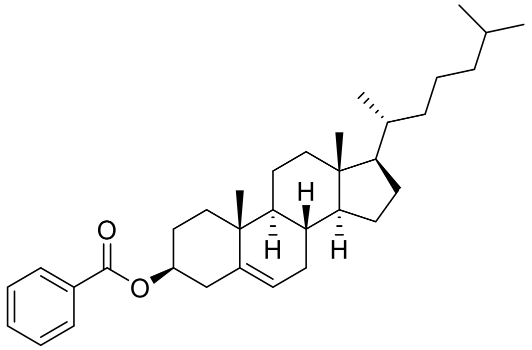

Pearl-white solid; Yield: 76**%**; **m.p.** 135-137 °C (lit. m.p.: 136-140 °C); $[\alpha]_D^{20}$ = −14.0 (c 1.00, $CHCl_3$), $[\alpha]_D^{25}$ lit.[4b] = −13.7 (c 0.90, $CHCl_3$); **^{1}H NMR (400 MHz, $CDCl_3$)** δ 8.05 (2H, d, *J* = 7.6 Hz, ArH), 7.54 (1H, t, *J* = 7.6 Hz, ArH), 7.43 (2H, t, *J* = 7.6 Hz, ArH), 5.43 (1H, d, *J* = 5.0 Hz, =CH), 4.93-4.82 (1H, m, OCH), 2.48 (2H, d, *J* = 8.1 Hz, 2 x C*H*H), 2.07-1.96 (3H, m, 3 x C*H*H), 1.95-1.89 (1H, m, C*H*H), 1.88-1.78 (1H, m, C*H*H), 1.77-1.68 (1H, m, C*H*H), 1.63-1.45 (6H, m, 6 x C*H*H), 1.41-1.31 (3H, m, C*H*H and CH), 1.30-1.10 (7H, m, C*H*H and CH), 1.08 (3H, s, CH_3), 1.06-0.96 (4H, m, C*H*H and CH), 0.93 (3H, d, *J* = 6.5 Hz, CHCH_3), 0.88 (6H, d, *J* = 6.5 Hz, 2 x CHCH_3), 0.70 (3H, s, CH_3); **^{13}C NMR (100 MHz, $CDCl_3$)** δ 165.9, 139.6, 132.6, 130.8, 129.5, 128.2, 122.7, 74.5, 56.7, 56.1, 50.0, 42.3, 39.7, 39.5, 38.2, 37.0, 36.6, 36.2, 35.8, 31.9, 31.8, 28.2, 28.0, 27.9, 24.3, 23.8, 22.8, 22.5, 21.0, 19.3, 18.7, 11.8; **MS (ESI)** 491 $[M+H]^+$.

(3*S*,8*R*,9*S*,10*S*,13*S*,14*S*)-10,13-Dimethyl-17-oxohexadecahydro-1*H*-cyclopenta[*a*]phenanthren-3-yl benzoate (8)[5a]

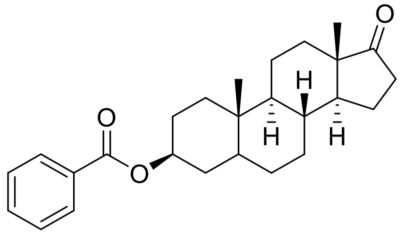

White solid; Yield: 79**%**; **m.p.** 208-211 ºC (lit. m.p.: 216 ºC)[5b]; $[\alpha]_D^{20}$ = 68 (c 1.00, $CHCl_3$), $[\alpha]_D^{20}$ lit.[5b] = 69.0 (c 1.44, $CHCl_3$); **^{1}H NMR (400 MHz, $CDCl_3$)** δ 8.01 (2H, d, *J* = 7.6 Hz, ArH), 7.51 (1H, t, *J* = 7.6 Hz, ArH), 7.40 (2H, t, *J* = 7.6 Hz, ArH), 4.97-4.87 (1H, m, OCH), 2.40 (1H, dd, *J* = 19.2 and 8.8 Hz, C*H*H), 2.09-1.98 (1H, m, C*H*H), 1.97-1.86 (2H, m, 2 x C*H*H), 1.81-1.70 (4H, m, 4 x C*H*H), 1.69-1.59 (2H, m, 2 x C*H*H), 1.56-1.40 (3H, m, 3 x C*H*H), 1.37-1.18 (6H, m, 5 x C*H*H and CH), 1.13-1.04 (1H, m, CH), 1.03-0.91 (1H, m, CH), 0.87 (3H, s, CH_3), 0.83 (3H, s, CH_3), 0.76-0.67 (1H, m, CH); **^{13}C NMR (100 MHz, $CDCl_3$)** δ 221.3, 166.1, 132.7, 130.9, 129.5, 128.2, 74.1, 54.3, 51.4, 47.8, 44.7, 36.7, 35.8, 35.7, 35.0, 34.0, 31.5, 30.8, 28.3, 27.5, 21.8, 20.5, 13.8, 12.3; **MS (ESI)** 395 $[M+H]^+$.

(1*S*,2*R*,4*S*)-1,7,7-Trimethylbicyclo[2.2.1]heptan-2-yl benzoate (9)[6a]

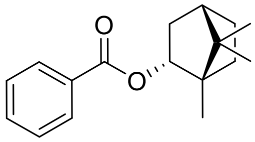

Colorless oil; Yield: 59**%**; $[\alpha]_D^{20}$ = −38 (c 1.00, ethanol), $[\alpha]_D^{15}$ lit.[6b] = -42.7 (c 1.69, ethanol); **^{1}H NMR (400 MHz, $CDCl_3$)** δ 8.07 (2H, d, *J* = 8.0 Hz, ArH), 7.56 (1H, t, *J* = 8.0 Hz, ArH), 7.45 (2H, t, *J* = 8.0 Hz, ArH), 5.12 (1H, d, *J* = 10.0 Hz, OCH), 2.55-2.42 (1H, m, C*H*H), 2.20-2.09 (1H, m, C*H*H), 1.86-1.72 (2H, m, 2 x C*H*H), 1.46-1.37 (1H, m, CH), 1.35-1.28 (1H, m, C*H*H), 1.15-1.10 (1H, m, C*H*H), 0.97 (3H, s, CH_3), 0.92 (6H, s, 2 x CH_3); **^{13}C NMR (100 MHz, $CDCl_3$)** δ 166.8, 132.7, 130.9, 129.5, 128.3, 80.5, 49.1, 47.9, 45.0, 36.9, 28.1, 27.4, 19.7, 18.9, 13.6; **MS (ESI)** 281 $[M+Na]^+$.

Synthesis of Benzoic Peroxyanhydride (10)[7a]

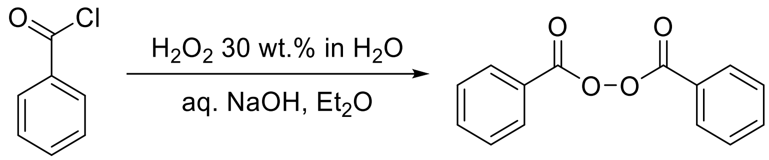

In a round-bottom flask, a solution of benzoyl chloride (**6**) (29.5 mg, 0.21 mmol, 1.0 equiv.) in diethyl ether (0.1 mL) was cooled at 0 °C. Hydrogen peroxide (13.0 μL, 30 wt.% in H_2O, 0.13 mmol, 0.6 equiv.) was then added dropwise over 5 minutes. Subsequently, an aqueous solution of NaOH (11 mg in 0.1 mL H_2O, 0.27 mmol, 1.3 equiv.) was introduced dropwise over 5 minutes. The reaction mixture was left stirring for another 30 minutes. The resulting white precipitate was collected by filtration, washed with water (2 x 1 mL) and diethyl ether (3 x 1 mL), and recrystallized from a cold acetone/water mixture (v/v 1:3) to afford pure benzoyl peroxide (**10**). White solid; Yield: 71**%**; m.p.: 100-103 °C (lit. m.p.: 105-105.5 °C)[7b]; **^{1}H NMR** (400 MHz, $CDCl_3$) δ: 8.09 (4H, d, J = 7.7 Hz, ArH), 7.67 (2H, t, J = 7.7 Hz, ArH), 7.52 (4H, t, J = 7.7 Hz, ArH); **^{13}C NMR** (100 MHz, $CDCl_3$) δ: 163.1, 134.3, 129.8, 128.9, 125.6; **MS (ESI)** 265 $[M+Na]^+$.

References

1. T. E. Hurst, J. A. Deichert, L. Kapeniak, R. Lee, J. Harris, P. G. Jessop, V. Snieckus, *Org. Lett.* **2019**, *21*, 3882-3885.
2. N. F. Nikitas, E. Skolia, P. L. Gkizis, I. Triandafillidi, C. G. Kokotos, *Green Chem.* **2023**, *25*, 4750-4759.
3. S. Cailotto, M. Negrato, S. Daniele, R. Luque, M. Selva, E. Amadio, A. Perosa, *Green Chem.* **2020**, *22*, 1145-1149.
4. a) A. Modak, T. Naveen, D. Maiti, *Chem. Commun.* **2013**, *49*, 252-254; b) Y. Inoue, S. Takamuku, Y. Kunitomi, H. Sakurai, *J. Chem. Soc., Perkin Trans. 2* **1980**, 1672-1677.
5. a) S. Kamijo, K. Tao, G. Takao, H. Tonoda, T. Murafuji, *Org. Lett.* **2015**, *17*, 3326-3329; b) D. H. R. Barton, J. D. Cox, *J. Chem. Soc.* **1948**, 783-793.
6. a) B. Lyu, Y. Hiraga, R. Takagi, S. Niwayama, *Molecules* **2025**, *30*, 597; b) N. Kunieda, A. Suzuki, M. Kinoshita, *Bull. Chem. Soc. Jpn.* **1981**, *54*, 1143-1150.
7. a) N. Yadav, S. R. Bhatta, J. N. Moorthy, *J. Org. Chem.* **2023,** *88*, 5431-5439; b) F. D. Greene, J. Kazan, *J. Org. Chem.* **1963**, *28*, 2168-2171.

NMR Spectra

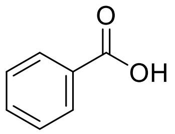

1

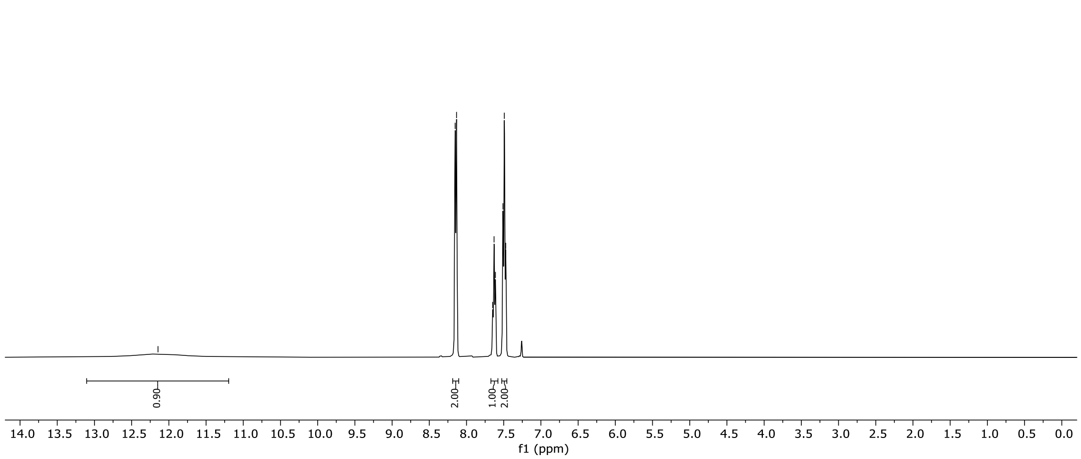

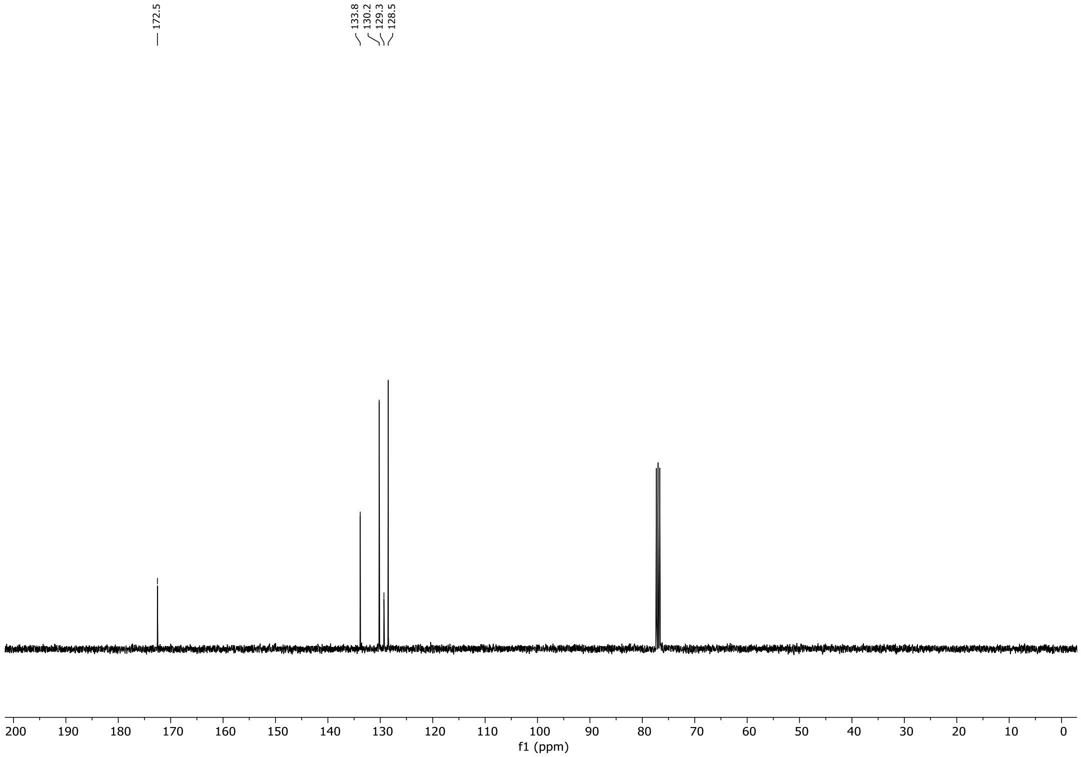

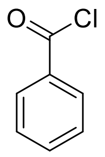
O
Cl

6

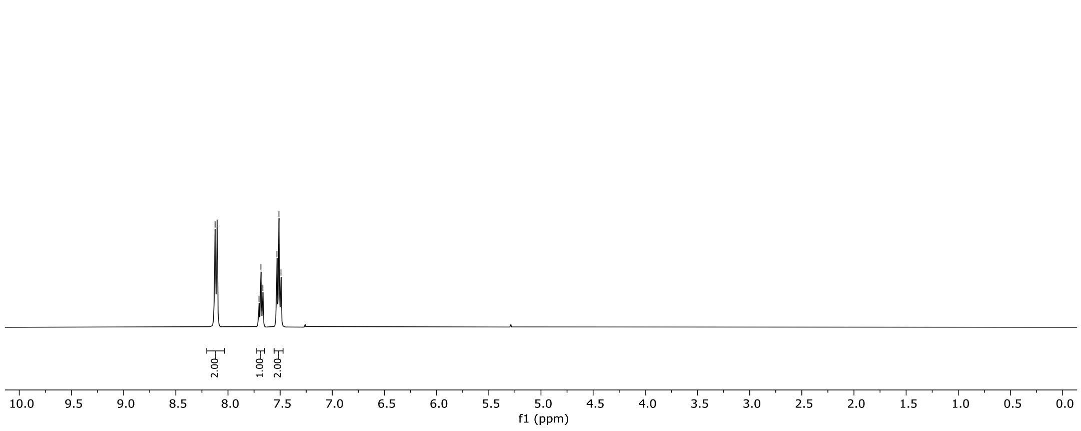
8.12
8.10
7.70
7.68
7.67
7.53
7.51
7.49
2.00
1.00
2.00
f1 (ppm)

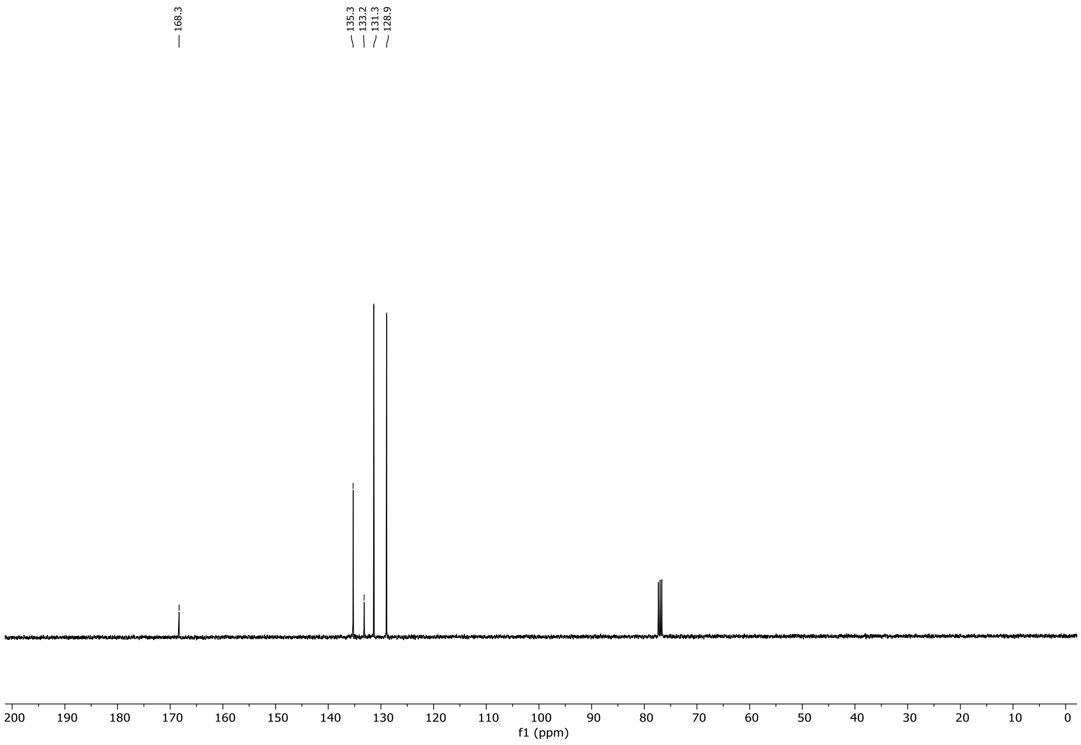
168.3
135.3
133.2
131.3
128.9
f1 (ppm)

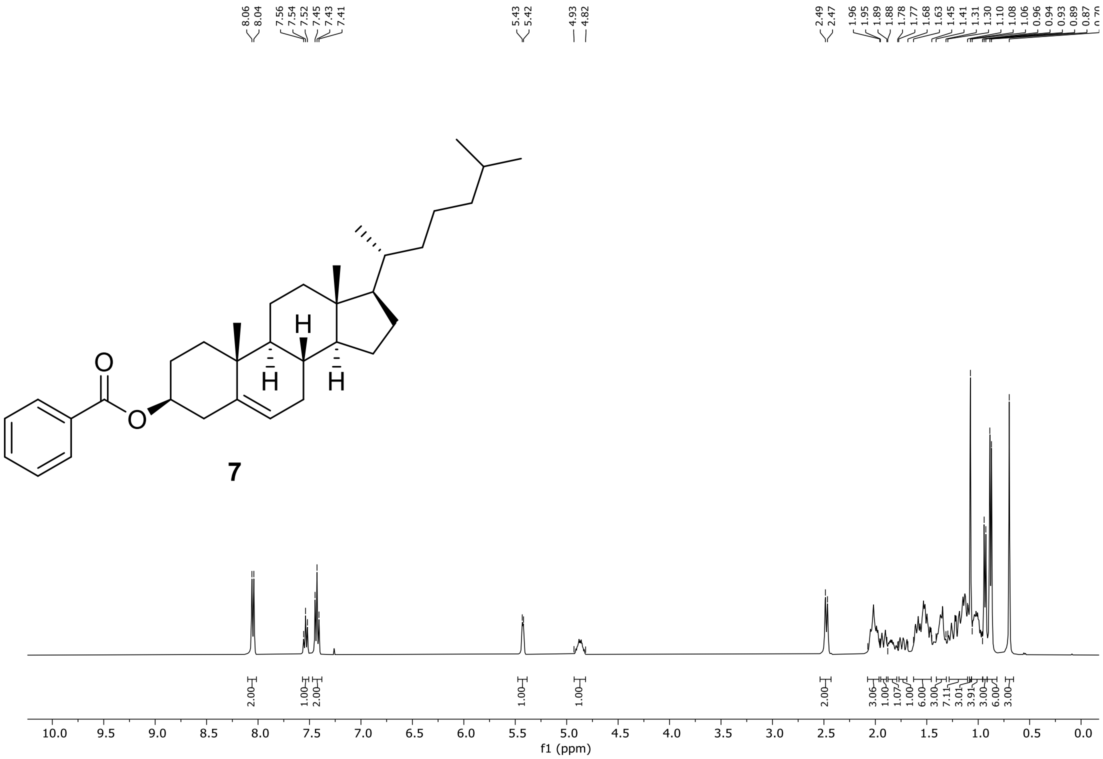

7

8.06
8.04
7.56
7.54
7.52
7.45
7.43
7.41
5.43
5.42
4.93
4.82
2.49
2.47
2.00
1.00
2.00
1.00
1.00
2.00
10.0 9.5 9.0 8.5 8.0 7.5 7.0 6.5 6.0 5.5 5.0 4.5 4.0 3.5 3.0 2.5 2.0 1.5 1.0 0.5 0.0
f1 (ppm)

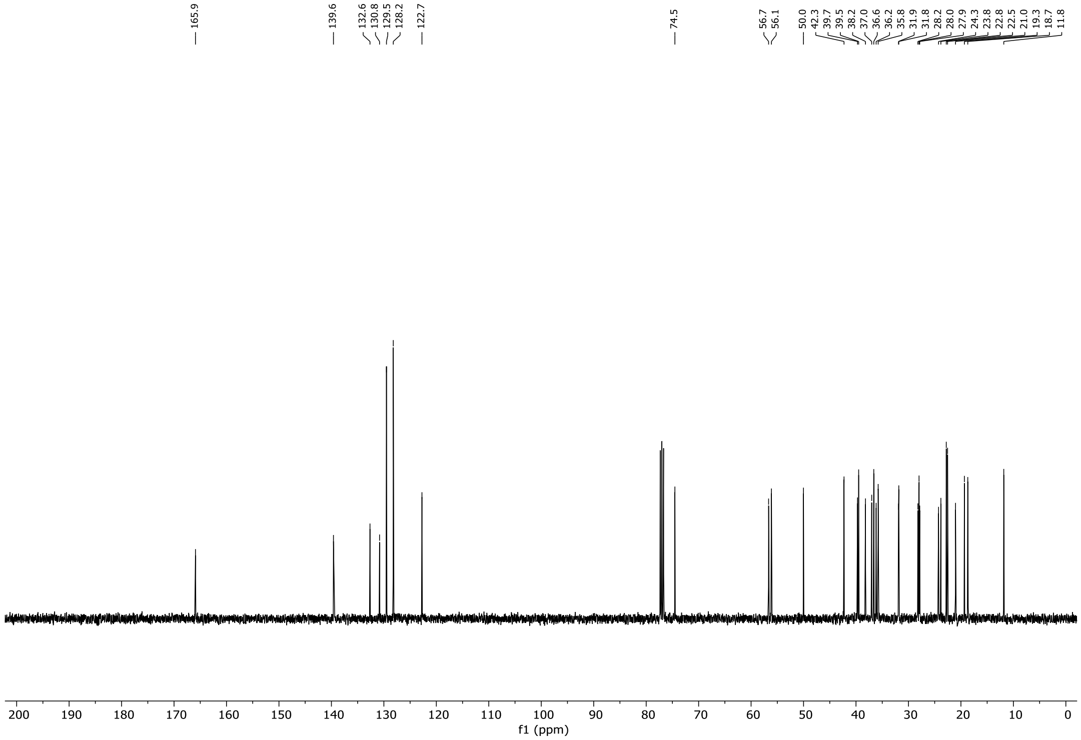
165.9
139.6
132.6
130.8
129.5
128.2
122.7
74.5
56.7
56.1
50.0
42.3
39.7
39.5
38.2
37.0
36.6
36.2
35.8
31.9
31.8
28.2
28.0
27.9
24.3
23.8
22.8
22.5
21.0
19.3
18.7
11.8
200 190 180 170 160 150 140 130 120 110 100 90 80 70 60 50 40 30 20 10 0
f1 (ppm)

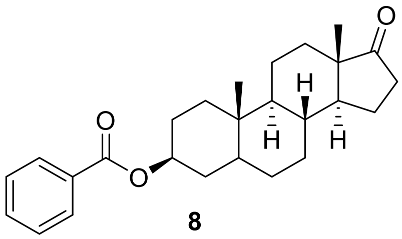

8

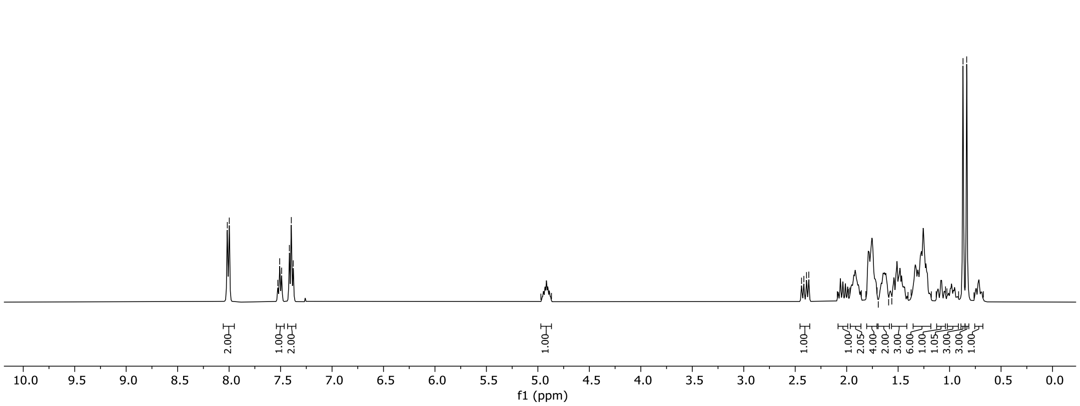

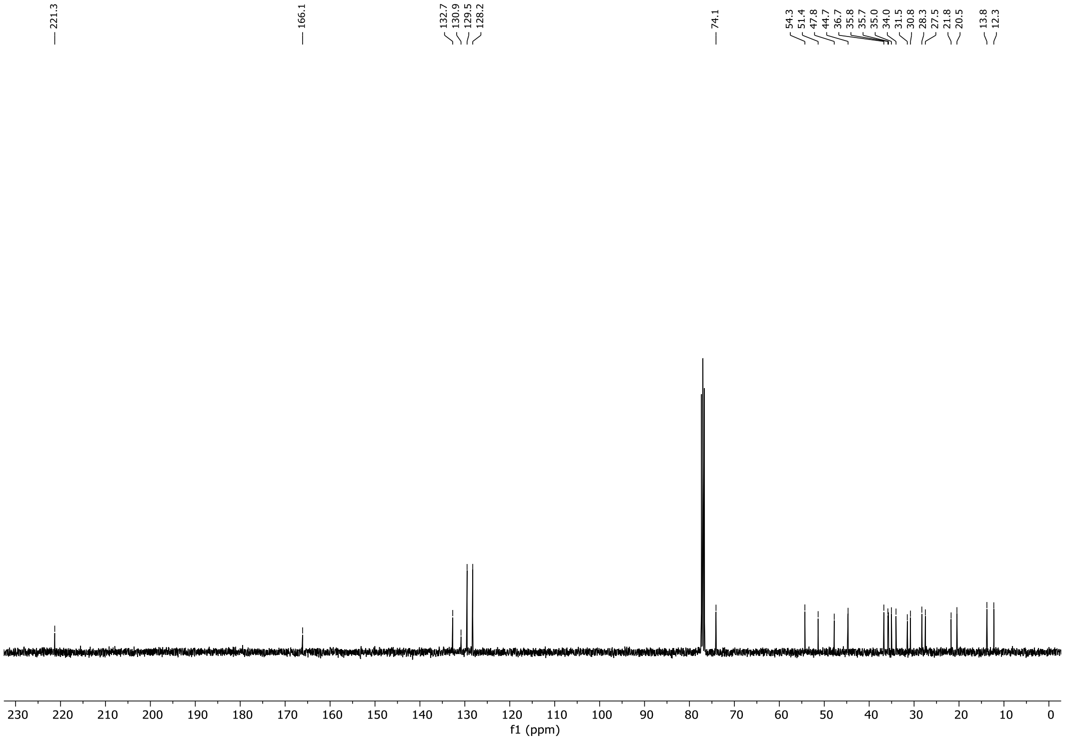

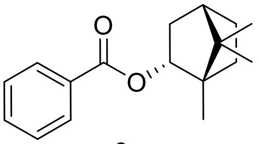
O
O

9

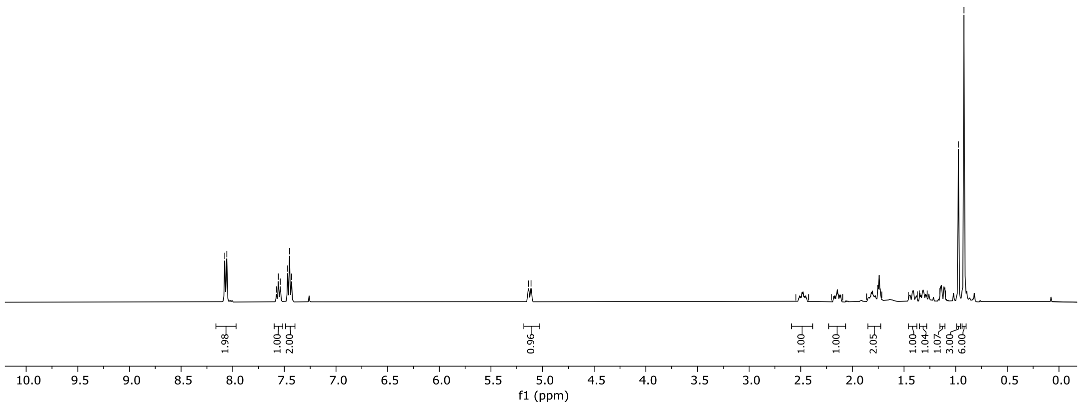
8.08
8.06
7.58
7.56
7.54
7.47
7.45
7.43
5.14
5.11
2.55
2.42
2.20
2.09
1.86
1.72
1.46
1.37
1.35
1.28
1.15
1.10
0.97
0.92
1.98
1.00
2.00
0.96
1.00
1.00
2.05
1.00
1.04
1.07
3.00
6.00
10.0 9.5 9.0 8.5 8.0 7.5 7.0 6.5 6.0 5.5 5.0 4.5 4.0 3.5 3.0 2.5 2.0 1.5 1.0 0.5 0.0
f1 (ppm)

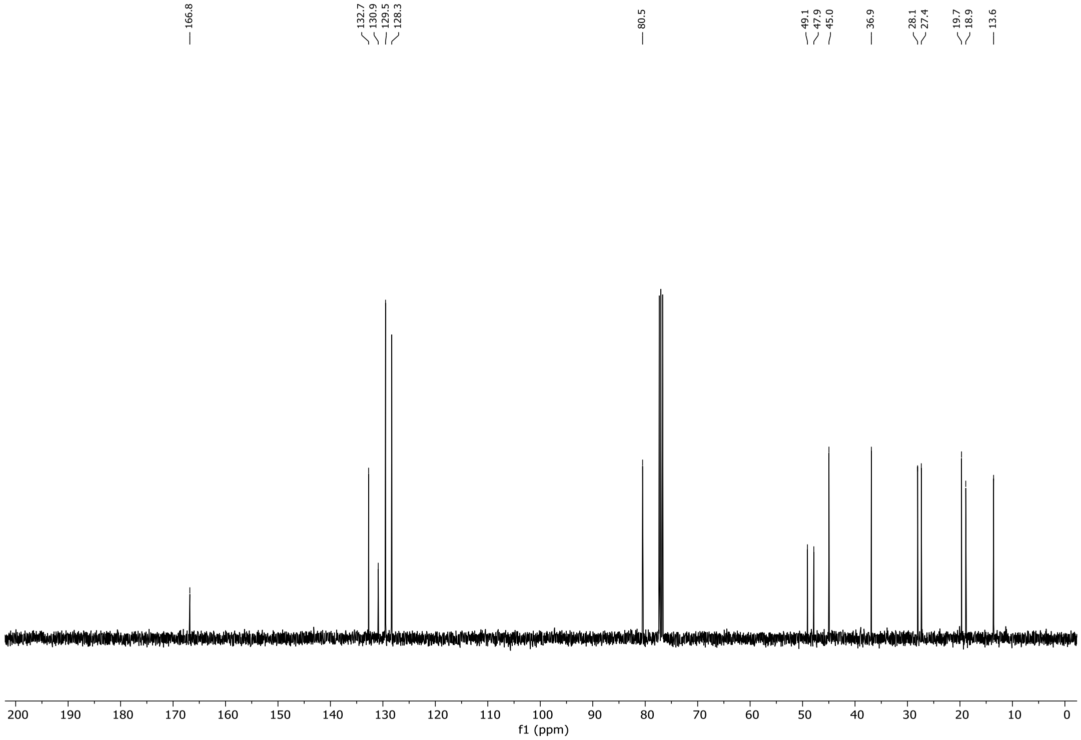
166.8
132.7
130.9
129.5
128.3
80.5
49.1
47.9
45.0
36.9
28.1
27.4
19.7
18.9
13.6
200 190 180 170 160 150 140 130 120 110 100 90 80 70 60 50 40 30 20 10 0
f1 (ppm)

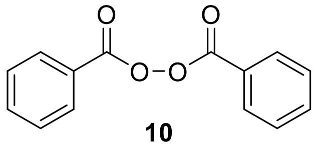

10

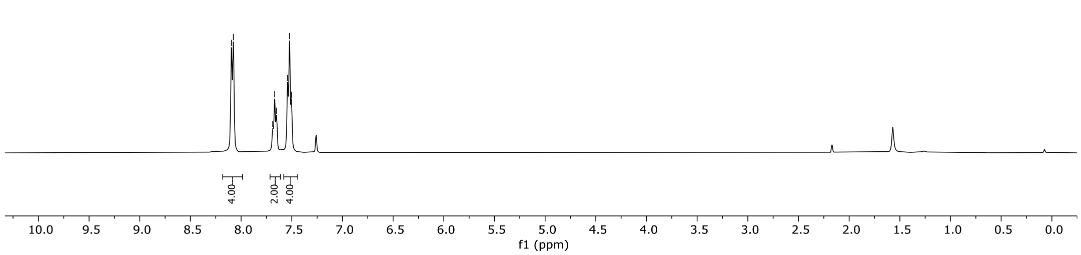
8.10
8.08
7.69
7.67
7.65
7.54
7.52
7.50
4.00
2.00
4.00
10.0 9.5 9.0 8.5 8.0 7.5 7.0 6.5 6.0 5.5 5.0 4.5 4.0 3.5 3.0 2.5 2.0 1.5 1.0 0.5 0.0
f1 (ppm)

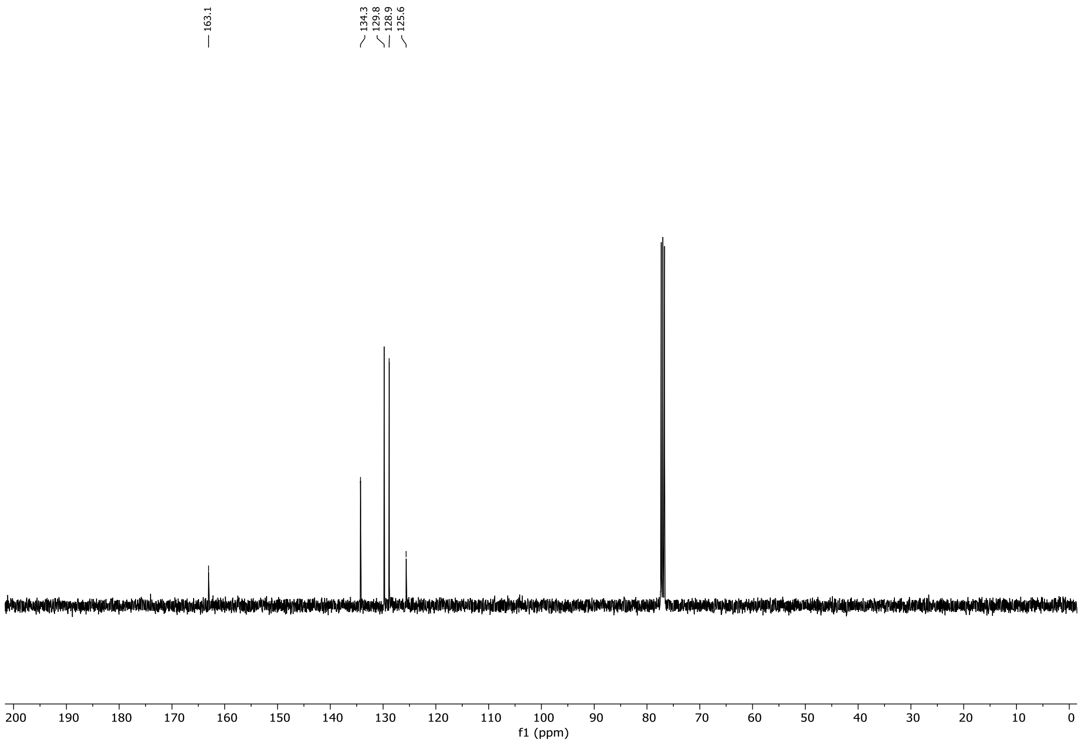
163.1
134.3
129.8
128.9
125.6
200 190 180 170 160 150 140 130 120 110 100 90 80 70 60 50 40 30 20 10 0
f1 (ppm)
